# Safety and immunogenicity of a thermostable ID93 + GLA-SE tuberculosis vaccine candidate in healthy adults

**DOI:** 10.1038/s41467-023-36789-2

**Published:** 2023-03-06

**Authors:** Zachary K. Sagawa, Cristina Goman, Aude Frevol, Azra Blazevic, Janice Tennant, Bridget Fisher, Tracey Day, Stephen Jackson, Franck Lemiale, Leon Toussaint, Irene Kalisz, Joe Jiang, Lisa Ondrejcek, Raodoh Mohamath, Julie Vergara, Alan Lew, Anna Marie Beckmann, Corey Casper, Daniel F. Hoft, Christopher B. Fox

**Affiliations:** 1 Access to Advanced Health Institute (formerly Infectious Disease Research Institute), Seattle, WA USA; 2 Saint Louis University Center for Vaccine Development, St. Louis, MO USA; 3 Advanced Bioscience Laboratories (ABL), Inc., Rockville, MD USA; 4 DF/Net Research, Inc., Seattle, WA USA; 5 Department of Global Health, University of Washington, Seattle, WA USA; 6 Department of Medicine, University of Washington, Seattle, WA USA; 7 Vaccine and Infectious Disease Division, Fred Hutchinson Cancer Center, Seattle, WA USA; 8 Present Address: HDT Bio, Seattle, WA USA; 9 Present Address: Bristol-Myers Squibb, Seattle, WA USA; 10 Present Address: Janssen Vaccines, Leiden, The Netherlands; 11 Present Address: Universal Cells, Seattle, WA USA

**Keywords:** Protein vaccines, Tuberculosis, Phase I trials

## Abstract

Adjuvant-containing subunit vaccines represent a promising approach for protection against tuberculosis (TB), but current candidates require refrigerated storage. Here we present results from a randomized, double-blinded Phase 1 clinical trial (NCT03722472) evaluating the safety, tolerability, and immunogenicity of a thermostable lyophilized single-vial presentation of the ID93 + GLA-SE vaccine candidate compared to the non-thermostable two-vial vaccine presentation in healthy adults. Participants were monitored for primary, secondary, and exploratory endpoints following intramuscular administration of two vaccine doses 56 days apart. Primary endpoints included local and systemic reactogenicity and adverse events. Secondary endpoints included antigen-specific antibody (IgG) and cellular immune responses (cytokine-producing peripheral blood mononuclear cells and T cells). Both vaccine presentations are safe and well tolerated and elicit robust antigen-specific serum antibody and Th1-type cellular immune responses. Compared to the non-thermostable presentation, the thermostable vaccine formulation generates greater serum antibody responses (*p* < 0.05) and more antibody-secreting cells (*p* < 0.05). In this work, we show the thermostable ID93 + GLA-SE vaccine candidate is safe and immunogenic in healthy adults.

## Abstract (web summary)

Here the authors present results from a randomized, double-blinded Phase 1 clinical trial, testing a thermostable presentation of a clinical-stage adjuvanted subunit tuberculosis vaccine candidate. The vaccine candidate is safe and well tolerated, and elicits comparable or improved immune responses compared to the non-thermostable presentation.

## Introduction

Tuberculosis (TB) is a leading infectious cause of morbidity and mortality, having killed 1.5 million people and caused 10 million new infections globally in 2020. Two-thirds of new cases occur in eight countries (India, China, Indonesia, Philippines, Pakistan, Nigeria, Bangladesh, and South Africa)^1^. For over 100 years, only one vaccine has been widely distributed for the prevention of TB disease (Bacillus Calmette-Guérin, or BCG). This vaccine has limited efficacy in the prevention of TB disease, being most useful for the prevention of TB meningitis and miliary disease in young children^2^. The effectiveness of the vaccine in adults or for preventing pulmonary disease is more modest^3^, and BCG is occasionally limited in availability^4^.

Efforts to develop new TB vaccines have most often relied on the rational selection of antigens made more immunogenic by the combination with vaccine adjuvants^5^. The field of adjuvanted subunit TB vaccine development has been energized by the ~50% efficacy achieved with the M72/AS01E candidate in Phase 2b clinical testing^6^. Other adjuvant-containing subunit TB vaccine candidates are also in clinical development^7^. For instance, ID93 + GLA-SE demonstrated promising safety and immunogenicity in Phase 2a clinical testing^8^. Despite such progress, no thermostable adjuvanted subunit TB vaccine candidates are in clinical development. Considering the enormous worldwide burden of TB, particularly in Southeast Asia and Sub-Saharan Africa, a thermostable vaccine could provide substantial advantages for global vaccine distribution^9^.

Various technologies can be employed to increase the thermostability of vaccines^10,11^. However, adapting thermostable technologies for vaccines containing adjuvants adds substantial complexity^11^. For instance, drying technologies such as lyophilization or spray-drying are often employed to enhance vaccine stability. However, maintaining the particulate structure inherent to the activity of many vaccine adjuvant formulations, including emulsions and aluminum salts, requires unique process development and characterization approaches. We previously described the formulation design, process development, and manufacture of a lyophilized formulation of the emulsion-adjuvanted subunit TB vaccine candidate ID93 + GLA-SE that maintained stability for 3 months when stored at 37 °C^12,13^.

Here, we present results from a Phase 1, randomized, double-blind clinical trial designed to evaluate the safety, tolerability, and immunogenicity of this single-vial lyophilized ID93 + GLA-SE vaccine candidate compared to the previously developed two-vial presentation in healthy adult subjects. We show the thermostable single-vial presentation of ID93 + GLA-SE elicits a similar safety profile and a comparable or improved immunogenicity profile to the non-thermostable two-vial vaccine presentation.

## Results

### Participants

**Fig. 1:**
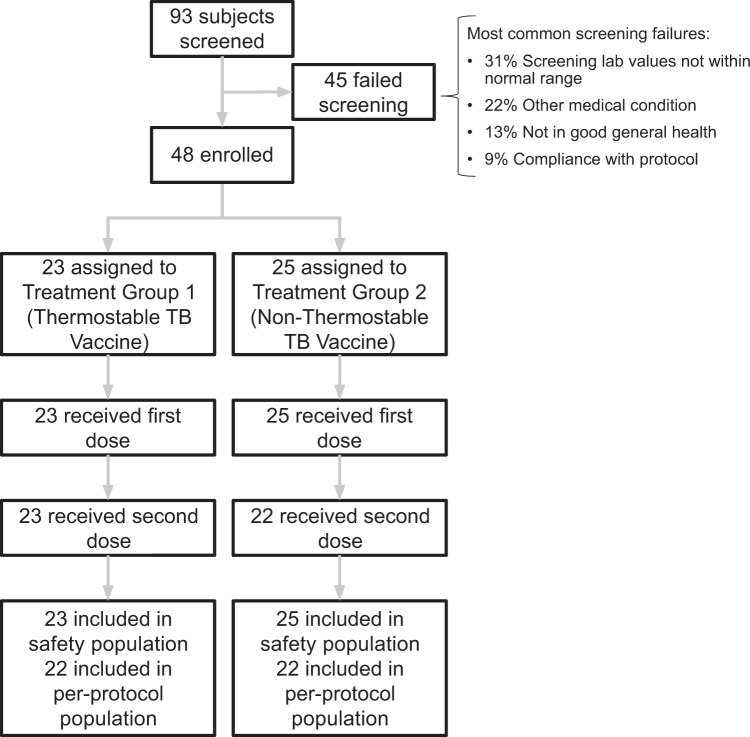
Screening, enrollment, randomization, and follow-up of study participants. Of 93 subjects screened, 48 met the study criteria and were enrolled and randomly assigned to two treatment groups. Treatment Group 1 comprised 23 participants receiving the thermostable single-vial ID93 + GLA-SE vaccine; Treatment Group 2 comprised 25 participants receiving the non-thermostable two-vial ID93 + GLA-SE vaccine. The safety population represents participants receiving at least one injection. The per-protocol population represents participants that completed the study according to plan.

Participation from human subjects in the study occurred beginning with recruitment from October 29, 2018, to follow-up, ending June 15, 2020. A total of 93 participants were screened for study inclusion over a 6-month recruitment period, among whom 48 met study entry criteria and were randomized 1:1 into two treatment groups (Fig. 1). Among the 45 screen failures, the most common reasons for exclusion were screening lab values not within normal range, other medical conditions, lack of good general health, and non-compliance with the protocol (Fig. 1). All 48 enrolled participants received at least one injection (safety population), 45 participants received both injections, and 44 participants completed the study according to plan (per-protocol population). Among the three participants that only received the first study injection in Treatment Group 2 (non-thermostable TB vaccine), two were lost to follow-up and one became ineligible due to a workplace requirement for immunization with another vaccine. Samples from participants receiving only one study injection were included in the immunogenicity data analysis prior to Study Day 56, after which only samples from participants receiving both study injections were included in the immunogenicity analysis. An additional study participant voluntarily withdrew from the study after Study Day 70. A total of 45 participants completed the long-term follow-up visit on Study Day 421 (44 received both injections and 1 received one injection). Other protocol deviations, such as missed visits and/or procedures, are described in the Supplementary Information. Table 1 summarizes the demographic and other baseline characteristics of the participants included in the safety population (*n* = 48). Thirty-six of the 48 participants (75%) were female, and 12 (25%) were male. Participants' ages at enrollment ranged from 18 to 48 years, with a median of 23 years. Participants’ mean body mass index (BMI) was 24.1. Races included 43 (90%) White, 2 (4%) Asian, 2 (4%) mixed, and 1 (2%) Black or African American.

**Table 1:** Summary of demographic data and other baseline characteristics in the safety population

	Treatment group 1: thermostable single-vial presentation of ID93 + GLA-SE	Treatment group 2: non-thermostable two-vial presentation of ID93 + GLA-SE
Age (yrs)		
Mean	28.3	27.3
Std deviation	9.07	8.82
Std error	1.89	1.76
Median	23.0	23.0
Interquartile range (IQR)	21.0–37.5	20.0–32.0
Gender		
Female	18 (78.3%)	18 (72.0%)
Male	5 (21.7%)	7 (28.0%)
Race		
White	20 (87.0%)	23 (92.0%)
Asian	2 (8.7%)	0
Black or African American	0	1 (4.0%)
Native Hawaiian or other Pacific Islander	0	0
American Indian or Alaskan native	0	0
Mixed race	1 (4.3%)	1 (4.0%)
Other	0	0
BMI (kg m^−2^)		
Mean	24.1	24.1
Std deviation	3.88	3.03
Std error	0.81	0.61
Median	24.0	23.8
IQR	20.7–26.3	21.9–25.6

### Safety

Both presentations of the ID93 + GLA-SE vaccine were determined to be safe and well tolerated in healthy adult participants following intramuscular (IM) administration. The pattern, type, severity, and frequency of adverse events (AEs) were similar across both treatment groups. Table 2 summarizes the proportion of participants with AEs by severity, relatedness to study injection, and treatment group. AEs were reported in 98% of all study participants. The highest grades reported for AEs were Grade 1 in 96% and 92% or Grade 2 in 22 and 36% of participants in Treatment Groups 1 and 2, respectively. No Grade 3 or 4 AEs, serious adverse events (SAEs), or potential immune-mediated medical conditions (PIMMCs) were reported. Local and systemic reactogenicity was common. Most participants reported injection site pain and/or tenderness, with fatigue, headache, and myalgia as the most common systemic effects. Upper respiratory tract infections or decreases in hemoglobin in some participants were not considered related to treatment (Supplementary Information, Study Protocol). Most AEs resolved without treatment, and no participants withdrew because of an AE. Therefore, the single-vial thermostable presentation of the ID93 + GLA-SE vaccine candidate was determined to be similarly safe and well tolerated compared to the two-vial non-thermostable vaccine presentation at the dose administered in healthy adults.

**Table 2:** Subjects with adverse events occurring in >5% of the safety population

	Treatment group 1: thermostable single-vial presentation of ID93 + GLA-SE (*n* = 23)	Treatment group 2: non-thermostable two-vial presentation of ID93 + GLA-SE (*n* = 25)
Any adverse event	23 (100%)	24 (96.0%)
Grade 1	22 (95.7%)	23 (92.0%)
Grade 2	5 (21.7%)	9 (36.0%)
Grade 3	0	0
Grade 4	0	0
Serious adverse event	0	0
Post-first immunization (Study Days 0–55)	19 (82.6%)	24 (96.0%)
Post-second immunization (Study Days 56–84)	19 (82.6%)	17 (68.0%)
Any local reaction	22 (95.7%)	21 (84.0%)
Injection site erythema	2 (8.7%)	0
Injection site induration	3 (13.0%)	0
Injection site pain	22 (95.7%)	21 (84.0%)
Any systemic reaction	9 (39.1%)	12 (48.0%)
Arthralgia	3 (13.0%)	1 (4.0%)
Fatigue	4 (17.4%)	10 (40.0%)
Headache	4 (17.4%)	5 (20.0%)
Myalgia	3 (13.0%)	5 (20.0%)

### Immunogenicity

**Fig. 2:**
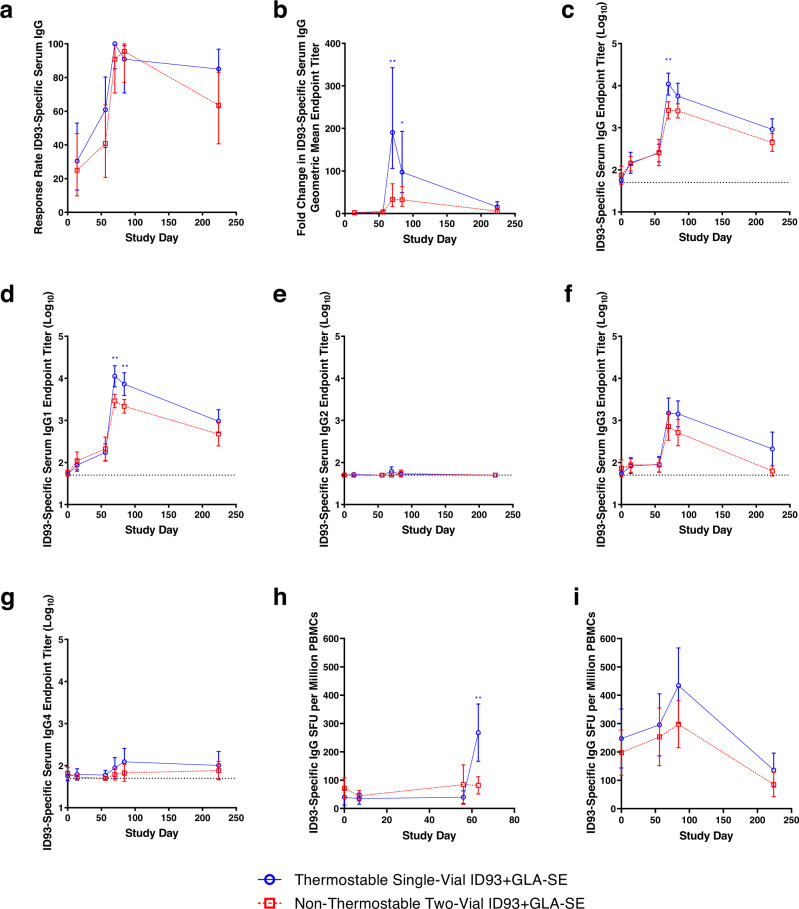
ID93-specific antibody responses in serum and B-cell responses in PBMCs. *n* = 20–25 biologically independent samples depending on treatment group and time point, see Supplementary Table 1. **a** Serum antibody response rate was calculated as at least a fourfold increase in ID93-specific IgG from baseline as measured by ELISA. Response rates were compared using Fisher’s exact test. Differences were detected based on a two-sided test and *p* ≤ 0.05 (α = 0.05) with Bonferroni adjustment to account for the multiple time points. **b**–**i** Data were compared between the two treatment groups using the Wilcoxon rank-sum test with Bonferroni adjustment to account for the multiple time points. **b** Fold changes in ID93-specific total IgG in serum from baseline as measured by ELISA. **p = 0.0023 (Study Day 70), *p = 0.0495 (Study Day 84). **c** Longitudinal plot of group MEPT for ID93-specific total IgG in serum as measured by ELISA. **p = 0.0061. **d**–**g** Longitudinal plots of group MEPT ID93-specific IgG subclasses in serum as measured by ELISA. **d** IgG1. **p = 0.0032 (Study Day 70), **p = 0.0040 (Study Day 84). **e** IgG2. **f** IgG3. **g** IgG4. **h** Enumeration of ID93-specific IgG-secreting cells in PBMC samples as measured by ELISpot assay. **p = 0.0085. **i** Enumeration of ID93-specific IgG memory B cells in PBMC samples as measured by ELISpot assay. Mean values are represented by symbols, and error bars show the 95% CI on the mean. Asterisks indicate a statistically significant difference (*p < 0.05, **p < 0.01) between treatment groups at that time point after correction for multiple comparisons. Source data are provided as a Source Data file.

IgG antibody responses to ID93 were evaluated in each treatment group. Serum IgG antibody enzyme-linked immunosorbent assays (ELISAs) were performed on samples collected on Study Days 0, 14, 56, 70, 84, and 224 for total IgG, IgG1, IgG2, IgG3, and IgG4. Total IgG was also assessed for the subunit proteins comprising ID93 (Rv1813, Rv2608, Rv3619, and Rv3620). Serum levels of ID93-specific IgG antibodies are presented as mean endpoint titer (MEPT), along with response rate and fold-change for total IgG, by treatment regimen (Fig. 2a–g and Supplementary Table 1). Participants in both ID93 + GLA-SE treatment groups developed robust ID93-specific antibody responses with low-level background detected at baseline. ID93-specific IgG responses remained elevated through 6 months after the last vaccination in both regimens. ID93-specific IgG1 and IgG3 responses were higher than IgG2 and IgG4 responses. ID93 subunit-specific total IgG responses were greatest for Rv1813, followed by Rv2608, Rv3620, and Rv3619 (Supplementary Fig. 1). In all cases, the peak antibody responses were observed after the second injection. Response rates for ID93-specific total IgG, based on a fourfold increase from baseline, were 90–100% at Study Days 70 and 84 for both treatment groups (Fig. 2a). At Study Day 224, IgG response rates ranged from 63–85% (Fig. 2a). Comparison of the thermostable single-vial presentation to the non-thermostable two-vial presentation showed that the thermostable format elicited a significantly higher antibody response in multiple readouts at the peak time points. For instance, compared to the non-thermostable two-vial presentation, the thermostable single-vial formulation enhanced the fold-change in ID93-specific IgG (190.5 vs 33.0 at Study Day 70 [*p* = 0.0023] and 97.4 vs 32.2 at Study Day 84 [*p* = 0.0495]) (Fig. 2b) and the ID93-specific IgG MEPT (10868 vs. 2583 at Study Day 70 [*p* = 0.0061]) (Fig. 2c).

ID93-specific IgG antibody-secreting cells in PBMC samples were assessed by enzyme-linked immunospot (ELISpot) on Study Days 0, 7, 56, and 63. Although responses remained near baseline levels for most time points in both treatment groups, a remarkable increase in the number of ID93-specific IgG antibody-secreting cells in the thermostable single-vial vaccine group was measured at Study Day 63, while the response in the non-thermostable two-vial vaccine group remained low (Fig. 2h). ID93-specific IgG memory B cells in peripheral blood mononuclear cell (PBMC) samples were also measured by ELISpot on Study Days 0, 56, 84, and 224. The IgG memory B cells appeared to increase in both treatment groups 4 weeks following the second injection, but responses had returned to baseline levels by Study Day 224 (Fig. 2i). Thus, consistent with the pattern evident in the serum IgG responses described earlier, the thermostable single-vial vaccine presentation significantly increased ID93-specific IgG-secreting plasma cells compared to the non-thermostable two-vial presentation following the second immunization.

Mucosal ID93-specific IgA responses were assessed by ELISA using nasal swabs and tear samples at Study Days 0, 70, and 224. However, ID93-specific IgA response in the mucosal samples remained at baseline levels at all time points in both treatment groups (Supplementary Fig. 2a, b). Likewise, ID93-specific IgA antibody-secreting cells and IgA memory B cells in PBMC samples did not significantly increase above baseline levels (Supplementary Fig. 2c, d). Thus, neither treatment group appeared to elicit antigen-specific IgA antibody responses in the mucosal samples or in PBMCs.

**Fig. 3:**
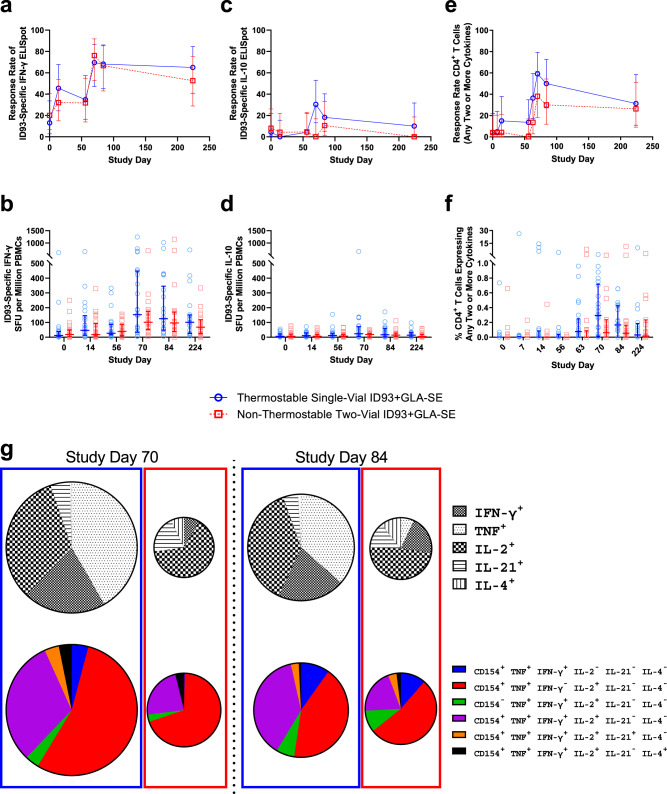
ID93-specific T cell responses in PBMCs. *n* = 16–25 biologically independent samples depending on treatment group and time point, see Supplementary Tables 2–4. **a** IFN-γ response rate by ELISpot assay using ID93-stimulated PBMCs and calculated by MIMOSA as a change from baseline using background-subtracted SFUs. **b** Enumeration of ID93-stimulated IFN-γ-secreting cells in PBMC samples as measured by ELISpot assay. **c** IL-10 response rate by ELISpot assay using ID93-stimulated PBMCs and calculated by MIMOSA as a change from baseline using background-subtracted SFUs. **d** Enumeration of ID93-stimulated IL-10-secreting cells in PBMC samples as measured by ELISpot assay. **e** Response rate by ICS of CD4^+^ T cells expressing Any Two cytokines among CD154, TNF, IFN-γ, IL-2, IL-21, and IL-4 calculated by MIMOSA as a change from baseline using background-subtracted frequencies and total counts. **f** Frequencies of ID93-stimulated CD4^+^ T cells expressing Any Two cytokines in PBMC samples as measured by ICS. **g** Single cytokine and polyfunctional cytokine CD4^+^ T cell phenotype at Study Days 70 and 84 as measured by ICS. Proportions of ID93-stimulated CD4^+^ T cells expressing different single cytokines and cytokine combinations at Study Days 70 and 84 represented by median frequencies. The pie chart area is proportional to the total median frequencies for each readout. **a**, **c**, **e** Response rate plots show mean values represented by symbols, and error bars show the 95% CI on the mean. Response rates were compared using Fisher’s exact test. Differences were detected based on a two-sided test and *p* ≤ 0.05 (α = 0.05) with Bonferroni adjustments to account for the multiple time points. **b**, **d**, **f** Dot plots represent all background-subtracted values as symbols, with solid lines representing median values and error bars representing the interquartile range. Data were compared between the two treatment groups using the Wilcoxon rank-sum test with Bonferroni adjustment to account for the multiple time points. Source data are provided as a Source Data file.

To quantify the number of IFN-γ and IL-10 cytokine-secreting cells in PBMC samples in response to ID93, ELISpot assays were performed on samples collected on Study Days 0, 14, 56, 70, 84, and 224. IFN-γ response rates, based on MIMOSA analysis, peaked after the second injection at 70–76% at Study Day 70 for both treatment groups (Fig. 3a). At Study Day 224, IFN-γ response rates ranged from 53 to 65% (Fig. 3a). Participants in both ID93 + GLA-SE treatment groups developed robust ID93-specific IFN-γ PBMC recall responses as measured by total SFU per million PBMCs producing IFN-γ (Supplementary Table 2 and Fig. 3b). These responses were sustained through 6 months after the last vaccination in both regimens. On the other hand, low reactivity to ID93 was seen in IL-10-producing cells (Supplementary Table 3 and Fig. 3c, d). This pattern of increased numbers of IFN-γ excreting cells and low levels of IL-10 excreting cells is indicative of a Th1-type immune response. A comparison of the two vaccine presentations showed no significant differences, although the thermostable single-vial vaccine format tended to elicit greater magnitude responses. IFN-γ and IL-10 levels from PBMCs stimulated by ID93 subunit peptide pools followed similar patterns as described above for ID93-stimulated PBMCs but at lower magnitudes (Supplementary Fig. 3).

The percentage of CD4^+^ and CD8^+^ T cells producing two or more cytokines in response to ID93 was measured by intracellular cytokine staining (ICS) with flow cytometry using PBMC samples collected on Study Days 0, 7, 14, 56, 63, 70, 84, and 224. The response rate and frequencies of antigen-specific CD4^+^ T cells expressing Any Two or more cytokines (i.e., cells expressing two or more immune markers among IFN-γ, TNF, IL-2, IL-4, IL-21, and CD154) were calculated using background-subtracted values. ID93-specific CD4^+^ T cell responses were observed with both vaccine presentations (Supplementary Table 4 and Fig. 3e, f). Peak response rates (analyzed by MIMOSA) occurred at Study Day 70 in both treatment groups (38–59%) (Fig. 3e). Response rates persisted at 26–31% at Study Day 224, 6 months after the last study injection (Fig. 3e). Analysis of single and combination cytokine profiles of ID93-specific T cells indicated that the highest frequencies of expression were attributable to IFN-γ, TNF, and/or IL-2, with low IL-4 response (Fig. 3g). While the thermostable vaccine formulation tended to elicit a higher median frequency of TNF response, both vaccine presentations induced a polyfunctional and Th1-skewed immune response. However, minimal impacts were observed from either treatment on ID93-specific response frequencies from CD8^+^ T cells (Supplementary Fig. 4d and Supplementary Table 5). In summary, while differences in individual readouts were not statistically significant, the thermostable vaccine presentation exhibited an overall trend of greater CD4^+^ T cell cytokine response frequencies compared to the non-thermostable vaccine presentation.

The frequency of other T cell subtypes, including T follicular helper (Tfh) cells, T effector memory cells, and T central memory cells, as well as other immune cells, including natural killer (NK) cells and NK T cells, was measured by ICS with flow cytometry in PBMC samples. Both treatments enhanced CD4^+^ T central memory cell frequencies but not effector memory cell frequencies compared to baseline (Supplementary Fig. 4a, b). The thermostable single-vial presentation also elicited a modest increase in Tfh cell frequencies at Study Day 63 compared to baseline (Supplementary Fig. 4c). However, minimal effects were observed from either treatment on ID93-specific response frequencies from NK cells or NK T cells (Supplementary Fig. 4e, f).

Finally, net intracellular mycobacterial growth inhibition from each treatment was measured using whole blood samples collected on Study Days 0, 70, and 224. However, neither vaccine presentation improved mycobacterial growth inhibition as indicated by time to positivity (Supplementary Figure 5).

## Discussion

To our knowledge, this study represents the first thermostable subunit tuberculosis vaccine candidate to be evaluated in clinical testing^11,14^. For other disease indications, three lyophilized adjuvanted subunit vaccine candidates have entered clinical testing, but clinical results have only been published for one candidate without evaluation of its potential thermostability characteristics^15–18^. Thus, the present report represents a major step forward for the field of thermostable lyophilized adjuvant-containing vaccine candidates. Notably, some liquid vaccine formulations with thermostable properties have progressed through clinical testing and even to licensure, with significant beneficial impact due to their thermostability profile demonstrated in field use^11,19,20^. Nevertheless, the stability of such vaccines outside of refrigerated temperatures is generally limited to days or a few weeks, whereas the thermostable single-vial presentation of ID93 + GLA-SE is stable for 3 months at 37 °C^12,13^.

The specific objectives of the current Phase 1 trial were to evaluate the safety and immunogenicity of a thermostable single-vial presentation of the ID93 + GLA-SE vaccine candidate compared to the previously developed non-thermostable two-vial presentation. The safety profiles demonstrated here for both vaccine presentations were consistent with the generally mild reactogenicity evident in previous clinical trials of the two-vial vaccine presentation^8,21,22^. Injection site pain was the most common AE, and no Grade 3 or 4 AEs or SAEs were identified for either vaccine presentation. Thus, the thermostable vaccine presentation maintained the excellent safety profile of the non-thermostable ID93 + GLA-SE vaccine candidate.

A Th1-type T cell response is considered favorable for protection against *Mycobacterium tuberculosis* (*M. tuberculosis*)^23^. The non-thermostable two-vial ID93 + GLA-SE presentation was previously shown to induce robust antigen-specific IgG and Th1-type CD4^+^ T cell responses^8,21,22^. The immunogenicity results reported here for both vaccine presentations were consistent with this profile, including polyfunctional CD4^+^ T cells expressing IFN-γ, TNF, and IL-2. However, multiple readouts employed in the current trial had not been included in previous clinical evaluations of ID93 + GLA-SE, including the mucosal secretion antibody ELISAs, the *M. tuberculosis* growth inhibition assay on whole blood, and the ELISpot enumeration of T cells and B cells. While minimal impact was apparent from either of the vaccine presentations on mucosal antibody responses or *M. tuberculosis* growth inhibition in whole blood, ELISpot-based enumeration of cytokine-producing cells in ID93-stimulated PBMC samples indicated that both presentations of ID93 + GLA-SE elicited a robust IFN-γ response and low IL-10 response, which is consistent with the Th1-type response quality evident in the ICS data from the current and previous trials. Interestingly, an overall trend for higher magnitude peak T cell responses was observed in the thermostable single-vial treatment group compared to the non-thermostable treatment group.

While B cell and antibody responses are not sufficient for protective efficacy against TB, their multiple complementary roles to T cell-mediated immunity are increasingly appreciated, and more clinical investigation is warranted in this regard^24–26^. Remarkably, the thermostable single-vial vaccine presentation elicited significantly more antigen-specific IgG and IgG1 in serum collected 2–4 weeks following the second immunization than the non-thermostable two-vial presentation. Moreover, the enumeration of ID93-specific IgG-secreting cells in PBMC samples revealed a robust response 1 week following the second immunization for the thermostable single-vial vaccine presentation but not the non-thermostable two-vial presentation. It is not clear how to specifically attribute the unexpected differences in the magnitude of immune responses between treatment groups. A higher baseline MEPT could impact post-vaccination fold-change calculations, but this does not explain the differences in absolute MEPT values. While each treatment group received the same dose of antigen and adjuvant, other factors, including the excipient composition, date of manufacture, and manufacturing processes, necessarily differed.

The multiple immunological assays, sample types, and time points employed in this study represent the most comprehensive clinical immunogenicity evaluation of the ID93 + GLA-SE vaccine candidate in healthy adults to date. Nevertheless, the study had several limitations, including the lack of placebo treatment groups, but these were deemed unnecessary due to previous clinical evaluation of ID93 + GLA-SE compared to ID93 alone or saline placebo^8,22^. Other limitations include that no established immune correlates of protection against TB exist and that the participants were not BCG-vaccinated or pre-exposed to *M. tuberculosis*. Thus, it is not possible to predict how the immunogenicity profile of ID93 + GLA-SE, and particularly the enhanced responses elicited by the thermostable vaccine formulation, would translate to impacts on protective efficacy. Finally, this study also enrolled primarily white women, which makes extrapolations to other populations with a high burden of TB disease challenging.

The impact of the thermostable single-vial formulation of ID93 + GLA-SE on vaccine manufacturing scalability and cost is another important consideration. We estimate that the excipient cost in the thermostable presentation would be approximately $0.15 more per dose than the non-thermostable composition at commercial scale. Furthermore, additional costs would be associated with the multi-day lyophilization processing time. However, these increased costs could be mitigated by the anticipated cost savings and reduced wastage associated with the less stringent storage requirements of the thermostable formulation compared to the non-thermostable presentation. Furthermore, lyophilization is already a well-established pharmaceutical processing technique that is employed in the production of many licensed vaccines.

In summary, this Phase 1 clinical evaluation indicated that the thermostable single-vial presentation of ID93 + GLA-SE elicits a similar safety profile and a comparable or improved immunogenicity profile to the non-thermostable two-vial vaccine presentation. An effective thermostable TB vaccine would have major implications for global health impact. Furthermore, this study demonstrates proof-of-concept that an adjuvant-containing vaccine can be formulated in a single-vial thermostable presentation without detrimentally impacting clinical immunogenicity or safety characteristics.

## Methods

### Study design and participants

This study was a Phase 1, randomized, double-blind clinical trial designed to evaluate the safety, tolerability, and immunogenicity of thermostable single-vial lyophilized ID93 + GLA-SE compared to the non-thermostable two-vial presentation consisting of lyophilized ID93 and liquid GLA-SE administered as two IM injections in healthy adult participants. The study was performed under an investigational new drug (IND) with the US Food and Drug Administration (FDA), and the protocol, informed consent form, and other study materials were approved by the Saint Louis University Institutional Review Board. The clinical trial is registered at ClinicalTrials.gov under Identifier NCT03722472.

The study was conducted at a single site (Saint Louis University Center for Vaccine Development in St. Louis, MO) and recruited participants from St. Louis, Missouri, and the surrounding area. A total of 48 male and female participants aged 18 to 55 years and in general good health were enrolled, as planned per Study Protocol (Supplementary Information). Participants were counseled on the study and underwent the informed consent process. The screening included an evaluation of medical history, medication history, a physical examination, safety laboratory tests, and urinalysis. The complete inclusion and exclusion criteria are shown in Supplementary Information (Study Protocol). Eligibility criteria included healthy males and females 18–55 years of age with negative human immunodeficiency virus (HIV), hepatitis B surface antigen (HBsAg), and hepatitis C virus (HCV), and no history of BCG vaccination. Participants who met all eligibility criteria were randomized 1:1 into two treatment groups. Study injections were performed on Study Days 0 and 56. General safety was evaluated on Days 0, 7, 56, 63, and 84 for each participant. Blood was assessed for safety laboratory analyses (hematology and serum chemistry) on the day of screening and on Study Days 7 and 63. All participants completed a written participant memory aid that solicited local and systemic reactogenicity AEs for 7 days following each study injection. Unsolicited AEs were recorded through Study Day 84 (28 days following the last study injection). The occurrence of SAEs and the onset of any PIMMCs were recorded throughout the study period (approximately 421 days). Immunogenicity was assessed on Study Day 0 (pre-dose) and on Study Days 7, 14, 56, 63, 70, 84, and 224. Participants were compensated $75 per study visit and $10 per telephone call visit.

### Sample size and study endpoints

The sample size of 48 participants was determined by what is reasonable for a Phase 1 trial and allows only preliminary safety and immunogenicity information relevant to the progression to larger trials. Based on previous clinical experience with the non-thermostable presentation of ID93 + GLA-SE, we estimated that a sample size of 24 participants per group would allow the detection of an increase in systemic adverse reaction frequency of >32.5% in the thermostable presentation group compared to the non-thermostable presentation group at 80% power with a one-sided confidence interval of 95%, and detection of a decrease in serum antibody response rate of >10% in the thermostable presentation group compared to the non-thermostable presentation group at 90% power with a one-sided confidence interval of 95%. This study was inclusive of all healthy adults who met the inclusion/exclusion criteria, regardless of sex. Sex was determined based on self-reporting. No sex- or gender-based analyses were performed as the study was not designed with sufficient power to adequately address this aspect. The primary endpoints included (1) the number of participants experiencing solicited local injection site reactions within 7 days following each study injection, (2) the number of participants experiencing solicited systemic reactions within 7 days following each study injection, (3) the number of participants spontaneously reporting AEs from Study Day 0 through Study Day 84, and (4) number of SAEs considered related to any of the study injections reported at any point during the study period. The secondary endpoints included (1) proportion of participants with at least a 4-fold increase in IgG antibody responses to ID93 on Study Days 14, 56, 70, 84, and 224 relative to baseline (Study Day 0) as assayed by ELISA; (2) mean fold-change from baseline in IgG antibody responses to ID93 on Study Days 14, 56, 70, 84, and 224 relative to baseline (Study Day 0) as assayed by ELISA; (3) the number of IFN-γ and IL-10 cytokine-secreting cells in PBMC samples in response to ID93 at Study Days 14, 56, 70, 84, and 224 relative to baseline (Study Day 0) as assayed by ELISpot; and (4) the percentage of CD4^+^ and CD8^+^ T cells producing two or more cytokines in response to ID93 as measured by ICS with flow cytometry of PBMCs on Study Days 0, 7, 14, 56, 63, 70, 84, and 224. Exploratory endpoints included (1) IgG subclass antibody responses to ID93 at Study Days 0, 14, 56, 70, 84, and 224; (2) net intracellular growth inhibition of *M. tuberculosis* using whole blood on Study Days 0, 70, and 224; (3) IgA antibody responses to ID93 in mucosal secretions (nasal swabs and tear collections) as measured by ELISA on Study Days 0, 70, and 84; (4) number of antibody-secreting cells in PBMCs as assayed by short-culture B cell ELISpot on Study Days 0, 7, 56, and 63; (5) the number of antigen-specific memory B cells in PBMCs as assayed by long-culture B cell ELISpot on Study Days 0, 56, 84, and 224; and (6) the percentage of Tfh cells and T cell homing markers in PBMCs as measured by immunophenotyping flow cytometry on Study Days 0, 7, 14, 56, 63, 70, 84, and 224.

### Randomization and blinding

Participants were enrolled by randomization into two treatment groups according to the following steps. A master log of all screened participants was maintained. At screening, participants who signed the informed consent form and met all inclusion and none of the exclusion criteria were identified as eligible to be enrolled in the study. Eligible participants were assigned a sequential identification number when they presented to the clinic on Study Day 0. Statisticians at DF/Net Research (Seattle, WA) generated the list of randomized treatment assignments and included it in the enrollment module of the Interactive Web Randomization System (IWRS), which is integrated into the DFexplore software. The study coordinator or designee at the clinical site performed the enrollment and randomization using DFexplore. Block randomization of appropriate size was used to balance enrollment in a 1:1 ratio into each of the two groups. A designated individual at the study site was provided with a treatment key, which linked the treatment code to the actual treatment assignment and was kept in a secure place.

The study was conducted as a double-blind trial. Participants, investigators, study personnel performing any study-related assessments following study injection, and laboratory personnel performing immunology assays were blinded to treatment assignment. The randomization scheme from DF/Net Research was provided to unblinded study personnel (i.e., site pharmacists performing study product preparations and unblinded study product administrators).

### Vaccination

ID93 is a recombinant subunit antigen, and GLA-SE is a squalene emulsion containing the synthetic Toll-like receptor 4 agonist glucopyranosyl lipid A (GLA)^8,27^. This was the fifth clinical trial in which the ID93 + GLA-SE vaccine was administered to human participants but the first for the thermostable single-vial presentation (NCT01599897, NCT01927159, NCT02465216, NCT02508376, and NCT03722472). Results from a previous trial informed dose selection (2 µg ID93 and 5 µg GLA-SE) and schedule for this trial^8^. The thermostable single-vial lyophilized presentation of ID93 + GLA-SE was developed and manufactured as described earlier, including optimization of excipient content, lyophilization process engineering, and comprehensive physicochemical and potency stability monitoring^12,13^. The thermostable single-vial lyophilized ID93 + GLA-SE was reconstituted with sterile water for injection (WFI) prior to administration. The non-thermostable two-vial presentation was prepared by reconstituting lyophilized ID93 protein with sterile WFI and mixing at 1:1 v:v with liquid GLA-SE prior to administration. For the two-vial presentation, ID93 was manufactured by the University of Iowa Center for Biocatalysis and Bioprocessing (CBB, Coralville, IA), and lyophilization was performed by the University of Iowa Pharmaceuticals (Iowa City, IA). Liquid GLA-SE was manufactured by the Infectious Disease Research Institute (IDRI; Seattle, WA), now the Access to Advanced Health Institute (AAHI). For the single-vial presentation, bulk drug substances ID93 and GLA-SE were manufactured at CBB and IDRI, respectively, and the final drug product was manufactured at Lyophilization Technology, Inc (Warminster, PA). All investigational products were provided to the Investigator (Daniel F. Hoft at Saint Louis University) by the Sponsor (IDRI). Vials of ID93 + GLA-SE, ID93, and GLA-SE were shipped and stored at the study site at 2–8 °C; the temperature during shipping and storage was continuously monitored. Sterile WFI was provided by the study site.

Study vaccine preparation, including vaccine dilutions and admixing for the two dosing groups, was performed by the unblinded site pharmacist using an aseptic technique on the same day of study vaccine administration but no more than 2 h prior to administration. Once reconstituted, the vaccine from both treatment groups (single- and two-vial presentations) looked identical. The designee assigned to administer the study injections was supplied with a loaded syringe labeled with the participant identification number and hospital-required identification data. The time of preparation and time of administration was noted in the source documentation. All study injections were 0.5 mL in volume and administered intramuscularly in the deltoid of the non-dominant arm.

### Sample collection and handling

All samples were collected at the study site, logged, and tracked (Saint Louis University Center for Vaccine Development in St. Louis, MO). Safety samples were shipped the same day as a collection to Quest Diagnostics, formerly LabOne (Lenexa, KS), for processing. Immunology samples for secondary and exploratory endpoints were processed at Saint Louis University. Serum and PBMC samples from each participant were stored here until completion of the study, at which point samples were shipped to Advanced Bioscience Laboratories (ABL Inc.; Rockville, MD) for assays and analysis in two separate shipments to mitigate the risk of sample loss due to temperature excursions. PBMCs were shipped using liquid nitrogen-charged dry shippers, and serum samples were shipped on dry ice.

For the Mycobacteria Growth Indicator Tube (MGIT) assay, blood was collected using sterile sodium heparin collection tubes. For safety hematology tests, blood was collected in tubes with EDTA. To obtain serum samples for safety chemistry and antibody analysis, blood was collected using serum separator tubes (SST). For antibody analysis, following centrifugation at 750–930 × *g* for 10–15 min at ambient temperature, the supernatant was collected. Serum samples at ~500 µL each were divided (into two primary and two backup vials), placed into pre-chilled 9 × 9 cryoboxes, immediately frozen at −70 °C ± 10 °C, and stored in an upright position at −70 °C ± 10 °C until shipment to ABL.

To obtain PBMC samples, blood was collected into 8-mL CPT tubes (BD Biosciences, Franklin Lakes, NJ). Following centrifugation at 1800 × *g* for 30 min at ambient temperature in a horizontal rotor swing-out head centrifuge, plasma was aspirated, and the cell layer was collected from the tube with a pipette and transferred to a conical centrifuge tube. Cells were then washed and resuspended in phosphate-buffered saline (PBS). PBMC samples were subsequently aliquoted at 8 × 10^6^ in 1 mL of freezing media (fetal bovine serum (FBS) containing 10% dimethylsulfoxide (DMSO)) per cryovial. PBMCs were frozen immediately in CoolCell (Corning, Corning, NY) or Mr. Frosty (Thermo Fisher Scientific, Waltham, MA) containers at −70 °C ± 10 °C. Each participant’s PBMC vials were distributed into two separate (primary and backup) pre-chilled 9 × 9 cryoboxes in an upright position in a freezer, then moved to a liquid nitrogen or vapor phase cryogenic freezer within 1 week, and stored there until shipment to ABL.

For nasal sample collection, the same sterile PurFlock swab (Puritan Medical Products, Guilford, ME) was inserted into each nasal cavity alongside the nasal septum and rotated 360° in each direction waiting for 5 s after each rotation before carefully removing the swab. After sampling both nostrils, the swab tip was placed in 3 mL of buffer containing Dulbecco’s PBS (DPBS) with 5 mM l-glutamic acid and 10% sucrose (pH 7.2) in a 15-mL centrifuge tube. The swab applicator stick was removed, leaving the swab in the tube. Nasal swab samples were held on wet ice or in a 2–8 °C refrigerator for up to 2 h until freezing. Samples were frozen at −70 °C ± 10 °C and stored at this temperature for at least 24 h but not more than 1 month until processing. Sample processing consisted of vortexing the tube for 1 min and then pressing the swab against the side of the tube before removing. The remaining sample was then aliquoted into cryotubes and stored at −70 °C ± 10 °C until analysis at Saint Louis University.

Tear samples were collected by first squeezing an orange rind directly over the participant’s eye. The participant then blinked several times until tears were generated. The tears (target volume 125 µL) were collected using a capillary tube which drained into a screw-cap collection microtube. The affected eye was then irrigated with an eye wash solution and a commercial eye drop solution. Tear samples were held on wet ice for up to 2 h before processing by centrifugation at 20,000 × *g* for 5 min at ~4 °C. The supernatant was collected in 15-µL aliquots, placed into 0.5-mL storage vials, and stored at −70 °C ± 10 °C until analysis at Saint Louis University.

### Safety monitoring

The occurrence of all AEs was recorded through Study Day 84, whereas SAEs and PIMMCs were monitored through Study Day 421. AEs were graded by severity (i.e., Grades 1, 2, or 3) and by relationship to the study injection (i.e., not, possibly, probably, or definitely related) according to definitions provided in the Study Protocol (Supplementary Information). For reporting purposes, consistent with the National Institute of Allergy and Infectious Diseases (NIAID) guidelines, definitely, probably, and possibly related AEs were considered related to study injection. Not related AEs were considered unrelated. An SAE was defined as any AE that resulted in death, was considered life-threatening, required hospitalization or prolongation of hospitalization, resulted in persistent or significant disability/incapacity, or resulted in a congenital anomaly/birth defect in the offspring of a study participant.

Local injection site reactions were assessed on the day of the study injection (prior to injection and 60 min after injection) and for 7 days after the study injection by grading pain, erythema, and induration. Solicited systemic reactions were defined as the occurrence of headache, arthralgia, chills, loss of appetite, fever, fatigue, and/or myalgia in the 7 days following each study injection. These symptoms were characterized as Grade 1 (mild), Grade 2 (moderate), or Grade 3 (severe). Memory aid booklets were distributed to each participant to collect local and systemic AE information covering the 7-day period following each study injection. All participants were instructed to record oral temperature and local and general signs and symptoms on individual memory aids on the evening of the day of injection and at approximately the same time daily for the 6 days following study injections. Erythema and induration at the injection site were measured and graded, using the measuring tool provided. Participants were also asked to record any medications taken. The memory aid was brought to the clinic on the scheduled visit 7 days after each injection for review and was collected by the study research staff on Study Days 7 and 63. The memory aid was a tool to help the Investigator and/or designee to engage in conversation with the participant about any AEs that may have occurred following injections.

Pulse, oral temperature, respiratory rate, and systolic and diastolic blood pressure were assessed on Study Days 0, 7, 56, 63, and 84. Safety lab tests (hematology and serum chemistry) were performed 7 days after each injection. Changes in vital signs or lab test values meeting Grade 1 or higher grade were recorded as AEs. Urine pregnancy tests were performed in the study clinic using a commercial test. Serology for HIV, HBsAg, HCV, and QuantiFERON-TB Gold was performed at screening. Unscheduled follow-up laboratory tests were done at the discretion of the study clinician. All serology, hematology, and chemistry tests were performed at Quest Diagnostics.

This clinical trial utilized a Safety Monitoring Committee (SMC) to monitor subject safety and study halting criteria. The SMC met 1 year after the start of the trial to review the current study status and discuss the safety summary report to date. No significant safety concerns were identified at that point. Therefore, the SMC recommended that the study proceed as planned with no changes.

### Immunogenicity assays

All immunogenicity assessments on serum and PBMC samples were performed by ABL. Serum ELISAs were performed using high-binding 384-well ELISA plates coated for 2 h at ambient temperature with either purified ID93 or subunit proteins (RV1813, RV2608, RV3619, or RV3620), each at 1 µg per mL. Plates were then washed three times using Wash Buffer A (Teknova, Hollister, CA) and blocked for 4 h at ambient temperature with blocking buffer (1% w/v bovine serum albumin [BSA] and 0.05% w/v polysorbate 20 in PBS). The blocking buffer was then discarded, and samples were added to each well. Control serum was diluted fourfold, beginning with an initial 1:100 dilution in 0.1% BSA with 0.05% polysorbate 20 in PBS, added to appropriate wells, and incubated overnight at 4 ^o^C. Plates were then washed seven times prior to adding horseradish peroxidase (HRP)-conjugated secondary antibodies (see Supplementary Notes for specific antibodies and dilutions) to appropriate wells in accordance with the specific ELISA being performed and incubated for 1 h at ambient temperature. Plates were then washed seven times, developed colorimetrically by adding SureBlue TMB peroxidase substrate (Kirkegaard & Perry Laboratories [KPL], Gaithersburg, MD), incubated for ~10 min at ambient temperature, and stopped using 1 N sulfuric acid. Optical densities (ODs) were quantified at 450 and 570 nm using a plate reader (Molecular Devices). Subtracted OD data (450–570 nm) was used to perform subsequent data analyses. Endpoint titers were calculated by performing a least-squares fit of the OD values at each dilution to a four-parameter least-squares sigmoidal curve. The cutoff values were calculated as the average of the negative control sera at all dilutions plus six times or three times the standard deviation depending on antibody isotype. Samples with OD values lower than the cutoff were assigned an endpoint titer of 1.699, representing the log transformation of 0.5 times the lowest dilution.

Secretory immunoglobulin A (sIgA) ELISAs were performed with a nasal swab and tear samples collected on Study Days 0, 70, and 224^28^. For antigen-specific sIgA ELISA, Immulon 2 plates were coated with 1 µg per mL of purified ID93 protein (AAHI, Seattle, WA) diluted in PBS and incubated overnight at 4 °C, 5% CO_2_. Plates were then washed with PBS plus 0.05% polysorbate 20 and blocked with 1% BSA in PBS for ~1 h at 37 °C. Following blocking, the plates were washed, nasal swab and tear samples were added to duplicate wells at optimal dilutions predetermined for each type of sample, and the plates were incubated overnight at 4 °C. Plates were then washed with PBS plus 0.05% polysorbate 20 and, with the addition of biotin-conjugated purified goat anti-human IgA (Cat# 5260-0027, KPL, Seracare Life Sciences) at 500-fold dilution, were incubated for 1.5 h at room temperature protected from light. After the incubation, the plates were washed with PBS plus 0.05% polysorbate 20, Phosphatase-labeled Streptavidin (KPL) was added, and plates were incubated for 1.5 h at room temperature, protected from light. At the end of incubation, pNPP (SIGMA*FAST* p-Nytrophenyl phosphate Tablets, MilliporeSigma) substrate solution was prepared, then plates were washed with PBS plus 0.05% polysorbate 20, and the substrate solution was added. The plates with substrate solution were incubated for 50 min (at room temperature and protected from light), AP stopping solution (KPL) was added, and absorbance was read at 405/550 dual wavelength. The total sIgA ELISA was performed following the IgA (Human) ELISA Kit (Abnova, Taipei, Taiwan; #KA2110) protocol.

ICS was performed according to the following procedures. PBMCs from study participants were thawed and rested in R10 media (RPMI 1640 with 10% FBS, 1% l-glutamine, and 1% penicillin-streptavidin) at a maximum concentration of 2 × 10^6^ cells per mL overnight at 37 °C and 5% CO_2_. PBMCs were then resuspended to a concentration of 10 × 10^6^ cells per mL and distributed so that 1 × 10^6^ cells per mL were each stimulated with 0.25 μg per mL Staphylococcal enterotoxin B (SEB) or 10 μg per mL ID93 or 0.3% DMSO, and a CD28/CD49d co-stimulation cocktail was also added to each. After PBMCs were incubated for 2 h at 37 °C and 5% CO_2_, 1X brefeldin A was added to retain secretory proteins (e.g., cytokines and interleukins) intracellularly. Activation continued for an additional 10 h at 37 °C and 5% CO_2_ with cells incubated in a sealed plastic bag containing a damp towel. Cell counts and percent viability were monitored using the Guava EasyCyte flow cytometer (Luminex, Austin, TX). Cells were then stained in multiple stages to detect region/zone-partitioned targets. Cells were stained separately: first with a 500-fold dilution of viability dye (Human CD14 BV510, Cat# 301842, BioLegend) for 20 min at ambient temperature followed by 5 µg per mL human Fc block for 10 min at ambient temperature (protected from light), and then with an extracellular surface marker cocktail for 20 min at ambient temperature. A complete list of antibodies and dilutions and other reagents used are in the Supplementary Notes. Next, cells were permeabilized and fixed with BD CytoFix/CytoPerm (BD Biosciences) for 10 min at ambient temperature (protected from light), followed by 1X Perm/Wash buffer washes. Finally, cells were stained with an intracellular marker cocktail for 30 min at ambient temperature (protected from light), fixed with 1X BD Stabilizing Fixative for 20 min at ambient temperature, and analyzed within 16 h post-staining on the BD LSRFortessa flow cytometer. Data on cell population counts and frequencies were collected using FlowJo v10.8.1 software (BD Biosciences).

A short-term B cell ELISpot assay was performed to detect the frequency of circulating antibody-secreting cells. Plates were activated with 35% alcohol, coating appropriate wells with either ID93 capture antigen or IgG/IgA capture antibody (Clone MT91/145, Cat# 3850-3-1000, or Clone MT57, Cat# 3860-3-1000, respectively, MabTech) at 100-fold dilution, and incubated overnight at 4 °C. Plates were then blocked using R10 media. PBMCs were thawed, incubated for 2 h at 37 °C and 5% CO_2_, and counted prior to seeding onto blocked wells (5 × 10^5^ cells per well for ID93 or 1 × 10^5^ cells per well for IgG/IgA). Cells were cultured for 18–22 h at 37 °C and 5% CO_2_. Plates were then washed, incubated with anti-IgG or anti-IgA detection antibodies (Clone MT78/145, Cat# 3850-6-250, or Clone MT20, Cat# 3860-6-250, respectively, MabTech) at 500-fold dilution for 2 h at ambient temperature, washed, incubated with anti-biotin streptavidin-alkaline phosphatase (AP) secondary antibody (Cat# 3310-9-1000, MabTech) for 1 h at ambient temperature, washed, and finally developed using nitro-blue tetrazolium/5-bromo-4-chloro-3’-indolyphosphate (NBT/BCIP) for 7 min at ambient temperature before stopping with distilled water. Within 2 days post-development, plates were scanned, counted, and subjected to quality control using the ImmunoSpot analyzer (Cellular Technology Limited, Cleveland, OH).

A long-term B cell ELISpot assay to detect memory B cells was conducted following the same procedures described above, except that PBMCs were first cultured for 3 days with R10 media supplemented with Human B-Poly-S polyclonal B cell resiquimod/IL-2 stimulator (Cellular Technology Limited) and then plated (2 × 10^5^ cells per well for ID93 or 5 × 10^4^ cells per well for IgG/IgA) and developed on the 4th and 5th days, respectively.

IFN-γ and IL-10 T cell ELISpot assays were conducted according to the following procedures. Plates were activated with 35% alcohol, coating plates with capture antibodies to either IFN-γ or IL-10 (Clone 1-D1K, Cat# 3420-3-250, or Clone 9D7, Cat# 3430-3-250, respectively, MabTech) at 100-fold dilution, and incubated overnight at 4 °C. Study sample PBMCs and cytomegalovirus, Epstein-Barr virus, influenza virus, and tetanus toxin (CEFT) donor control PBMCs were thawed, counted, and incubated overnight at 37 °C and 5% CO_2_. Plates were then blocked by adding R10 media and stored for 2–4 h at 37 °C and 5% CO_2_. Study samples and control PBMCs were re-counted and resuspended to appropriate concentrations. Cells were seeded to 96-well polyvinylidene fluoride (PVDF) plates according to the following densities. All study samples were seeded at 2 × 10^5^ cells per well for both T cell ELISpot assays. Donor control PBMCs were seeded at either 2 × 10^5^ cells per well for phytohemagglutinin (PHA)-stimulation (IL-10 assay) or 1 × 10^5^ cells per well for CEF-stimulation (IFN-γ assay). Stimulants were added to their corresponding wells using the following concentrations: 10 µg per mL ID93; 1 µg per mL RV1813, RV2608, RV3619, or RV3620 peptides; 1–10 µg per mL PHA; or 1 µg per mL CEF. Assembled plates were incubated overnight for 18–22 h at 37 °C and 5% CO_2_. Plates were then washed and incubated with 1 µg per mL of anti-human IFN-γ or anti-human IL-10 detection antibodies (Clone 7-B6-1, Cat# 3420-6-250, or Clone 12G8, Cat# 3430-6-250, respectively, MabTech) at 1000-fold dilution for 2.5 h at ambient temperature, washed, incubated with streptavidin-HRP for 1 h at ambient temperature, washed, and finally developed using NovaRED (Vector Laboratories, Newark, CA) for ~15 min at ambient temperature before stopping with distilled water. Within 1–2 days post-development, plates were scanned, counted, and subjected to quality control using the ImmunoSpot analyzer.

The mycobactericidal activity was studied in whole blood cultures from Day 0, 7, and 224 as previously described^29^. Briefly, RPMI 1640 medium with L-glutamine and HEPES (4-(2-hydroxyethyl)−1-piperazine ethanesulfonic acid) was used to dilute the heparinized whole blood samples, then these diluted whole blood samples were added in 2-mL tubes with BCG (in duplicates) and tightly capped. Then tubes were placed on a rotator and continuously rotated for 72 h at 37 °C. After the incubation, the tubes were centrifuged at 13,000 × *g* for 10 min, and the supernatant was removed. The remaining pellet from each tube was resuspended in PANTA-supplemented Mycobacteria Growth Indicator Tube (MGIT) medium (BD), vortexed thoroughly, and then transferred into a fluorimetric MGIT (BD). The MGITs with samples were tightly capped and placed into a Bactec MGIT 320 instrument (BD). The time to positivity (TTP) of MGITs was automatically tracked and recorded.

### Statistical analysis

The immunology analysis was performed using either Prism 9.3 or higher (GraphPad Software, San Diego, CA), SAS 9.4 (SAS Institute, Cary, NC), or R 4.03 (The R Foundation, Vienna, Austria). Categorical data were analyzed using Fisher’s exact test and a 0.05 level of significance (α = 0.05) with Bonferroni adjustments whenever pairwise comparisons were made between more than two categories.

Continuous data were compared between two treatment groups using the Wilcoxon rank-sum test since the normality assumption was not met for any dataset according to the Shapiro–Wilk test. Response rates were compared using Fisher’s exact test. Differences were detected based on a two-sided test and *p* ≤ 0.05 (α = 0.05) with Bonferroni adjustments whenever pairwise comparisons were made between multiple time points. Comparisons were accompanied by the point estimate and 95% confidence interval (CI) for each group. Missing data were assumed to be missing completely at random (MCAR), and analysis was performed on the available data.

IgG antibody responses (total IgG, IgG1, IgG2, IgG3, and IgG4) to the ID93 antigen were measured by ELISA using serum collected from a given participant. At each time point, the MEPT calculated from duplicates was reported. At each post-injection time point, the following Post:Pre Ratio was calculated:

1 $${{{{{\rm{Post}}}}}} \!\! :{{{{{\rm{Pre}}}}}}\,{{{{{\rm{Ratio}}}}}}=\frac{{{{{{\rm{MEPT}}}}}}({{{{{\rm{post}}}}}}-{{{{{\rm{injection}}}}}}\,{{{{{\rm{time}}}}}}\,{{{{{\rm{point}}}}}})}{{{{{{\rm{MEPT}}}}}}({{{{{\rm{baseline}}}}}}\,{{{{{\rm{time}}}}}}\,{{{{{\rm{point}}}}}})}$$

A participant was considered an antibody positive responder at a given time point if system suitability criteria for a given ELISA were met and the Post:Pre Ratio at that time point was ≥4.

Cellular responses to the ID93 protein were evaluated by measuring cytokine production in PBMCs by ELISpot assay and by ICS with flow cytometry. IFN-γ and IL-10 ELISpot assays were performed on samples from Study Days 0, 14, 56, 70, 84, and 224 to determine the proportion of PBMCs producing IFN-γ or IL-10 in response to stimulation with ID93 or unstimulated (PBS). For analysis, background-subtracted spot-forming units (SFU) values were calculated. At each post-injection time point, the change from baseline was calculated by subtracting the background-subtracted SFU value at baseline from a given time point. Both background-subtracted SFU and change from baseline SFU were summarized for each participant and visit day. The responder status at each time point was determined using the MIMOSA model, a Bayesian statistical framework that controls the false-discovery rate to 0.1%^30^. The Markov chain Monte Carlo (MCMC) settings used the default hyperparameter. Specifically, both non-responder and responder probabilities were assumed to follow the beta distribution where the hyperparameters were given vague exponential priors with a mean of 1000. The unknown proportion of responders was assumed to be drawn from a uniform distribution between 0 and 1. Each MCMC run used 200,000 iterations after 50,000 burn-in iterations. The responder rate was then calculated based on the total number of participants analyzed within each treatment group.

PBMC ICS was performed on samples from Study Days 0, 7, 14, 56, 63, 70, 84, and 224 to determine the proportion of single and multi-functional CD4 and CD8 T cells producing various combinations of IFNγ, TNF, IL-2, IL-4, IL-21, and CD154 in response to ID93 stimulation. A gate was applied for each functional marker, not accounting for the co-expression of other markers (Supplementary Figs. 6–10). Boolean gates were later created based on the gates shown to identify cells expressing various combinations of markers. We analyzed the T cell population for any two or more cytokines, which were positive for at least two immune markers among IFN-γ, TNF, IL-2, IL-4, IL-21, and CD154. We also evaluated T cells that expressed selected combinations of the six immune markers. Any negative background-subtracted values were set to zero. The background-subtracted frequency (%) was calculated as the frequency of cytokine-positive T cells for the ID93-stimulated samples minus the unstimulated samples (background subtracted).

2 $$\%{{{{{\rm{background}}}}}}{{{{{\rm{subtracted\; frequency}}}}}}=	\%{{{{{\rm{stimulated\; frequency}}}}}}\\ 	 -\%{{{{{\rm{background\; frequency}}}}}}$$

At each post-injection time point, the change from baseline was calculated by subtracting the background-subtracted frequency (%) at baseline from a given time point. Both background-subtracted and change from baseline frequencies were summarized for each participant and visit day. Response status was determined for each participant, visit day, and T cell subset using the MIMOSA model. The responder rate was then calculated based on the total number of participants analyzed within each treatment group.

### Reporting summary

Further information on research design is available in the Nature Portfolio Reporting Summary linked to this article.

## Supplementary information

Supplementary Information

Peer Review File

Reporting Summary

## Data Availability

The datasets generated during and/or analysed during the current study are available from the corresponding author upon reasonable request. However, individual participant data will not be available because informed consent did not explicitly include this. The study protocol, study participant disposition, and protocol deviations are included in the Supplementary Information. Source data are provided with this paper.

## Source data

Source Data
