# Supplementary material for: Safety and immunogenicity of a thermostable ID93 + GLA-SE tuberculosis vaccine candidate in healthy adults
Source: Nat Commun. 2023 Mar 6;14:1138. doi: 10.1038/s41467-023-36789-2 (PMC9988862; doi:10.1038/s41467-023-36789-2)
Supplement: Supplementary file 1 — Supplementary Information [file 41467_2023_36789_MOESM1_ESM.pdf]

Supplementary Information for "Safety and Immunogenicity of a Thermostable ID93 + GLA-SE Tuberculosis Vaccine Candidate in Healthy Adults" by Sagawa et al.

Supplementary Notes

List of clinical immunology antibodies and reagents

1. Serum ELISA

Protein

- ID93 Purified Bulk 70002-3 15JUN17/XJY Bottle 106 of 180 (AAHI)
- RV1813 #928-89.2 (AAHI)
- RV2608 Lot 1141-102 2.28.2019 (AAHI)
- RV3619#1065-16 Tris 8 (AAHI)
- RV3620 Lot 795-114 9/7/2015 (AAHI)

Control Sera

- Serum Study Samples
- Positive Serum 203-903-3006, 902-3004
- Negative Serum 203-903-3007, 903-2001
- Normal Human Serum Pool #11-14

Antibody-related Reagents

- rec-Protein G-Peroxidase Conjugate, Cat# 10-1223, Invitrogen
- Mouse anti-Human IgG1 Fc Secondary Ab HRP, Clone HP6070, Cat# MH1715, Invitrogen (1000-fold dilution)
- Mouse anti-Human IgG2 Fd Secondary Ab HRP, Clone HP6014, Cat# 05-0520, Invitrogen (500-fold dilution)
- Mouse anti-Human IgG3 (Hinge) Secondary Ab HRP, Clone HP6047, Cat# 53620, Invitrogen (1000-fold dilution)
- Mouse anti-Human IgG4 Fc, Secondary Ab HRP, Clone HP6023, Cat# MH1742, Invitrogen (500-fold dilution)
- Protein G purified Human IgG (Lot# 12621, ABL)
- Ultra Leaf Human IgG2 Isotype Control Recombinant Antibody, Clone QA16A13, Cat# 403602, BioLegend (1000-fold dilution)

- Ultra Leaf Human IgG4 Isotype Control Recombinant Antibody, Clone QA16A15, Cat# 403702, BioLegend (2350-fold dilution)

2. B Cell ELISpot

Protein

- ID93 Purified Bulk 70002-3 15JUN17/XJY Bottle 106 of 180 (AAHI)

PBMCs

- PBMC Study Samples
- Standard cryopreserved (LN_2) human PBMC sample supplied by ABL (TB history unknown)

Critical Reagents

- IgG Capture Antibody, Clone MT91/145, Cat# 3850-3-1000, MabTech (100-fold dilution)
- IgA Capture Antibody, Clone MT57, Cat# 3860-3-1000, MabTech (100-fold dilution)
- Biotinylated IgG Detection Ab, Clone MT78/145, Cat# 3850-6-250, MabTech (500-fold dilution)
- Biotinylated IgA Detection Ab, Clone MT20, Cat# 3860-6-250, MabTech (500-fold dilution)

3. T Cell ELISpot

Protein

- ID93 Purified Bulk 70002-3 15JUN17/XJY Bottle 106 of 180 (AAHI)
- Peptide Pools RV2608, RV1813, RV3619, RV3620RV (JPT Peptide Technologies)

PBMCs

- PBMC Study Samples
- CEFT Donor PBMCs (#1063678, Bloodworks Northwest)

Critical Reagents

- PHA-P, Cat# inh-phap, InvivoGen (replacement for PHAP [Murex], Remel)
- CEF Pool Extended, Cat# PM-CEF-E-2, JPT Technologies
- IFN-γ Mouse anti-human Capture Antibody, Clone 1-D1K, Cat# 3420-3-250, MabTech (100-fold dilution)
- IL-10 Mouse anti-human Capture Antibody, Clone 9D7, Cat# 3430-3-250, MabTech (100-fold dilution)
- IFN-γ Biotinylated Mouse anti-human Detection Ab, Clone 7-B6-1, Cat# 3420-6-250, MabTech (1000-fold dilution)
- IL-10 Biotinylated Mouse anti-human Detection Ab, Clone 12G8, Cat# 3430-6-250, MabTech (1000-fold dilution)
- NovaRed substrate, Cat# SK-4800, Vector Laboratory
- Streptavidin-HRP, Cat# 3310-9-1000, MabTech

4. ICS

Protein

- ID93 Purified Bulk 70002-3 15JUN17/XJY Bottle 106 of 180 (AAHI)

PBMCs

- PBMC Study Samples
- CEFT Donor PBMCs (#W31374034473, HemaCare BioResearch Products)

Critical Reagents

- Live/Dead Aqua (AviD) BV510 (Reconstituted), Cat# L34957, Invitrogen
- Human CD3 R718, Clone SP34-2, Cat# 566955, BD Biosciences (25-fold dilution)
- Human CD4 BUV395, Clone SK3, Cat# 563550, BD Biosciences (25-fold dilution)
- Human CD8 BV711, Clone RPA-T8, Cat# 563677, BD Biosciences (25-fold dilution)
- Human CD14 BV510, Clone M5E2, Cat# 301842, BioLegend (20-fold dilution)
- Human CD45RA APC-Cy7, Clone HI100, Cat# 560674, BD Biosciences (40-fold dilution)
- Human CD56 BV650, Clone HCD56, Cat# 318344, BioLegend (50-fold dilution)
- Human CD154 BUV496, Clone TRAP1, Cat# 750407, BD Biosciences (40-fold dilution)

- Human CD197 (CCR7) BV785, Clone G043H7, Cat# 353230, BioLegend (10-fold dilution)
- Mouse anti-Human CD279 (PD-1) PE-Cy7, Clone eBioJ105, Cat# 25-2799-42, eBioscience (10-fold dilution)
- Human CXCR5 PE-eFluor 610 (PE-Dazzle), Clone MU5UBEE, Cat# 61-9185-42, eBioscience (20-fold dilution)
- Human IL-2 PE, Clone MQ1-17H12, Cat# 559334, BD Biosciences (10-fold dilution)
- Human IL-4 PerCP-Cy5.5, Clone MP4-25D2, Cat# 500822, BioLegend (40-fold dilution)
- Human IL-21 APC, Clone 3A3-N2, Cat# 513008, BioLegend (40-fold dilution)
- Human IFN-γ BV421, Clone B27, Cat# 560371, BD Biosciences (25-fold dilution)
- Human TNF-α FITC, Clone MAb11, Cat# 11-7349-82, eBioscience (40-fold dilution)
- Human CD49d, Clone 9F10, Cat# 16-0499-85, Invitrogen
- Human CD28, Clone CD28.2, Cat# 16-0289-85, eBioscience
- TruStain FcX, Cat# 422302, BioLegend
- BV Brilliant Stain, Cat# 563794, BD Biosciences
- Guava EasyCheck Kit, Cat# 4200-0025, Luminex
- Guava ViaCount, Cat# 4000-0041, Luminex
- 1X DPBS w/o Ca^{2+} and Mg^{2+}, Cat# 114-057-131, Quality Biological
- Cell Culture Water, Cat# 118-162-101, Quality Biological
- RPMI 1640 Media, Cat# R5886-500ML, Sigma-Aldrich
- FBS, Cat# SH30070.03, HyClone
- L-Glutamine, Cat# 25030-081, Gibco
- Pen-Strep, Cat# 15140-122, Gibco
- Benzonase, Cat# 71206-3, Millipore Sigma
- Brefeldin A, Cat# 00-4506-51, ThermoFisher
- SEB (Reconstituted), Cat# 324-798-500UG, Millipore Sigma
- Cytofix/CytoPerm, Cat# 554722, BD Biosciences
- 10X Perm/Wash, Cat# 554723, BD Biosciences
- Stabilizing Fixative, Cat# 338036, BD Biosciences
- Ultracomp Compensation Beads, Cat# 01-3333-42, Invitrogen/ThermoFisher
- ArC Reactive Beads, Cat# A10628, Invitrogen/ThermoFisher
- ArC Negative Beads, Cat# A10628, Invitrogen/ThermoFisher

5. Mucosal ELISA and MGIT Reagents

- ID93 Purified Protein (Lot# 700002-3, AAHI)
- BSA, Cat# 12659, MilliporeSigma
- Biotinylated Anti-Hu-IgA (alpha chain) Ab, Cat# 5260-0027, KPL, Seracare Life Sciences (500-fold dilution)
- Streptavidin, Phosphatase-Labeled, Cat# 5270-0030, KPL, Seracare Life Sciences

- SIGMAFAST p-NPP Tablets, Cat# N1891, MilliporeSigma
- APStop Solution (10X), Cat# 5150-0026, KPL, Seracare Life Sciences
- IgA (Human) ELISA Kit, Cat# KA2110, Abnova
- Gibco RPMI 1640, Cat# 11875-085, Life Technologies
- Bacillus Calmette-Guérin (BCG) Pasteur (Saint Louis University, St. Louis, MO)
- BACTEC™ MGIT™ Tubes, Cat# 245122, BD
- BACTEC MGIT Growth Supplement (OADC+PANTA) Kit, Cat# 245124

Supplementary Table 1. Antibody Responses to ID93 as measured by serum IgG ELISA

	**ID93 + GLA-SE (single-vial)**	**ID93 + GLA-SE (two-vial)**	**Adjusted *p*-value**
Study Day 0	(*n* = 23)	(*n* = 25)	
Geometric mean MEPT	57 (49, 66)	74 (45, 123)	1
Study Day 14	(*n* = 23)	(*n* = 24)	
Geometric mean MEPT	147 (83, 260)	140 (94, 209)	1
Geometric mean Post:Pre Ratio	2.6 (1, 5)	1.9 (1, 3)	1
Responder rate	30.4 (13.21, 52.92)	25.0 (9.77, 46.71)	1
Study Day 56	(*n* = 23)	(*n* = 22)	
Geometric mean MEPT	250 (154, 405)	259 (126, 530)	1
Geometric mean Post:Pre Ratio	4.4 (3, 7)	3.3 (1, 8)	1
Responder rate	60.9 (38.54, 80.29)	40.9 (20.71, 63.65)	1
Study Day 70	(*n* = 23)	(*n* = 22)	
Geometric mean MEPT	10868 (5971, 19780)	2583 (1608, 4147)	0.0061**
Geometric mean Post:Pre Ratio	190.5 (106, 343)	33 (16, 70)	0.0023**
Responder rate	100 (85.18, 100.00)	90.9 (70.84, 98.88)	1
Study Day 84	(*n* = 22)	(*n* = 22)	
Geometric mean MEPT	5592 (2753, 11362)	2522 (1711, 3716)	0.1415
Geometric mean Post:Pre Ratio	97.4 (49, 193)	32.2 (17, 63)	0.0495*
Responder rate	90.9 (70.84, 98.88)	95.5 (77.16, 99.88)	1
Study Day 224	(*n* = 20)	(*n* = 22)	
Geometric mean MEPT	913 (510, 1634)	445 (273, 726)	0.3857
Geometric mean Post:Pre Ratio	15.7 (9, 28)	5.7 (3, 12)	0.1661
Responder rate	85.0 (62.11, 96.79)	63.6 (40.66, 82.80)	0.829

Parentheticals shown are 95% confidence interval. For MEPT and Post:Pre Ratio comparisons, the Wilcoxon rank-sum test was used. The responder rate comparisons were performed using the Fisher's exact test. Differences were detected based on a two-sided test and $p \leq 0.05$ ($\alpha = 0.05$). The *p*-values for the MEPT normality test (Shapiro-Wilk test) were <0.0001 (single-vial); the *p*-values for Post:Pre Ratio normality test (Shapiro-Wilk test) were 0.0037 (single-vial) and 0.0017 (two-vial); thus the non-parametric Wilcoxon rank-sum test was used for their comparisons. The multiplicity-adjusted *p*-values were calculated using the Bonferroni method to account for the multiple time points. *multiplicity-adjusted *p*-value < 0.05 compared to non-thermostable two-vial presentation at the same time point; **multiplicity-adjusted *p*-value < 0.01 compared to non-thermostable two-vial presentation at the same time point.

Supplementary Table 2. IFN-γ responses to ID93 as measured by PBMC ELISpot

	**ID93 + GLA-SE (single-vial)**	**ID93 + GLA-SE (two-vial)**	**Adjusted *p*-value**
Study Day 0	(*n* = 23)	(*n* = 25)	
Mean background-subtracted SFUs	10.73 (-0.567, 22.028)	8.068 (3.197, 12.939)	1
Responder rate	13.0 (2.78, 33.59)	20.0 (6.83, 40.70)	1
Study Day 14	(*n* = 22)	(*n* = 25)	
Mean background-subtracted SFUs	21.241 (7.7, 34.782)	11.596 (5.064, 18.128)	1
Responder rate	45.5 (24.39, 67.79)	32.0 (14.95, 53.50)	1
Study Day 56	(*n* = 23)	(*n* = 22)	
Mean background-subtracted SFUs	13.722 (5.744, 21.699)	10.995 (6.283, 15.708)	1
Responder rate	34.8 (16.38, 57.27)	31.8 (13.86, 54.87)	1
Study Day 70	(*n* = 23)	(*n* = 21)	
Mean background-subtracted SFUs	55.139 (27.786, 82.493)	28.114 (14.774, 41.454)	1
Responder rate	69.6 (47.08, 86.79)	76.2 (52.83, 91.78)	1
Study Day 84	(*n* = 22)	(*n* = 21)	
Mean background-subtracted SFUs	45.027 (21.883, 68.172)	35.271 (10.623, 59.92)	1
Responder Rate	68.2 (45.13, 86.14)	66.7 (43.03, 85.41)	1
Study Day 224	(*n* = 20)	(*n* = 19)	
Mean background-subtracted SFUs	29.165 (12.704, 45.626)	17.179 (9.187, 25.171)	1
Responder rate	65.0 (40.78, 84.61)	52.6 (28.86, 75.55)	1

Parentheticals shown are 95% confidence intervals. Colony count comparisons were performed using the Wilcoxon rank-sum test. The responder rate comparisons were performed using the Fisher's exact test. Differences were detected based on a two-sided test and $p \leq 0.05$ ($\alpha = 0.05$). The *p*-values for the SFU count normality test (Shapiro-Wilk test) were <0.0001 (single-vial) and <0.0001 (two-vial); thus, the non-parametric Wilcoxon rank-sum test was used for the comparisons. Adjusted *p*-values were calculated using the Bonferroni method.

Supplementary Table 3. IL-10 responses to ID93 as measured by PBMC ELISpot

	ID93 + GLA-SE (single-vial)	ID93 + GLA-SE (two-vial)	Adjusted *p*-value
Study Day 0	(*n* = 23)	(*n* = 25)	
Mean background-subtracted SFUs	2.387 (1.01, 3.764)	3.148 (1.278, 5.018)	1
Responder rate	4.3 (0.11, 21.95)	8.0 (0.98, 26.03)	1
Study Day 14	(*n* = 22)	(*n* = 23)	
Mean background-subtracted SFUs	3.432 (1.833, 5.031)	2.652 (1.068, 4.237)	1
Responder rate	0 (0.00, 15.44)	4.3 (0.11, 21.95)	1
Study Day 56	(*n* = 23)	(*n* = 22)	
Mean background-subtracted SFUs	4.443 (1.364, 7.523)	3.305 (1.41, 5.199)	1
Responder rate	4.3 (0.11, 21.95)	4.5 (0.12, 22.84)	1
Study Day 70	(*n* = 23)	(*n* = 20)	
Mean background-subtracted SFUs	13.583 (1.763, 25.402)	3.47 (2.019, 4.921)	0.7404
Responder rate	30.4 (13.21, 52.92)	0 (0.00, 16.84)	0.0601
Study Day 84	(*n* = 22)	(*n* = 19)	
Mean background-subtracted SFUs	6.8 (3.034, 10.566)	4.853 (2.248, 7.457)	1
Responder rate	18.2 (5.19, 40.28)	10.5 (1.30, 33.14)	1
Study Day 224	(*n* = 20)	(*n* = 18)	
Mean background-subtracted SFUs	4.205 (1.921, 6.489)	2.633 (0.712, 4.555)	1
Responder rate	10.0 (1.23, 31.70)	0 (0.00, 18.53)	1

Parentheticals shown are 95% confidence intervals. SFU count comparisons were performed using the Wilcoxon rank-sum test. The responder rate comparisons were performed using the Fisher's exact test. Differences were detected based on a two-sided test and $p \leq 0.05$ ($\alpha = 0.05$). The *p*-values for SFU count normality test (Shapiro-Wilk test) were <0.0001 (single-vial) and <0.0001 (two-vial); thus, the non-parametric Wilcoxon rank-sum test was used for the comparisons. Adjusted *p*-values were calculated using the Bonferroni method.

Supplementary Table 4. CD4$^+$ T cell responses to ID93 as measured by PBMC ICS

	ID93 + GLA-SE (single-vial)	ID93 + GLA-SE (two-vial)	Adjusted *p*-value
Study Day 0	(*n* = 23)	(*n* = 25)	
Mean frequency of Any Two cytokines	0.038 (-0.028, 0.104)	0.035 (-0.02, 0.089)	1
Responder rate	4.3 (0.11, 21.95)	4.0 (0.10, 20.35)	1
Study Day 7	(*n* = 21)	(*n* = 25)	
Mean frequency of Any Two cytokines	1.256 (-1.355, 3.866)	0.015 (-0.004, 0.035)	1
Responder rate	4.8 (0.12, 23.82)	4.0 (0.10, 20.35)	1
Study Day 14	(*n* = 20)	(*n* = 24)	
Mean frequency of Any Two cytokines	1.527 (-0.317, 3.371)	0.024 (-0.015, 0.063)	0.2243
Responder rate	15.0 (3.21, 37.89)	4.2 (0.11, 21.12)	1
Study Day 56	(*n* = 22)	(*n* = 20)	
Mean frequency of Any Two cytokines	0.22 (-0.192, 0.633)	0.002 (-0.002, 0.005)	0.1384
Responder rate	13.6 (2.91, 34.91)	0 (0.00, 16.84)	1
Study Day 63	(*n* = 22)	(*n* = 22)	
Mean frequency of Any Two cytokines	0.187 (0.067, 0.308)	0.47 (-0.299, 1.239)	0.1604
Responder rate	36.4 (17.20, 59.34)	13.6 (2.91, 34.91)	0.9737
Study Day 70	(*n* = 22)	(*n* = 21)	
Mean frequency of Any Two cytokines	0.404 (0.202, 0.605)	0.214 (0.055, 0.372)	0.4242
Responder rate	59.1 (36.35, 79.29)	38.1 (18.11, 61.56)	1
Study Day 84	(*n* = 20)	(*n* = 20)	
Mean frequency of Any Two cytokines	0.225 (0.121, 0.329)	0.688 (-0.508, 1.883)	1
Responder rate	50.0 (27.20, 72.80)	30.0 (11.89, 54.28)	1
Study Day 224	(*n* = 16)	(*n* = 19)	
Mean frequency of Any Two cytokines	0.696 (-0.586, 1.977)	0.419 (-0.046, 0.884)	1
Responder rate	31.3 (11.02, 58.66)	26.3 (9.15, 51.20)	1

Parentheticals shown are 95% confidence intervals. Mean frequency of Any Two cytokines represents mean frequency of Any Two cytokine positives. Mean frequency comparisons were performed using the Wilcoxon rank-sum test. The responder rate comparisons were performed using the Fisher's exact test. Differences were detected based on a two-sided test and $p \leq 0.05$ ($\alpha = 0.05$). The *p*-values for mean frequency normality test (Shapiro-Wilk test) were <0.0001 (single vial) and <0.0001 (two vials); thus, the non-parametric Wilcoxon rank-sum test was used for the comparisons. Adjusted *p*-values were calculated using the Bonferroni method.

Supplementary Table 5. CD8^{+} T cell response to ID93 as measured by PBMC ICS

	ID93 + GLA-SE (single-vial)	ID93 + GLA-SE (two-vial)	Adjusted *p*-value
Study Day 0	(*n* = 23)	(*n* = 25)	
Mean frequency of Any Two cytokines	0.019 (-0.001, 0.039)	0.018 (0.001, 0.035)	1
Responder rate	0 (0.00, 14.82)	0 (0.00, 13.72)	1
Study Day 7	(*n* = 21)	(*n* = 25)	
Mean frequency of Any Two cytokines	0.334 (-0.35, 1.018)	0.014 (0.002, 0.027)	1
Responder rate	4.8 (0.12, 23.82)	0 (0.00, 13.72)	1
Study Day 14	(*n* = 20)	(*n* = 24)	
Mean frequency of Any Two cytokines	0.601 (-0.244, 1.446)	0.014 (-0.001, 0.029)	0.7515
Responder rate	15.0 (3.21, 37.89)	0 (0.00, 14.25)	0.5165
Study Day 56	(*n* = 22)	(*n* = 20)	
Mean frequency of Any Two cytokines	0.26 (-0.264, 0.783)	0.007 (0.001, 0.013)	1
Responder rate	4.5 (0.12, 22.84)	0 (0.00, 16.84)	1
Study Day 63	(*n* = 22)	(*n* = 22)	
Mean frequency of Any Two cytokines	0.032 (-0.002, 0.066)	0.067 (-0.033, 0.167)	1
Responder rate	4.5 (0.12, 22.84)	9.1 (1.12, 29.16)	1
Study Day 70	(*n* = 22)	(*n* = 21)	
Mean frequency of Any Two cytokines	0.006 (0, 0.012)	0.034 (0.004, 0.065)	0.5714
Responder rate	0 (0.00, 15.44)	4.8 (0.12, 23.82)	1
Study Day 84	(*n* = 20)	(*n* = 20)	
Mean frequency of Any Two cytokines	0.005 (0, 0.01)	0.119 (-0.12, 0.358)	1
Responder rate	0 (0.00, 16.84)	5.0 (0.13, 24.87)	1
Study Day 224	(*n* = 16)	(*n* = 19)	
Mean frequency of Any Two cytokines	0.292 (-0.286, 0.87)	0.106 (-0.025, 0.237)	1
Responder rate	6.3 (0.16, 30.23)	15.8 (3.38, 39.58)	1

Parentheticals shown are 95% confidence intervals. Mean frequency of Any Two cytokines represents mean frequency of Any Two cytokine positives. Mean frequency comparisons were performed using the Wilcoxon rank-sum test. The responder rate comparisons were performed using the Fisher's exact test. Differences were detected based on a two-sided test and $p \leq 0.05$ ($\alpha = 0.05$). The *p*-values for mean frequency normality test (Shapiro-Wilk test) were <0.0001 (single-vial) and <0.0001 (two-vial); thus, the non-parametric Wilcoxon rank-sum test was used for the comparisons. Adjusted *p*-values were calculated using the Bonferroni method.

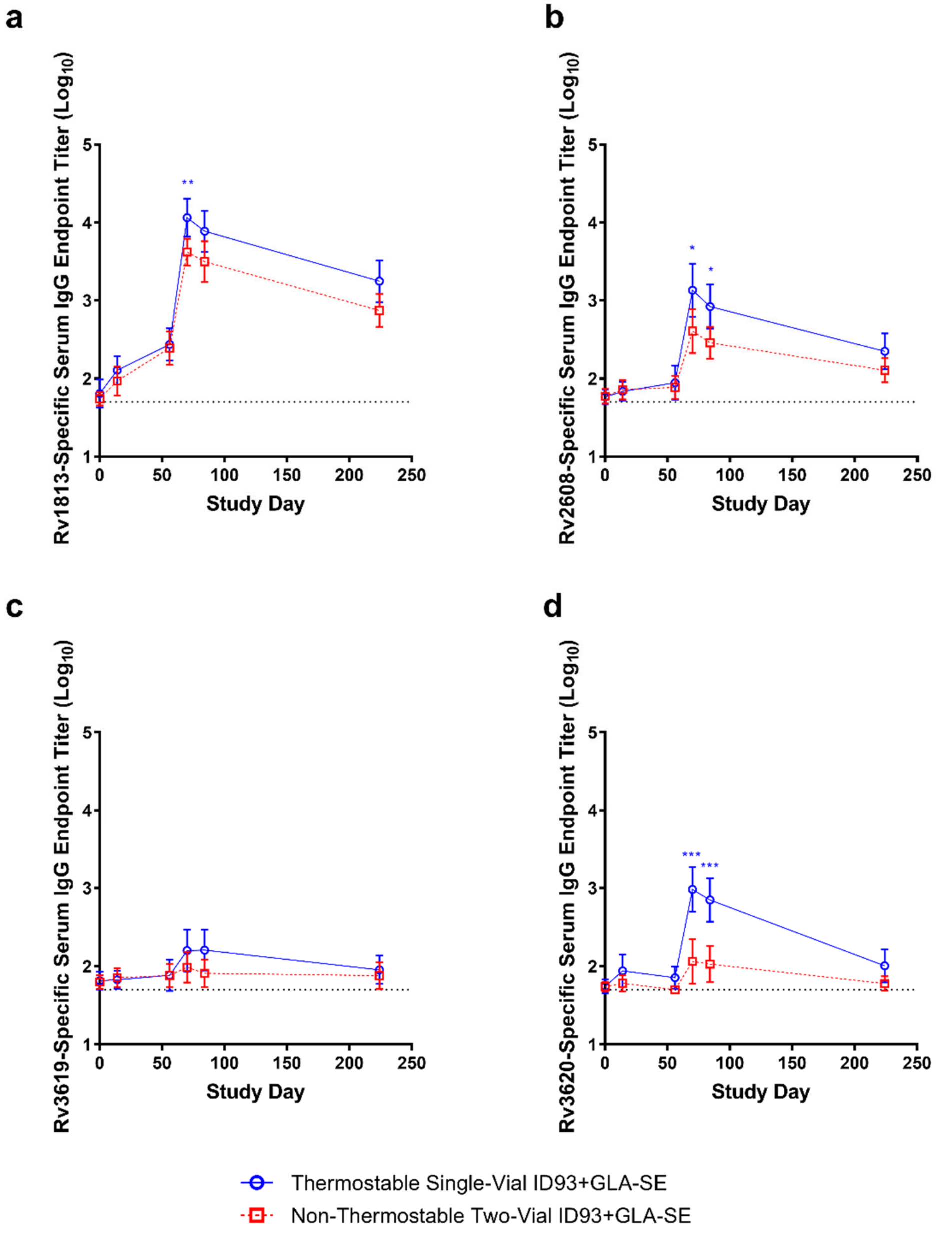

Supplementary Figure 1. ID93 subunit-specific serum antibody responses by ELISA. *n*=19-25 biologically independent samples depending on treatment group and time point. Longitudinal plots of group MEPT for total IgG specific for ID93 subunits. **a** Rv1813. ***p* = 0.0095. **b** Rv2608. **p* = 0.0490 (Study Day 70), **p* = 0. 0.0249 (Study Day 84). **c** Rv3619. **d** Rv3620. *** *p* = 0.0001 (Study Day 70), *** *p* = 0.0002 (Study Day 84). Error bars show the 95% CI on the mean. Data were compared between the two treatment groups using the Wilcoxon rank-sum test with Bonferroni adjustment to account for the multiple time points. Asterisks indicate statistically significant difference (**p* < 0.05, ***p* < 0.01, *** *p* < 0.001) between treatment groups at that time point after correction for multiple comparisons. Source data are provided as a Source Data file.

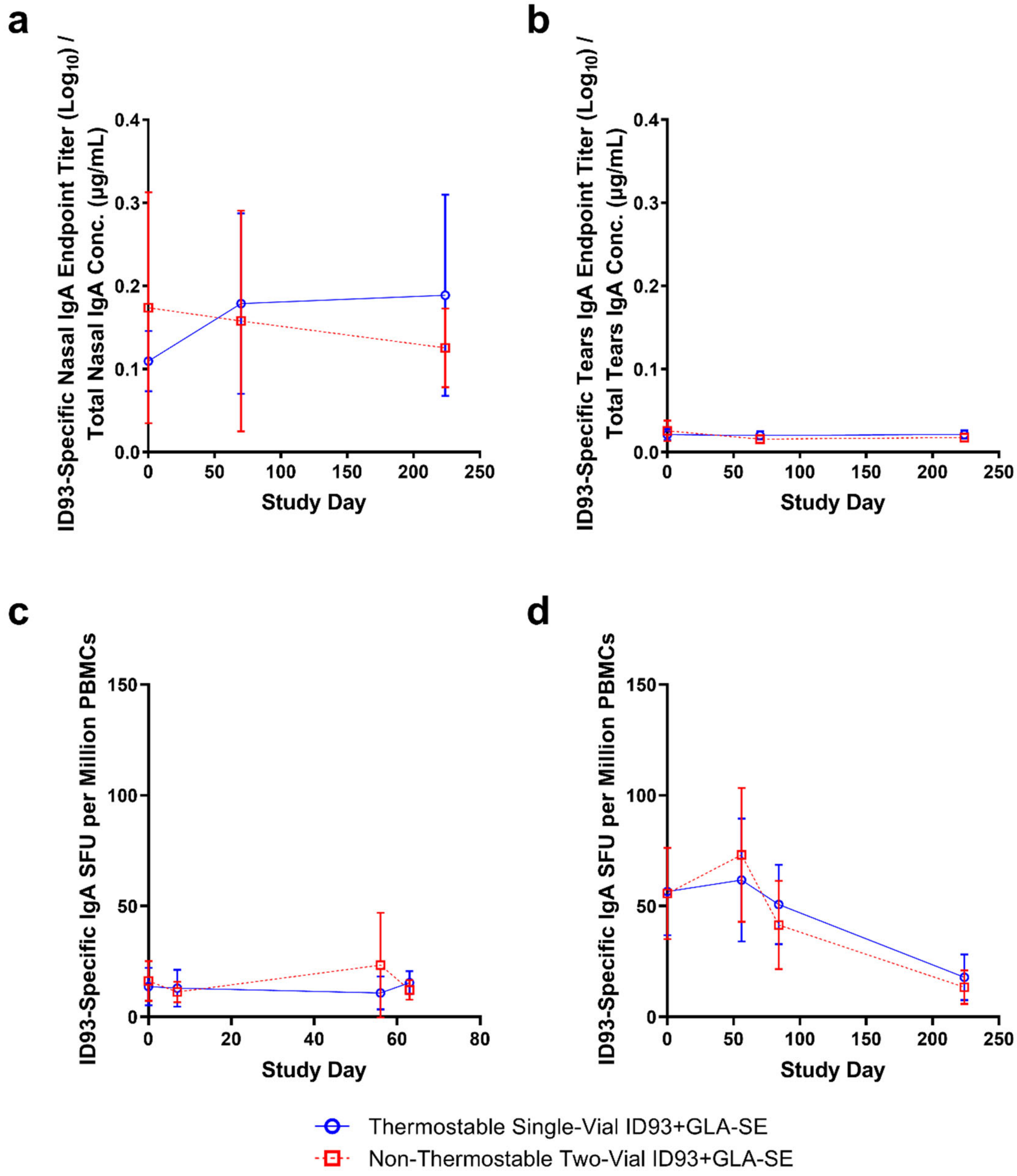

Supplementary Figure 2. Group comparison of ID93-specific mucosal antibody responses by ELISA and B cell responses by ELISpot assay. *n*=18-25 biologically independent samples depending on treatment group and time point. **a** Longitudinal plot of group MEPT for ID93-specific IgA ratioed to non-specific IgG concentration in nasal swabs as measured by ELISA. **b** Longitudinal plot of group MEPT for ID93-specific IgA ratioed to non-specific IgG concentration in tear samples as measured by ELISA. **c** Enumeration of ID93-specific IgA-secreting cells in PBMC samples as measured by ELISpot assay. **d** Enumeration of ID93-specific IgA memory B cells in PBMC samples as measured by ELISpot assay. Mean values are represented by symbols, and error bars show the 95% CI on the mean. Data were compared between the two treatment groups using the Wilcoxon rank-sum test with Bonferroni adjustment to account for the multiple time points. Source data are provided as a Source Data file.

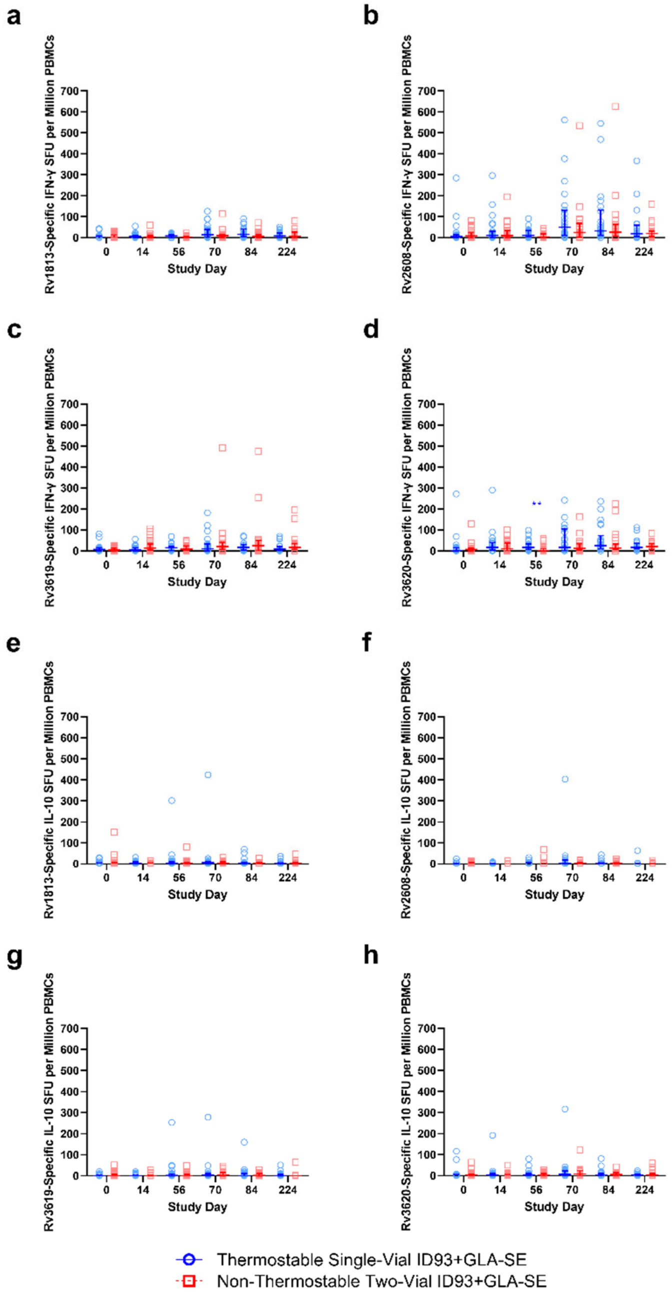

Supplementary Figure 3. T Cell ELISpot with ID93 subunit peptide pool stimulation. *n*=18-25 biologically independent samples depending on treatment group and time point. **a-d** Enumeration of antigen subunit peptide pool-stimulated IFN-γ-secreting cells in PBMC samples. **e-h** Enumeration of antigen subunit peptide-pool-stimulated IL-10-secreting cells in PBMC samples. Dot plots represent all background-subtracted values as symbols, with solid lines representing median values and error bars representing the interquartile range. Data were compared between the two treatment groups using the Wilcoxon rank-sum test with Bonferroni adjustment to account for the multiple time points. ***p* = 0.0087. Source data are provided as a Source Data file.

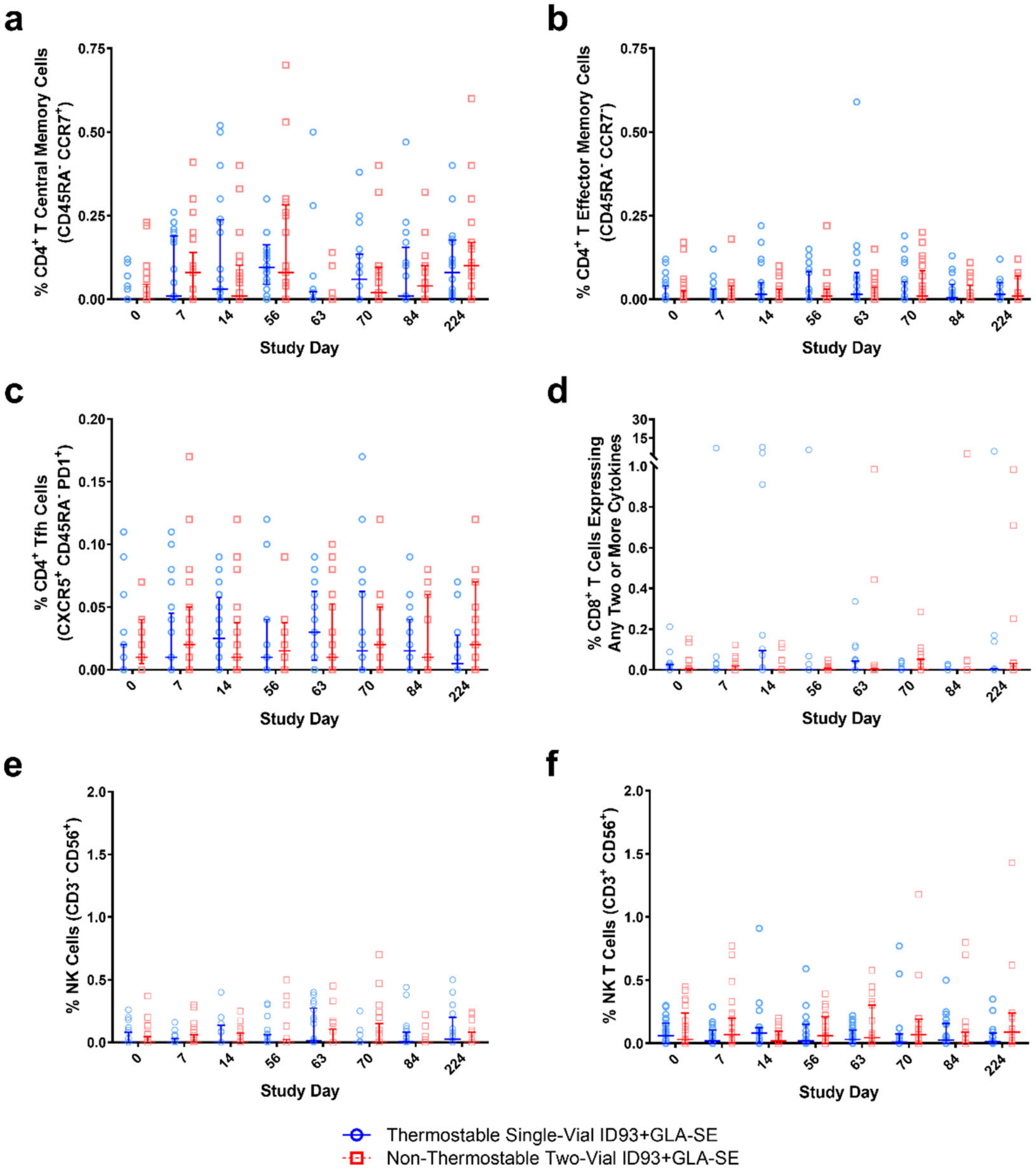

Supplementary Figure 4. Cellular immune response phenotypes in PBMC samples by ICS with flow cytometry. *n*=16-25 biologically independent samples depending on treatment group and time point. **a** Frequencies of ID93-stimulated CD4^{+} T central memory cells in PBMC samples. **b** Frequencies of ID93-stimulated CD4^{+} T effector memory cells in PBMC samples. **c** Frequencies of ID93-stimulated CD4^{+} Tfh cells in PBMC samples. **d** Frequencies of ID93-stimulated CD8^{+} T cells expressing Any Two cytokines in PBMC samples. **e** Frequencies of ID93-stimulated NK cells in PBMC samples. **f** Frequencies of ID93-stimulated NK T cells in PBMC samples. All background-subtracted values are represented as symbols, with solid lines representing median values and error bars representing the interquartile range. Data were compared between the two treatment groups using the Wilcoxon rank-sum test with Bonferroni adjustment to account for the multiple time points. Source data are provided as a Source Data file.

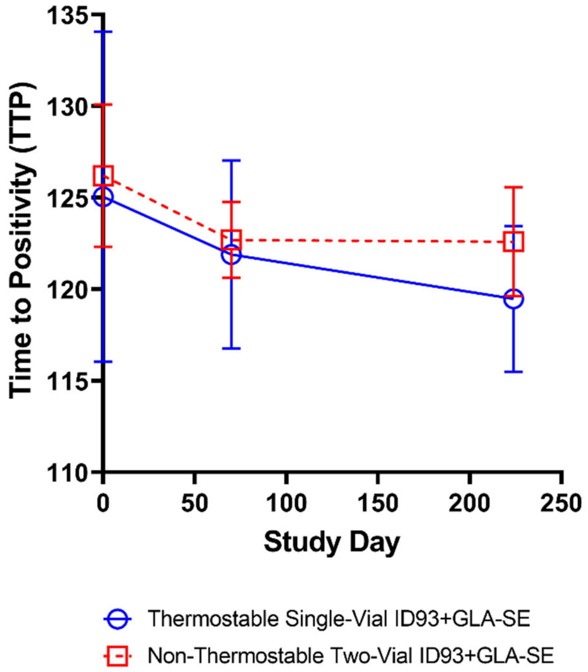

Supplementary Figure 5. Group comparison of whole blood mycobacterial growth inhibition. Longitudinal plot of time to positivity in mycobacterial growth inhibition assay. *n*=19-25 biologically independent samples depending on treatment group and time point. Mean values are represented by symbols and error bars show the 95% CI on the mean. Data were compared between the two treatment groups using the Wilcoxon rank-sum test with Bonferroni adjustment to account for the multiple time points. Source data are provided as a Source Data file.

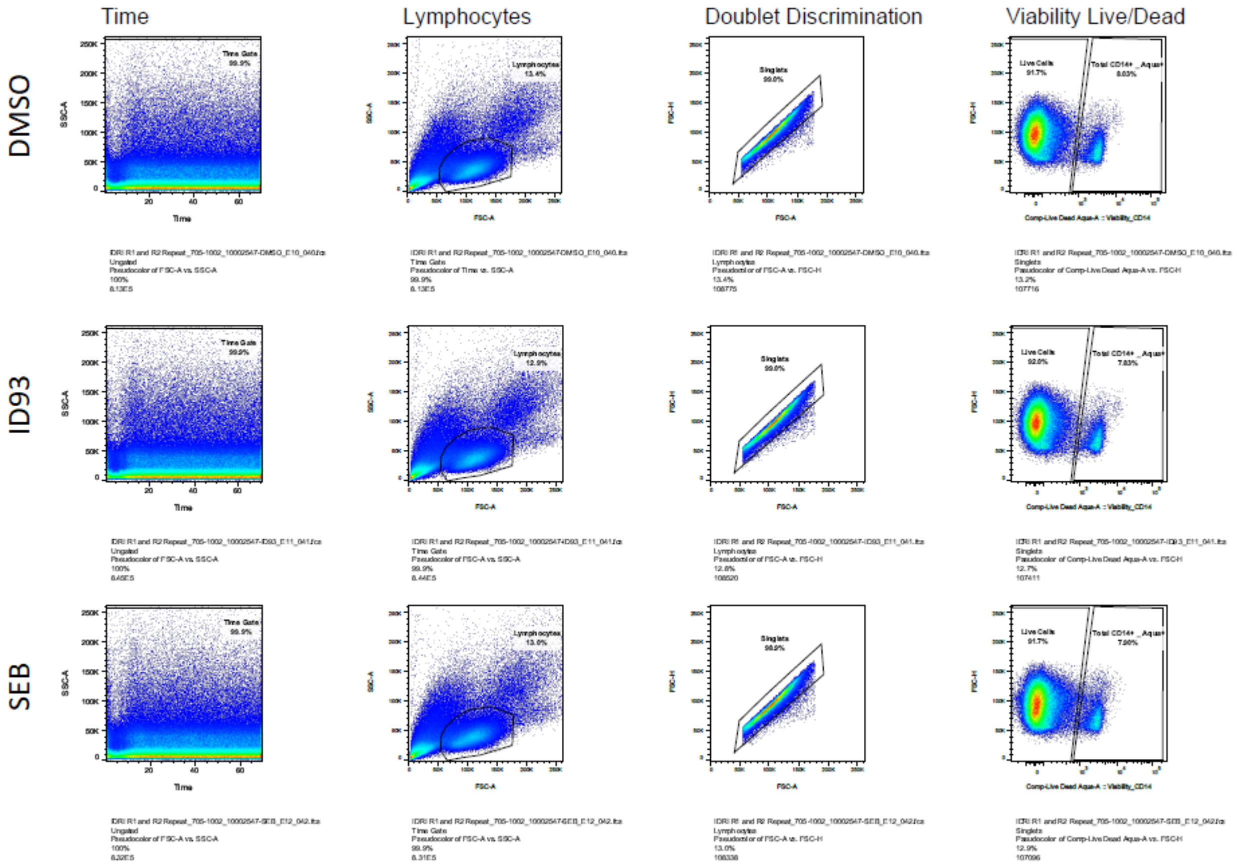

Supplementary Figure 6. Flow cytometry gating strategy and cell population percentages: live cell gating. Comparison between cells that were stimulated with either DMSO (negative control), ID93 antigen, or Staphylococcal enterotoxin B (SEB; positive control). Live/dead staining was conducted using the CD14 viability dye.

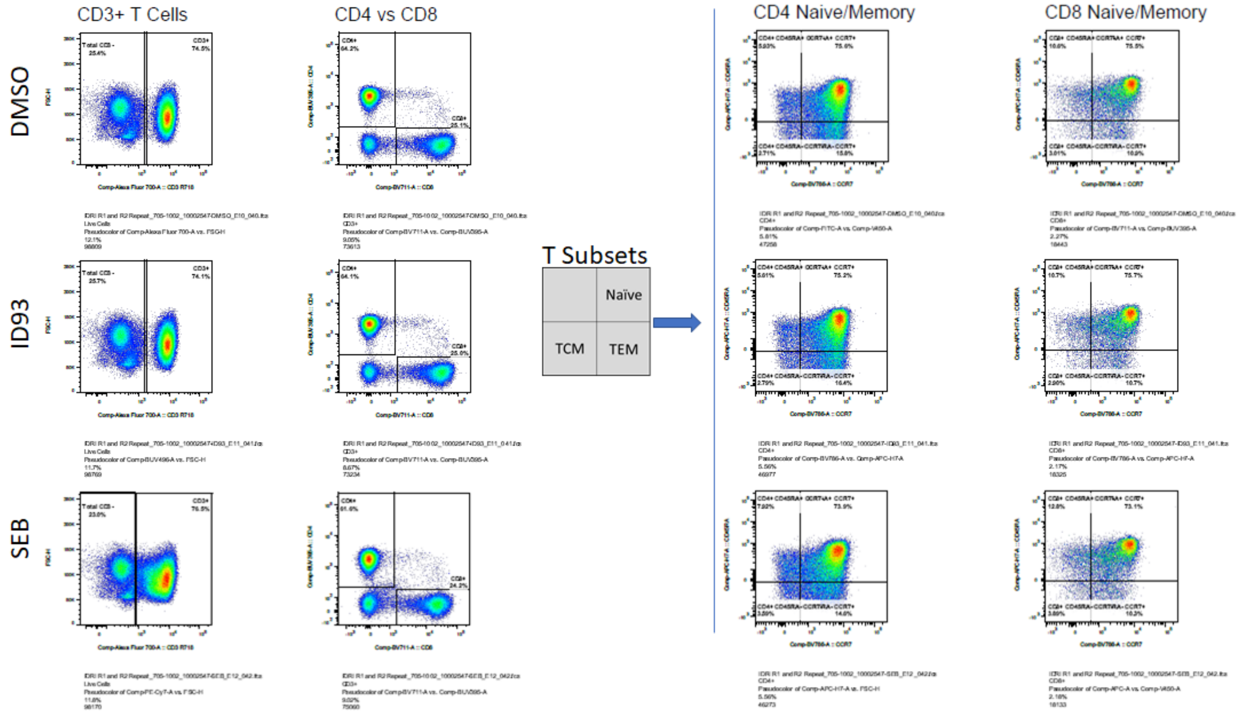

Supplementary Figure 7. Flow cytometry gating strategy and cell population percentages: CD3$^+$, CD4 T cells, CD8 T cells, and T cell subsets naïve, T central memory cells (TCM), and T effector memory cells (TEM). Biomarkers for naïve T cells, TCM, and TEM were CD3$^+$CD4$^+$CD45RA$^-$CCR7$^+$ and CD3$^+$CD8$^+$CD45RA$^-$CCR7$^+$, CD3$^+$CD4$^+$CD45RA$^-$CCR7$^+$ and CD3$^+$CD8$^+$CD45RA$^-$CCR7$^+$, and CD3$^+$CD4$^+$CD45RA$^-$CCR7$^-$ and CD3$^+$CD8$^+$CD45RA$^-$CCR7$^-$, respectively.

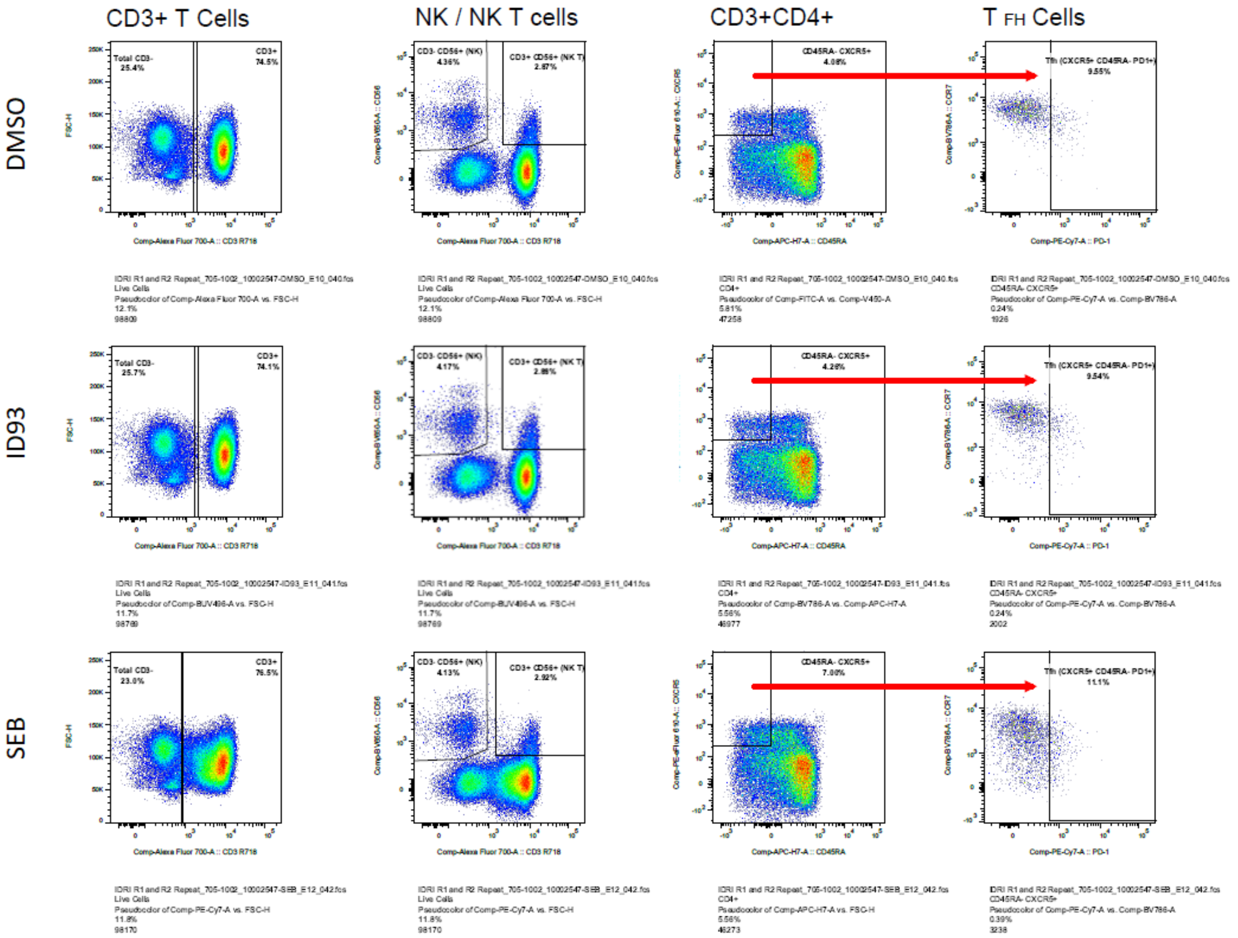

Supplementary Figure 8. Flow cytometry gating strategy and cell population percentages: natural killer (NK) ($CD3^{-}CD56^{+}$), NK T ($CD3^{+}CD56^{+}$), and T^{FH} follicular helper ($CD3^{+}CD4^{+}CD45RA^{-}CXCR5^{+}PD1^{+}$) cells.

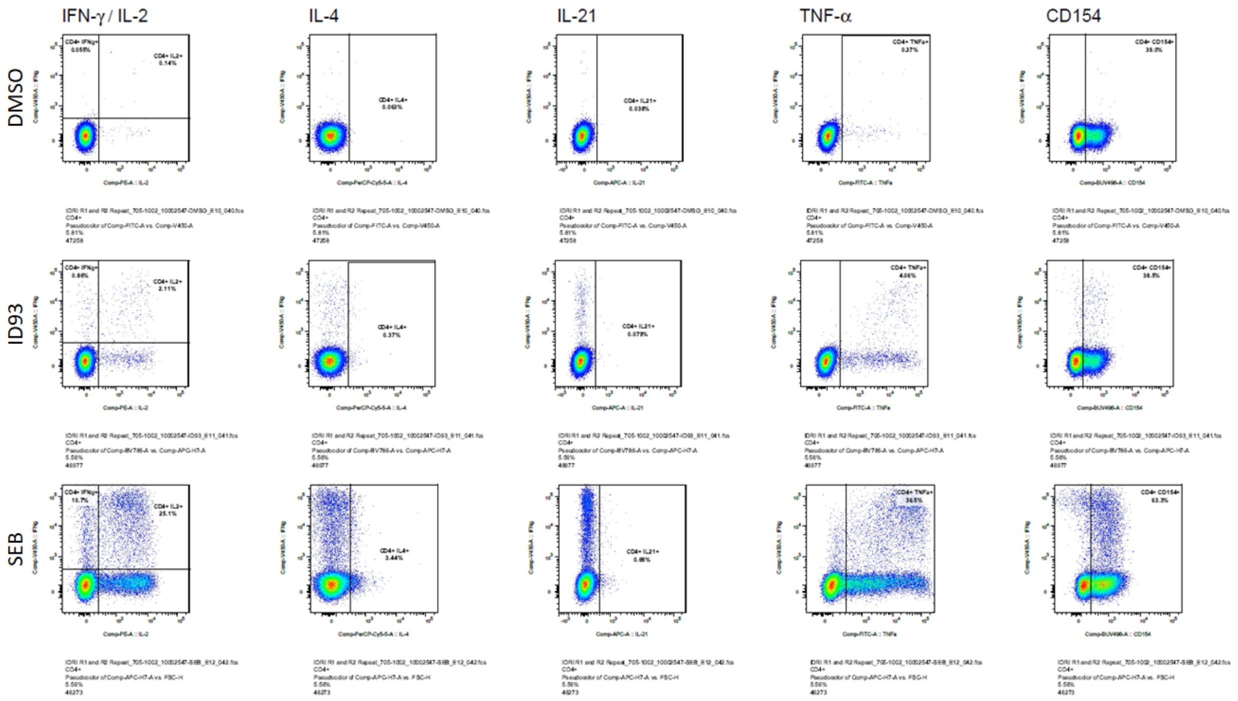

Supplementary Figure 9. Flow cytometry gating strategy and cell population percentages: cytokine expression among CD4 T cells.

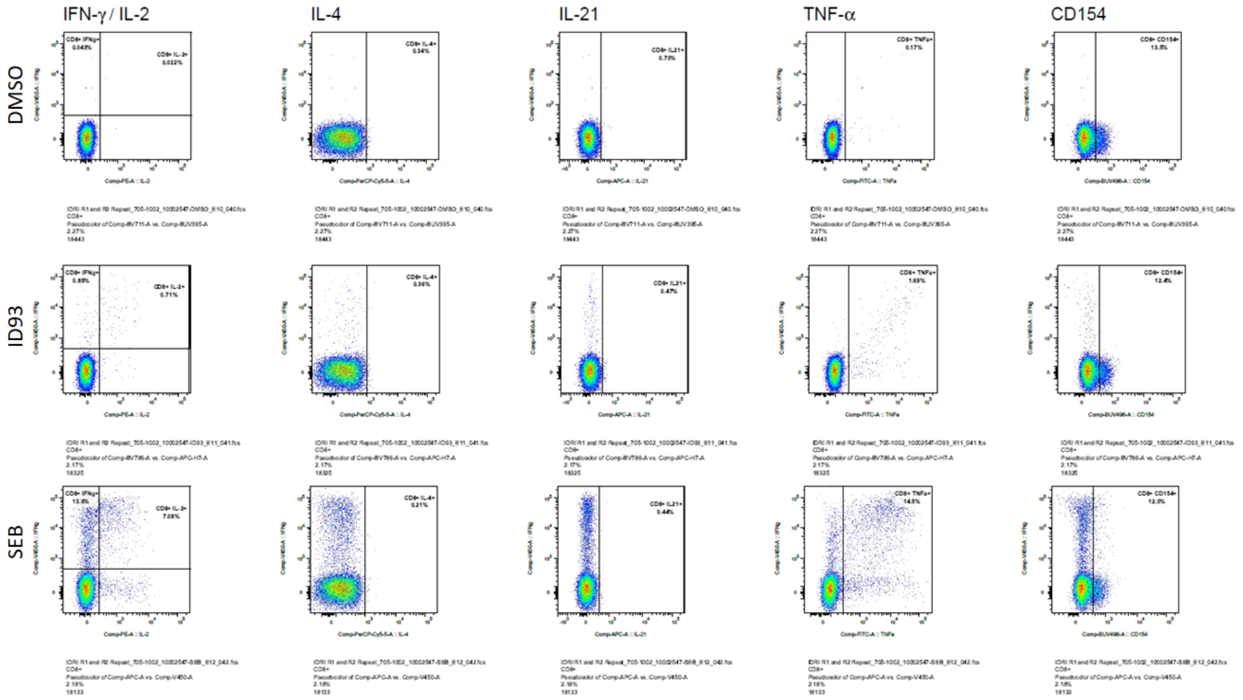

Supplementary Figure 10. Flow cytometry gating strategy and cell population percentages: cytokine expression among CD8 T cells.

4. STUDY SUBJECTS

Subject disposition, demographic, and baseline data listings are provided in Listings 16.1-16.6, and summarized in Tables 14.1.1-14.1.3.

4.1 Disposition of Subjects

This trial was conducted by investigators by Saint Louis University at a single site in Saint Louis, Missouri. Subjects were screened for inclusion in the study beginning October 29, 2018 and were enrolled to receive their first study injection starting on November 12, 2018.

Three subjects were withdrawn from the study: two were lost to follow-up prior to the Day 56 (second injection) visit and one withdrew consent prior to the Day 84 visit. One additional subject missed their second injection, however they remained on-study as they agreed to the long-term follow-up phone call on Day 421. Table 3 lists subjects who were withdrawn or had treatment discontinued.

Figure 2 is a flow chart depicting the number of individuals entering screening and the top four reasons for screen failure, and the numbers enrolling in the study and completing each study visit.

Table 3: Subjects Withdrawn from the Study or Treatment Discontinued

PID#	Last Visit Attended	Status	Reason
705-1021	Day 70	Withdrawn	Withdrew consent
705-1038	Day 14	Withdrawn	Lost to follow-up
705-1040	Day 421	Remained in follow-up; treatment discontinued	Became ineligible due to Hepatitis B vaccination
705-1048	Day 14	Withdrawn	Lost to follow-up

CONFIDENTIAL

Figure 2: Flow Diagram: IDRI-TBVPX-120

Screened but not Enrolled<br>Top Four Screen Fail Reasons		
Inclusion criterion # 03 (Screening laboratory within normal limits)	14	31.1%
Exclusion criterion # 19 (Other medical condition)	10	22.2%
Inclusion criterion # 02 (Good general health)	6	13.3%
Inclusion criterion # 07 (Compliance with protocol/procedures)	4	8.9%
TOTAL SCREEN FAILS	45	

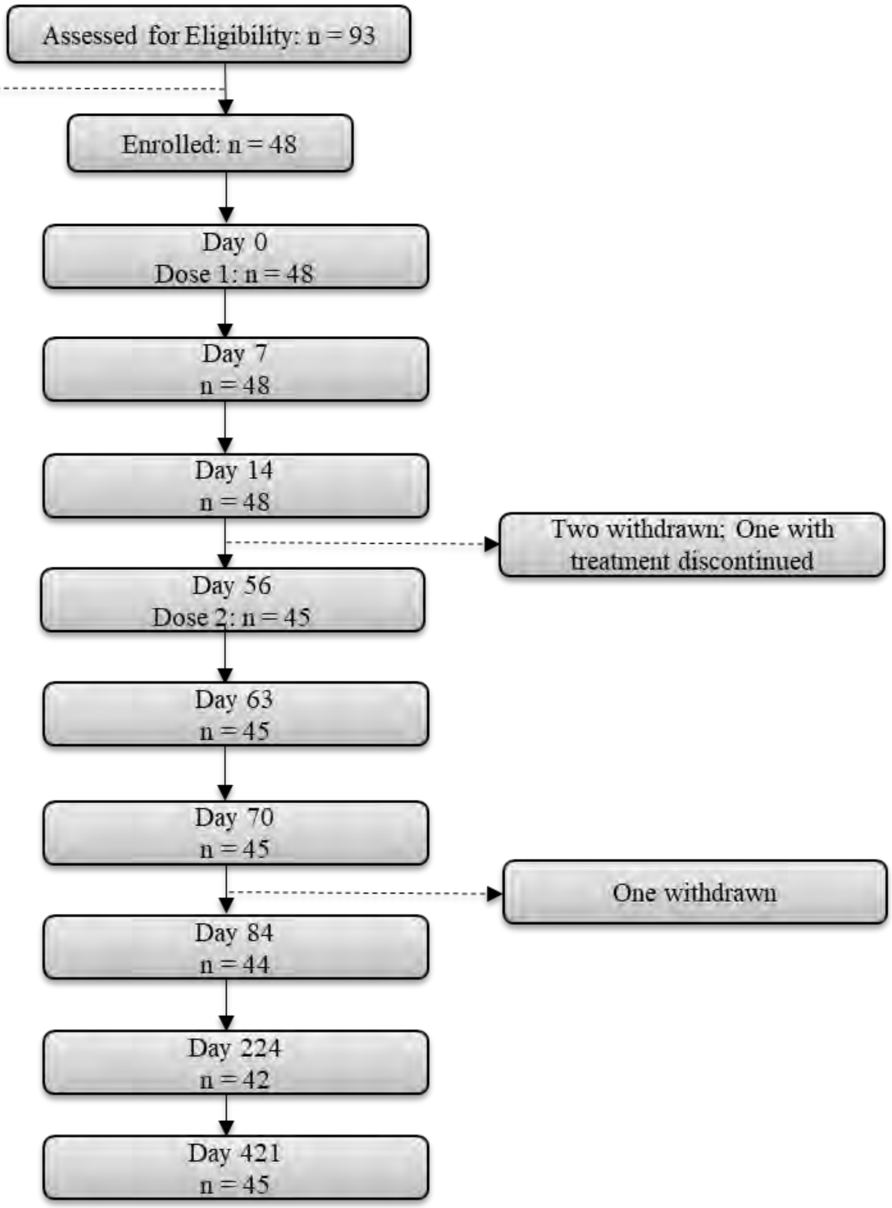

4.2 Clinical Protocol Violations

There were several protocol violations and deviations reported (Table 4): 4 for missed visits, 7 for visits performed outside the protocol allowable window, 9 for missed procedures, 8 for eligibility, and 2 classified as ‘other’ deviations. Three subjects were granted Medical Monitor approval for their second injection to occur outside the protocol window. Most missed procedures were for insufficient tear collection. The violations and deviations reported were not considered detrimental to study subjects’ safety and the integrity of study data and conclusions was not affected.

The deviations regarding the eligibility criteria for diastolic blood pressure are discussed in the next section.

Table 4: Protocol Violations and Deviations

Type of violation/deviation	PID #	Study Visit Day	Description
Missed visits	705-1006	224	Subject moved out of town
	705-1009	224	Subject moved out of town
	705-1038	56	Subject lost to follow up
	705-1048	56	Subject moved out of town
Visits out of window	705-1004	224	Visit performed 5 days out of window
	705-1012	224	Visit performed 34 days out of window
	705-1015	224	Visit performed 37 days out of window
	705-1020	56	Visit performed 6 days out of window
	705-1027	56	Visit performed 11 days out of window
	705-1045	56	Visit performed 11 days out of window
	705-1047	224	Visit performed 8 days out of window
Missed procedure	705-1003	224	Insufficient tear collection
	705-1011	0	Insufficient tear collection
	705-1012	70	PBMC blood not obtained
		63	Sodium and potassium not ordered
		224	PBMC and MGIA blood not obtained
	705-1021	0	Insufficient tear collection
		70	Subject refused tear collection
	705-1028	70	Insufficient tear collection
		224	Insufficient tear collection
Eligibility	705-1021	56	BP 115/59. Repeat BP after vaccination 110/65. Not clinically significant. Posed no safety concerns for this healthy subject per the PI. Clinic error. Staff reinstructed on protocol BP parameters. IRB notified.
	705-1029	0	BP 104/49. Repeat BP after vaccination 96/52 and 116/63. Not clinically significant. Posed no safety concerns for this healthy subject per the PI. Clinic error. Staff reinstructed on protocol BP parameters. IRB notified.
		56	BP 118/58. Repeat BP after vaccination 109/56. Follow up BP 110/67. Not clinically significant. Posed no safety concerns for this healthy subject per the PI. Clinic error. Staff reinstructed on protocol BP parameters. IRB notified.
	705-1037	SCR	BP 130/59 at Screening but normal on Day 0 prior to injection.
	705-1042	SCR	BP 128/57. Not clinically significant. Posed no safety concerns for this healthy subject per the PI. Clinic error. Staff reinstructed on protocol BP parameters. IRB notified.
		0	BP 126/59. Repeat BP after vaccination 119/60. Not clinically significant. Posed no safety concerns for this healthy subject per the PI. Clinic error. Staff reinstructed on protocol BP parameters. IRB notified.

Type of violation/deviation	PID #	Study Visit Day	Description
	705-1043	SCR	BP 107/59. Not clinically significant. Posed no safety concerns for this healthy subject per the PI. Clinic error. Staff reinstructed on protocol BP parameters. IRB notified.
		0	BP 114/59. Repeat BP after vaccination 105/70. Not clinically significant. Posed no safety concerns for this healthy subject per the PI. Clinic error. Staff reinstructed on protocol BP parameters. IRB notified.
Other deviations	705-1019	224	Incorrect green tubes used for MGIA collection. Subject returned within protocol window for visit 224 and had blood collected for MGIA in the correct 4 mL green top tubes.
	705-1022	84	Serum collection for IgG missed at Day 84. Clinic error. Subject agreed to return at later day for blood sample. Unscheduled visit still within protocol window for day 84.

4.2.1 Exemptions from Inclusion Criteria for Diastolic Blood Pressure

During the screening period (screening visit and/or Day 0 visit prior to the first injection), three subjects were found to have mild low diastolic blood pressure (57 to 59 mmHg). Similar low diastolic blood pressure was reported for one subject on the Day 0 (49 mmHg) and the Day 56 visits (58 mmHg) prior to injections and for one subject at the Day 56 visit prior to the second injection (59 mmHg). Per inclusion criterion #2, diastolic blood pressure must be ≥ 60 mmHg. Due to clinical error, these subjects were allowed to receive the study injection. Blood pressure was measured after the study injection and values ≥ 60 mmHg were observed. The PI judged these observations to be not clinically significant (NCS). The IDRI Medical Monitor agreed with the PI assessment that the deviations were not a safety concern. Table 5 lists the low diastolic blood pressure values deemed to be not clinically significant by the PI and Medical Monitor.

No other exemptions from inclusion or exclusion criteria were granted.

Table 5: NCS Diastolic Blood Pressure Values

PID #	Study Visit	Blood Pressure (mmHg) Prior to injection	Blood Pressure (mmHg) After injection
705-1021	Day 56	115/59	110/65
705-1029	Day 0<br>Day 56	104/49<br>118/58	116/63<br>109/56
705-1037	Screening	130/59	N/A
705-1042	Screening<br>Day 0	128/57<br>126/59	N/A<br>119/60
705-1043	Screening<br>Day 0	107/59<br>114/59	N/A<br>105/70

A Phase 1, Double-Blind, Randomized Clinical Trial to Evaluate the Safety, Tolerability, and Immunogenicity of the Single-Vial Lyophilized ID93 + GLA-SE Vaccine Administered Intramuscularly in Healthy Adult Subjects

IDRI Protocol Number: TBVPX-120

DMID Protocol Number: 17-0104

DMID Funding Mechanism: HHSN272201400041C

IND Sponsor: Infectious Disease Research Institute (IDRI)

Principal Investigator: Daniel Hoft, MD, PhD

IDRI Medical Monitor: Stuart Kahn, MD

DMID Medical Monitor: Mohamed Elsafy, MD, MSc

DMID Medical Officer: Mamodikoe Makhene, MD, MPH

Version Number: 2.0

05 October 2018

This document is proprietary and is to be used only as authorized. No part is to be reproduced, disclosed to others, or quoted without prior written authorization from IDRI.

STATEMENT OF COMPLIANCE

This trial will be conducted in compliance with the protocol, International Conference on Harmonisation (ICH) Guideline E6: Good Clinical Practice: Consolidated Guideline, (R2), the applicable regulatory requirements from US Code of Federal Regulations (CFR) (Title 45 CFR Part 46 and Title 21 CFR including Parts 50 and 56, 21 CFR Part 11, and 21 CFR Part 312) concerning informed consent and Institutional Review Board regulations, and the NIAID Clinical Terms of Award.

All individuals responsible for the design and conduct of this study have completed Human Subjects Protection Training and are qualified to be conducting this research prior to the enrollment of any subjects. Curricula vitae for all investigators and sub-investigators participating in this trial are on file (21 CFR 312.23 [a] [6] [iii] [b] edition).

This document is proprietary and is to be used only as authorized. No part is to be reproduced, disclosed to others, or quoted without prior written authorization from IDRI.

SIGNATURE PAGE

The signature below constitutes the approval of this protocol and the attachments, and provides the necessary assurances that this trial will be conducted according to all stipulations of the protocol, including all statements regarding confidentiality, and according to local legal and regulatory requirements and applicable US federal regulations and ICH guidelines.

Site Investigator Signature:

Signed: ______________________________ Date: ______________

Daniel Hoft, MD, PhD
Principal Investigator

Sponsor Signature:

Signed: ______________________________ Date: ______________

Corey Casper, MD, MPH
Chief Scientific Officer

This document is proprietary and is to be used only as authorized. No part is to be reproduced, disclosed to others, or quoted without prior written authorization from IDRI.

TABLE OF CONTENTS

Page
1 Key Roles 13
2 Background Information and Scientific Rationale 15
2.1 Background Information 15
2.1.1 GLA-SE 16
2.1.2 Preclinical animal studies with ID93 16
2.1.3 Clinical Experience with ID93 + GLA-SE 16
2.2 Rationale 18
2.3 Potential Risks and Benefits 18
2.3.1 Potential Risks 18
2.3.2 Known Potential Benefits 20
3 Objectives 21
3.1 Study Objectives 21
3.2 Study Outcome Measures 21
3.2.1 Primary Outcome Measures 21
3.2.2 Secondary Outcome Measures 22
3.2.3 Exploratory Outcome Measures 22
4 Study Design 23
5 Study Enrollment and Withdrawal 24
5.1 Subject Inclusion Criteria 24
5.2 Subject Exclusion Criteria 25
5.3 Eligibility Criteria for 2nd Injection 27
5.4 Treatment Assignment Procedures 27
5.4.1 Randomization Procedures 27
5.4.2 Blinding Procedures 28
5.4.3 Reasons for Withdrawal 28
5.4.4 Handling of Withdrawals 29
5.4.5 Handling of Subjects Withdrawn from Further Study Injections 29
5.4.6 Termination of Study 30
6 Study Intervention/Investigational Product 31
6.1 Study Product Description 31
6.1.1 Acquisition 31
6.1.2 Formulation, Storage, Packaging, and Labeling 31
6.2 Dosage, Preparation, and Administration of Study Intervention/Investigational Product 32
6.3 Modification of Study Intervention/Investigational Product for a Participant 33
6.4 Accountability Procedures for the Study Intervention/Investigational Product(s) 33
6.5 Concomitant Medications/Treatments 33
7 Study Schedule 35
7.1 Screening Procedures (Day -30 to Day -1) 35
7.2 Day 0 Enrollment/Baseline Evaluations and First Study Injection 35
7.3 Day 7 (±1) Evaluations 36
7.4 Day 14 (±3) Evaluations 36
7.5 Day 56 (±3) Evaluations and Second Study Injection 36
7.6 Day 63 (±1) Evaluations 37
7.7 Day 70 (±3) Evaluations 37
7.8 Day 84 (±5) Evaluation 38
7.9 Day 224 (±10) Evaluations 38

This document is proprietary and is to be used only as authorized. No part is to be reproduced, disclosed to others, or quoted without prior written authorization from IDRI.

7.10 Day 421 (±14) Evaluations 38
7.11 Early Termination Visit 38
7.12 Unscheduled Visit 39
7.13 Visit Schedule to be followed if 2nd study injection is withheld 39
8 Study Procedures AND Evaluations 40
8.1 Clinical Evaluations 40
8.2 Laboratory Evaluations 41
8.2.1 Clinical Laboratory Evaluations 41
8.2.2 Special Assays or Procedures 41
8.2.3 Specimen Shipment 42
9 Assessment of Safety 43
9.1 Specification of Safety Parameters 43
9.2 Methods and Timing for Assessing, Recording, and Analyzing Safety Parameters 43
9.2.1 Adverse Events 43
9.2.2 Assessment of Severity (Grading) 45
9.2.3 Assessment of Causality (Relatedness to Study Injection) 46
9.2.4 Evaluation of Expectedness 47
9.2.5 Reactogenicity 48
9.2.6 Memory Aids 48
9.3 Serious Adverse Events (SAE) 49
9.3.1 Assessment of Seriousness 49
9.3.2 Procedures to be Followed in the Event of Abnormal Laboratory Test Values or Abnormal Clinical Findings 50
9.4 Reporting Procedures 50
9.4.1 Serious Adverse Events 50
9.4.2 Regulatory Reporting 52
9.4.3 Reporting Other Adverse Events (if applicable) 52
9.4.4 Reporting of Pregnancy 52
9.5 Type and Duration of Follow-up of Subjects after Adverse Events 52
9.6 Halting Rules 53
9.6.1 Rules for Discontinuing Study injection in an Individual Subject 53
9.6.2 Rules for Suspension of the Entire Study 53
9.7 Safety Oversight (ISM plus SMC) 54
9.7.1 Independent Safety Monitor (ISM) 54
9.7.2 Safety Monitoring Committee (SMC) 54
10 Clinical Monitoring 56
10.1 Site Monitoring Plan 56
11 Statistical Considerations 57
11.1 Introduction 57
11.2 Study Population 57
11.2.1 Intent to Treat Population 57
11.2.2 Per Protocol Population 57
11.2.3 Safety Population 57
11.2.4 Immunogenicity Population 57
11.3 Sample Size Considerations 58
11.4 Planned Interim Analyses (if applicable) 58
11.5 Final Analysis Plan 58
11.5.1 Analysis of Demographics and Disposition 58
11.5.2 Analysis of Safety 59
11.5.3 Immunology Analyses 60
12 Source Documents and Access to Source Data/Documents 61

This document is proprietary and is to be used only as authorized. No part is to be reproduced, disclosed to others, or quoted without prior written authorization from IDRI.

13 Quality Control and Quality Assurance 62
14 Ethics/Protection of Human Subjects 63
14.1 Ethical Standard 63
14.2 Institutional Review Board 63
14.3 Informed Consent Process 63
14.4 Exclusion of Women, Minorities, and Children (Special Populations) 64
14.5 Subject Confidentiality 64
14.6 Study Discontinuation 65
14.7 Future Use of Stored Specimens 65
14.8 Disclosure of Individual Research Information 66
15 Data Handling and Record Keeping 67
15.1 Data Management Responsibilities 67
15.2 Data Capture Methods 67
15.3 Types of Data 67
15.4 Timing/Reports 67
15.5 Study Records Retention 68
15.6 Protocol Deviations 68
16 Publication Policy 69
17 Literature References 71
Appendix 1: Schedule of Study Visits and Procedures 72
Appendix 2: Schedule of Blood Draws and Volumes 73
Appendix 3: Grading Scale for Clinical Laboratory Values 74
Appendix 4: Grading Scale for Local Injection Site Reactions 75
Appendix 5: Grading Scale for Determining the Severity of Clinical AEs 76
Appendix 6: List of PIMMCs 77

This document is proprietary and is to be used only as authorized. No part is to be reproduced, disclosed to others, or quoted without prior written authorization from IDRI.

LIST OF ABBREVIATIONS

AE	Adverse Event/Adverse Experience
ALT	Alanine Aminotransferase
AST	Aspartate Aminotransferase
BCG	Bacille Calmette-Guérin (Vaccine)
BP	Blood Pressure
CBER	Center for Biologics Evaluation and Research, FDA, DHHS
CFR	Code of Federal Regulations
CROMS	Clinical Research Operations and Management Support
CRP	C-Reactive Protein
DHHS	Department of Health and Human Services
DMID	Division of Microbiology and Infectious Diseases, NIAID, NIH, DHHS
eCRF	Electronic Case Report Form
ELISA	Enzyme linked immunosorbent assay
ELISpot	Enzyme linked immunospot assay
ER	Emergency room
ET	Early Termination
FDA	Food and Drug Administration
GCP	Good Clinical Practice
GLA	Glucopyranosyl Lipid A
GLA-SE	Glucopyranosyl Lipid A – Stable Emulsion
GLP	Good Laboratory Practice
HBsAg	Hepatitis B surface antigen
HCV	Hepatitis C Virus
HEENT	Head, Eyes, Ears, Nose, and Throat
HGB	Hemoglobin
HIV	Human Immunodeficiency Virus
ICH	International Conference on Harmonisation
ICS	Intracellular Cytokine Staining
IDRI	Infectious Diseases Research Institute
IEC	Independent or Institutional Ethics Committee
IgG	Immunoglobulin G
IM	Intramuscular
IND	Investigational New Drug Application
IFNγ	Interferon Gamma
IRB	Institutional Review Board
ISM	Independent Safety Monitor
IV	Intravenous
LLN	Lower limit of normal
Mtb	*Mycobacterium tuberculosis*
MPL	Monophosphoryl Lipid A
N	Number (typically refers to subjects)

This document is proprietary and is to be used only as authorized. No part is to be reproduced, disclosed to others, or quoted without prior written authorization from IDRI.

NIAID	National Institute of Allergy and Infectious Diseases, NIH, DHHS
NIH	National Institutes of Health
P	Pulse
PBMC	Peripheral Blood Mononuclear Cell
PE	Physical exam
PHI	Protected Health Information
PI	Principal Investigator
PID	Participant identification number
PIMMC	Potential immune-mediated medical condition
RBC	Red Blood Cell
RR	Respiratory Rate
SAE	Serious Adverse Event/Serious Adverse Experience
SMC	Safety Monitoring Committee
SSP	Study Specific Procedures
SUSAR	Suspected Unexpected Serious Adverse Reaction
TB	Tuberculosis
TLR	Toll-like receptor
TNF	Tumor Necrosis Factor Alpha
ULN	Upper limit of normal
US	United States
USP	Unites States Pharmacopeia
WBC	White Blood Cell Count

This document is proprietary and is to be used only as authorized. No part is to be reproduced, disclosed to others, or quoted without prior written authorization from IDRI.

PROTOCOL SUMMARY

**Title:**	**A Phase 1, Double-Blind, Randomized Clinical Trial to Evaluate the Safety, Tolerability, and Immunogenicity of the Single-Vial Lyophilized ID93 + GLA-SE Vaccine Administered Intramuscularly in Healthy Adult Subjects**
**Phase:**	1
**Population:**	Healthy males and non-pregnant females, aged 18-55. A total of 48 subjects will be enrolled.
**Number of Sites:**	One site: Saint Louis University
**Study Duration:**	~18 months
**Subject Participation Duration:**	~14 months
**Estimated Time to Complete Enrollment:**	Approximately 4 months
**Description of Agent or Intervention:**	• ID93 (2 µg, lyophilized) + GLA-SE (5 µg, liquid) [two-vial presentation]<br>• ID93 + GLA-SE (2 µg ID93, 5 µg GLA) [lyophilized, single-vial]
**Study Objectives:**	Primary:<br>• To evaluate the safety and tolerability of single-vial lyophilized ID93 + GLA-SE following two intramuscular (IM) injections administered on Days 0 and 56.<br>• To compare the safety and tolerability of single-vial lyophilized ID93 + GLA-SE to the two-vial presentation consisting of lyophilized ID93 and liquid GLA-SE following two IM injections administered on Days 0 and 56.<br><br>Secondary:<br>• To assess the immunogenicity of single-vial lyophilized ID93 + GLA-SE following two IM injections administered on Days 0 and 56 by quantifying cytokine production and IgG antibody responses to ID93 at specified time points.<br>• To compare the immunogenicity of single-vial lyophilized ID93 + GLA-SE to the two-vial presentation consisting of lyophilized

This document is proprietary and is to be used only as authorized. No part is to be reproduced, disclosed to others, or quoted without prior written authorization from IDRI.

	ID93 and liquid GLA-SE following two IM injections administered on Days 0 and 56.<br><br>Exploratory:<br>• To further evaluate the immunogenicity of ID93 + GLA-SE following two IM injections administered on Days 0 and 56 by measuring mycobacterial growth inhibition, quantifying IgA levels in mucosal secretions, and measuring frequencies of circulating T follicular helper cells, antibody secreting cells, and memory B cells at specified time points.
**Study Outcome Measures:**	Primary:<br>• The number of subjects experiencing solicited local injection site reactions within 7 days following each study injection.<br>• The number of subjects experiencing solicited systemic reactions within 7 days following each study injection.<br>• The number of subjects spontaneously reporting adverse events from Day 0 through Day 84.<br>• The number of serious adverse events considered related to any of the study injections reported at any point during the study period.<br><br>Secondary:<br>• The proportion of subjects with at least a 4-fold increase in IgG antibody responses to ID93 on Days 14, 56, 70, 84, and 224 relative to baseline (Day 0).<br>• Mean fold change from baseline (Day 0) in IgG antibody responses to ID93 on Days 14, 56, 70, 84, and 224.<br>• The number of IFN-γ and IL-10 cytokine-secreting cells in PBMC samples in response to the ID93 antigen relative to baseline (Day 0) at Days 14, 56, 70, 84, and 224, as assayed by ELISpot<br>• Percentage of CD4 and CD8 T cells producing 1 or more cytokines (IFN-γ, TNF, and IL-2) simultaneously in response to stimulation with the ID93 antigen as measured by intracellular cytokine staining of PBMCs at Days 14, 56, 70, 84, and 224 relative to baseline (Day 0).

This document is proprietary and is to be used only as authorized. No part is to be reproduced, disclosed to others, or quoted without prior written authorization from IDRI.

<table>
<tr><td></td><td>Exploratory:
• Measurement of net intracellular growth inhibition of Mtb using whole blood on Days 0, 70, and 224.
• Quantitate IgA titers in mucosal secretions (nasal swabs and tear collections) by ELISA on Days 0, 70, and 224.
• Quantitate number of antibody-secreting cells in PBMC by short-culture B cell ELISpot on Days 0, 7, and 63.
• Quantitate number of antigen-specific memory B cells in PBMC by long-culture B cell ELISpot on Days 0, 56, 84, and 224.
• Quantitate T follicular helper cells, memory B cells, and T cell homing markers in PBMC by immunophenotyping flow cytometry on Days 0, 7, 14, 63, 70, and 224.</td></tr>
<tr><td>Description of Study Design:</td><td>This is a phase 1, double-blind, randomized clinical trial to evaluate the safety, tolerability, and immunogenicity of single-vial lyophilized ID93 + GLA-SE compared to the two-vial presentation consisting of lyophilized ID93 and liquid GLA-SE administered as two IM injections in healthy adult subjects (aged 18 - 55).

The two treatment groups are outlined in the table below. Subjects will receive a total of two doses administered IM on Days 0 and 56. Subjects will be monitored for approximately 421 days (one year following the last study injection), including safety laboratory analyses done just prior to and 7 days following each study injection. Tears and nasal swabs will be obtained for exploratory antibody analysis at Days 0, 70, and 224. Blood samples will be obtained for immunological assays (secondary and exploratory) at Days 0, 7, 14, 56, 63, 70, 84, and 224).

<table>
<tr><th>Group</th><th>N</th><th>Study Injections</th><th>Timing of Study Injections</th></tr>
<tr><td>1</td><td>24</td><td>ID93 (2 µg, lyophilized) +
GLA-SE (5 µg, liquid)
[two-vial presentation]</td><td>Days 0, 56</td></tr>
<tr><td>2</td><td>24</td><td>ID93 + GLA-SE
(2 µg ID93, 5 µg GLA)
[lyophilized, single-vial]</td><td>Days 0, 56</td></tr>
</table>
</td></tr>
</table>

This document is proprietary and is to be used only as authorized. No part is to be reproduced, disclosed to others, or quoted without prior written authorization from IDRI.

SCHEMATIC OF STUDY DESIGN

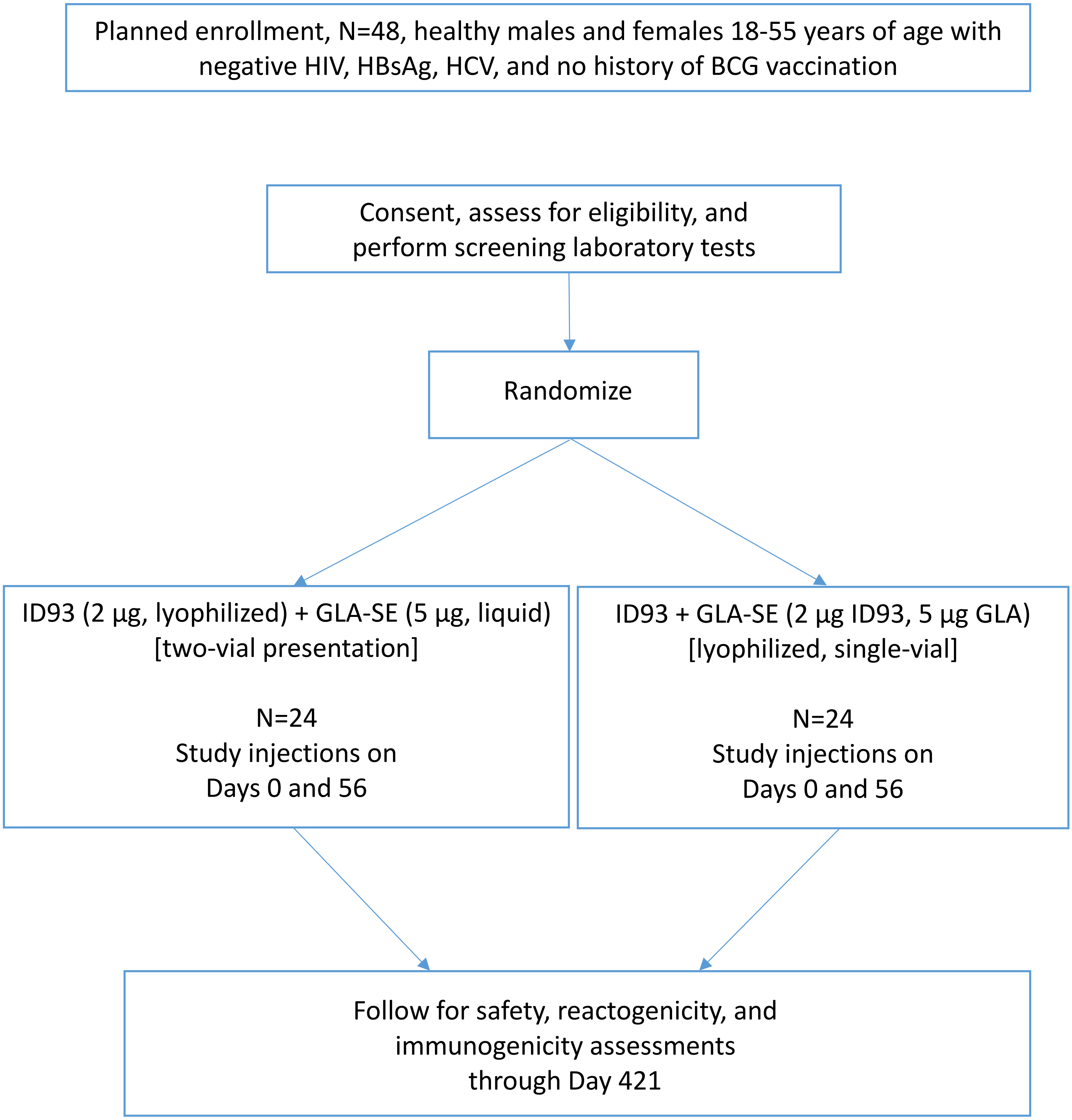

This document is proprietary and is to be used only as authorized. No part is to be reproduced, disclosed to others, or quoted without prior written authorization from IDRI.

1 KEY ROLES

**Principal Investigator:**	Daniel Hoft, MD, PhD<br>Saint Louis University<br>Center for Vaccine Development<br>1100 S. Grand Blvd., 8th Floor<br>St. Louis, MO 63104<br>Phone: 314-977-9044<br>Fax: 314-771-3816<br>E-mail: Daniel.Hoft@health.slu.edu
**Sponsor:**	Infectious Disease Research Institute (IDRI)<br>1616 Eastlake Avenue East, Suite 400<br>Seattle, WA 98102<br>Phone: 206-381-0883<br>Fax: 206-858-6098<br><br>NIAID Contract Principal Investigator:<br>Chris Fox, PhD<br>VP, Formulations<br>Phone: 206-858-6027<br>E-mail: Chris.Fox@idri.org<br><br>Anna Marie Beckmann, PhD<br>SVP, Product Development and Regulatory Affairs<br>Phone: 206-858-6099<br>E-mail: AnnaMarie.Beckmann@idri.org<br><br>Corey Casper, MD, MPH<br>Chief Scientific Officer<br>Phone: 206-858-6054<br>E-mail: Corey.Casper@idri.org<br><br>Aude Frevol, RN, PMP<br>Clinical Trial Director<br>Phone: 206-858-6039<br>E-mail: Aude.Frevol@idri.org<br><br>Zachary Sagawa, MS<br>Regulatory Manager<br>Phone: 206-858-6040<br>E-mail: Zachary.Sagawa@idri.org<br><br>Rhea Coler, PhD<br>SVP, Pre-Clinical and Translational Research<br>Phone: 206-858-6065<br>E-mail: Rhea.Coler@idri.org

This document is proprietary and is to be used only as authorized. No part is to be reproduced, disclosed to others, or quoted without prior written authorization from IDRI.

	Tracey Day, PhD<br>Clinical Immunologist<br>Phone: 206-858-6044<br>E-mail: Tracey.Day@idri.org
**IDRI Medical Monitor**	Stuart Kahn, MD<br>Phone: 206-330-7604<br>E-mail: stujkahn@outlook.com
**DMID Medical Monitor**	Mohamed Elsafy, MD, MSc<br>OCRA/DMID/NIAID/NIH<br>5601 Fishers Lane, 7E56<br>Rockville, MD 20852 USA<br>Tel: +1 (240) 292 4241<br>Mobile: +1 (301) 204 3735<br>Email: mo.elsafy@nih.gov
**DMID Medical Officer**	Mamodikoe Makhene, MD, MPH<br>RDB/DMID/NIAID/NIH<br>5601 Fishers Lane, 8E24<br>Rockville, MD 20852<br>Tel: 240-627-3309<br>Email: mmakhene@niaid.nih.gov
**Statistics and Data Management**	DF/Net Research, Inc.<br>Lisa Ondrejcek<br>President<br>140 Lakeside Avenue, Suite 310<br>Seattle, WA 98122<br>Phone: 206-322-5931<br>Fax: 206-686-5119<br>Email: lisa@dfnetresearch.com
**Clinical Site**	Saint Louis University<br>Center for Vaccine Development<br>1100 S. Grand Blvd., 8th Floor<br>St. Louis, MO 63104
**Clinical Safety Laboratory**	Lab One, LLC Quest Diagnostic<br>10101 Renner Blvd<br>Lenexa, KS 66219-9752
**Immunology Laboratory**	IDRI<br>1616 Eastlake Avenue East, Suite 400<br>Seattle, WA 98102

This document is proprietary and is to be used only as authorized. No part is to be reproduced, disclosed to others, or quoted without prior written authorization from IDRI.

2 BACKGROUND INFORMATION AND SCIENTIFIC RATIONALE

2.1 Background Information

Tuberculosis (TB) is an infectious disease caused by the bacterium *Mycobacterium tuberculosis* (Mtb). TB is one of the leading causes of morbidity and mortality around the world with an estimated 8 million cases of active TB each year and 1.3 million deaths annually (http://www.who.int/mediacentre/factsheets/fs104/en/). Though TB is often curable, treatment requires prolonged multidrug therapy (6 or more months). Compliance is difficult in all patient populations, but it is particularly difficult in resource-limited countries where the majority of cases of active TB are identified. Therapy has become more complex since it is increasingly common to find strains of Mtb that have developed resistance to many of the standard antimycobacterial drugs. Extensively drug resistant strains that are resistant to first and second line antitubercular agents have been identified around the world.

Given the widespread prevalence of TB, the high rates of morbidity and mortality, and the complexities of treatment, effective TB vaccines could have profound impacts on global health. However, it is critical to realize that the nature of protective immunity for Mtb is complex and immune correlates of protection remain unknown [1]. Animal and human studies have demonstrated that a robust T cell-mediated immune response with active engagement of Th1 CD4 and CD8 cells is critical for control of Mtb. In particular, interferon gamma (IFNγ) and tumor necrosis factor (TNF) producing CD4 T cells are critical [2-5].

The only licensed TB vaccine is Bacille Calmette-Guérin (BCG), a live attenuated strain of *Mycobacterium bovis*. BCG has been extensively studied around the world. It is effective in preventing TB meningitis and disseminated TB in children living in high prevalence areas of the world, but has had little effect on preventing reinfection and active pulmonary disease [6-8]. Today, BCG is often administered to infants and young children residing in many TB endemic countries, but as TB remains the leading infectious disease killer in the word, there is a clear need for more effective vaccines that better prevent infection, reactivation and reinfection.

A number of Mtb vaccine candidates are under development and have entered clinical trials (Reviewed in [1, 9-11]). Vaccines can be grouped based on their target population as therapeutic or preventative and preventative vaccines can be used either pre-exposure or post-exposure. Post-exposure vaccines are intended to prevent development of active disease in already exposed and/or latently infected individuals. Though some of the vaccine candidates are being designed to replace BCG, others incorporate BCG into their proposed vaccine schema and would serve as a booster vaccine to be administered either soon after BCG administration in infants or later in life to boost waning BCG-responses [11]. ID93 is a recombinant subunit vaccine antigen that combines four antigens comprised of Mtb proteins that are associated with virulence and latency into a single recombinant protein [12]. The antigenicity of ID93 was tested by ex vivo stimulation of human leukocytes isolated from humans who were reactive to Mtb purified protein derivative (PPD+). ID93 stimulation elicited CD4 and CD8 T cells expressing one or more of the cytokines, IFNγ, TNF, or IL-2. Various adjuvants and adjuvant formulations were explored to

This document is proprietary and is to be used only as authorized. No part is to be reproduced, disclosed to others, or quoted without prior written authorization from IDRI.

enhance the Th1 response [13]. Mice, guinea pigs, and cynomolgus monkeys immunized with ID93 + GLA-SE showed reduced pathology and/or fewer bacilli after challenge with virulent Mtb [12].

This trial will evaluate ID93 + GLA-SE lyophilized in a single vial to produce a thermostable candidate vaccine. Previous clinical trials have used a two-vial presentation, which in this trial serves as a comparator.

2.1.1 GLA-SE

Glucopyranosyl Lipid A (GLA) is a synthetic Toll-like Receptor 4 (TLR4) agonist and is an analog of the well-studied adjuvant Monophosphoryl Lipid A (MPL®; GlaxoSmithKline Biologicals). GLA is formulated in a stable oil-in-water emulsion (SE) to yield the adjuvant formulation GLA-SE. Due to the TLR4 activity of the GLA molecule, the combination of GLA-SE with a recombinant protein antigen (ID93) results in a Th1-type T cell response.

2.1.2 Preclinical animal studies with ID93

Multiple preclinical studies in human cells, mice, guinea pigs, non-human primates, and GLP toxicity studies in rabbits have demonstrated the safety and immunogenicity of ID93 + GLA-SE. Mice, guinea pigs, and non-human primates have shown that ID93 + GLA-SE is well-tolerated and immunogenic. Upon challenge with Mtb, animals showed significantly lower bacterial burdens in lungs and spleens compared with animals injected with saline, antigen alone, or adjuvant alone (see Investigator's Brochure). Cells isolated from lung infiltrates showed that there was an increased proportion of ID93-specific CD4 T cells that expressed both IFNγ and TNF compared to controls. Primate studies showed that animals developed a mild acute phase response following each injection with mild-moderate increases in neutrophils, white blood cells, fibrinogen, and C-reactive protein (CRP). Increases in neutrophils and WBC were similar following repeated doses, though there was a mild increase in the magnitude of fibrinogen and CRP with each dose. Findings resolved within 2 weeks.

Murine studies showed that ID93 + GLA-SE enhanced the Th1 response and elicited specific splenic CD4 T cells producing IFNγ, TNF, and IL-2. Similar reductions in bacterial burden were seen in the lungs of Mtb challenged mice.

A repeated-dose toxicity study was performed in New Zealand white rabbits evaluating four weekly intramuscular doses of ID93 + GLA-SE. The vaccine candidate was well tolerated and immunogenic. As with the primate studies, mild increases in WBC, fibrinogen, and globulin were seen.

2.1.3 Clinical Experience with ID93 + GLA-SE

This trial will be the first in which the lyophilized, single-vial presentation of ID93 + GLA-SE is tested. ID93 + GLA-SE in the two-vial presentation has been evaluated in four completed Phase 1 and 2 clinical trials.

This document is proprietary and is to be used only as authorized. No part is to be reproduced, disclosed to others, or quoted without prior written authorization from IDRI.

The first trial, TBVPX-113, was a phase 1, dose-escalation study evaluating 60 healthy adults randomized into eight different groups that explored two different dose levels of ID93 antigen (2 or 10 µg), two different dose levels of the GLA-SE adjuvant (2 or 5 µg), or ID93 antigen alone (2 or 10 µg). Each subject received three IM injections on Days 0, 28, and 56. The vaccine was safe and well-tolerated at all dose levels. Adverse reactions included injection site reactions and mild-moderate systemic symptoms. There were no clinically significant changes in clinical chemistry or hematology values related to the study product. The mild changes in sodium and potassium that are outlined in the Investigator's Brochure were determined to be due to normal values for the particular laboratory that overlapped with the FDA toxicity tables and to delayed laboratory processing of blood samples. ID93 + GLA-SE elicited a robust ID93-specific antibody response and a polyfunctional CD4 T cell response, whereas responses to ID93 alone were significantly lower.

The second study, TBVPX-114, was a phase 1b, randomized, double-blind, placebo-controlled, dose-escalation evaluation of two dose levels of the ID93 antigen (2 or 10 µg) administered in combination with two dose levels of the GLA-SE adjuvant (2 or 5 µg). Each subject received three IM injections on Days 0, 28, and 112. The study was conducted in South Africa and enrolled 66 healthy adults previously vaccinated with BCG. Some groups included individuals with a positive QuantiFERON®-TB Gold test (i.e., with a history of exposure to TB). The vaccine was safe and well-tolerated in this endemic population with a similar safety profile to TBVPX-113. Adverse reactions mainly consisted of local and systemic reactogenicity, including mild to moderate injection site pain, injection site swelling, myalgia, headache, and chills. The vaccine elicited ID93-specific CD4 T cell and antibody responses in all groups. Higher responses were observed in QuantiFERON-positive than in QuantiFERON-negative individuals.

The third phase 1 trial, DMID 12-0109, was a randomized and double-blind clinical trial to evaluate the safety, tolerability, and immunogenicity of ID93 (10 µg) alone or in combination with AP10-602 (5 or 10 µg) or GLA-SE (5 µg). AP 10-602 is another GLA-based adjuvant in a liposomal formulation with the saponin QS-21. Each subject received three IM injections on Days 0, 28, and 56. A total of 70 participants were enrolled and the study is complete. The database was recently locked and the clinical study report is in preparation. Unblinded results are not yet available; however, no significant safety issues were identified.

The phase 2a study, TBVPX-203, was a randomized, double-blind, placebo-controlled, dose-escalation evaluation of two dose levels of the ID93 antigen (2 or 10 µg) administered IM in combination with two dose levels of the GLA-SE adjuvant (2 or 5 µg). The study also compared a two-dose regimen (Days 0, 56) with the three-dose regimen (Days 0, 28, 56) studied in the phase 1 trials. This study was conducted in 60 South African adults following successful completion of standard treatment and cure for Mtb culture-confirmed, drug-susceptible pulmonary TB. This trial was a lead-in to a future larger phase 2b trial intended to test efficacy for reducing the rate of disease relapse in people recently cured of TB. The vaccine was safe and well-tolerated in this population.

Numerous studies have evaluated GLA-SE in combination with a wide variety of other infectious disease antigens, with no significant safety issues identified. In addition, two studies evaluated the safety and tolerability of two dose levels of GLA-SE given alone without antigen by the IM or

This document is proprietary and is to be used only as authorized. No part is to be reproduced, disclosed to others, or quoted without prior written authorization from IDRI.

SC route. GLA-SE was safe and well-tolerated. The most common adverse events (AEs) were injection site reactions and systemic reactions including headache, fatigue, myalgia, malaise, and dizziness. Most AEs were graded as mild. There were no clinically significant changes in clinical chemistry or hematology values.

2.2 Rationale

TB causes significant morbidity and mortality throughout the world. Strains of Mtb are becoming increasingly resistant to antimycobacterial drugs and therapy is often associated with poor compliance, which increases the likelihood of antimicrobial resistance. The current licensed BCG vaccine does not prevent reactivation or reinfection in adults so there is a great need for a more effective vaccine. ID93 is a recombinant antigen that, when mixed with the adjuvant GLA-SE, shows great promise in eliciting what is thought to be an important Th1 T cell response and limits the bacterial burden in Mtb challenged animals. Thermostable vaccines facilitate greater distribution capacity in the developing world and reduce vaccine wastage. Development of a vaccine against Mtb that does not require cold-chain maintenance would significantly lower distribution cost and enable more effective delivery in low resource settings.

This phase 1, double-blind, randomized clinical trial will evaluate the safety, tolerability, and immunogenicity of ID93 + GLA-SE in the lyophilized, single-vial presentation. The goal of this study is to evaluate the safety and tolerability of the new lyophilized single-vial presentation ID93 + GLA-SE and how immunological responses elicited by this product compare to ID93 + GLA-SE prepared from the two-vial presentation. One dose level of ID93 + GLA-SE will be tested (2 µg of ID93 and 5 µg of GLA-SE). This dose was selected because safety and immunogenicity results from previous clinical trials have indicated this may be the optimal dose. This trial is a bridging study that will allow the thermostable single-vial vaccine to move forward in clinical development.

2.3 Potential Risks and Benefits

2.3.1 Potential Risks

Safety data from the TBVPX-113, -114, and -203 trials of ID93 + GLA-SE are summarized here.

The vaccine (combined ID93 + GLA-SE) was safe and well-tolerated:

- The most common adverse events were injection site reactions (pain/tenderness, erythema, and induration) and certain injection-associated systemic reactions (headache, fatigue, myalgia, anorexia, chills, and arthralgia). The majority of adverse events were graded as mild (Grade 1).
- There were no clinically significant changes in clinical chemistry or hematology values though there were subjects that experienced Grade 1 and Grade 2 fluctuations in clinical chemistry and hematology values (including decreased hemoglobin, neutrophils, lymphocytes, and WBC).

This document is proprietary and is to be used only as authorized. No part is to be reproduced, disclosed to others, or quoted without prior written authorization from IDRI.

These findings were typically mild and transient, typical of vaccinations by the IM route, and indicative of the expected immunomodulatory effect of GLA-SE. There were no Grade 4 AEs, nor were there any serious AEs, AEs defined as a dose-limiting toxicity, or AEs of special interest (AEs of special interest were renamed Potential Immune-Mediated Medical Conditions (PIMMCs)). No subjects withdrew from the studies because of an AE.

GLA-SE has been tested in numerous clinical trials in combination with a wide variety of antigens for infectious diseases including influenza, leishmaniasis, leprosy, schistosomiasis, and malaria. Several trials evaluated GLA-SE given alone or in combination with an antigen as a cancer immunotherapy.

These clinical trials are evaluating GLA-SE at dose levels ranging from 0.5 to 20 µg. In these studies, over 1100 subjects have received at least one study injection containing GLA-SE. Safety data have not revealed any significant safety issues at any dose level tested. These studies have revealed an acceptable safety profile:

- Study injections are generally well tolerated
- There have been no serious adverse events related to study injection
- Injection site reactions are common and may include pain, tenderness, erythema, and induration
- Systemic reactions may include headache, fatigue, anorexia, fever, chills, myalgia, arthralgia, and anorexia
- Transient elevations in CRP levels were noted in one study
- Hematologic changes may occur (including decreases in hemoglobin, WBC, lymphocytes, and neutrophils).

These reactions varied from study to study, were generally mild, resolved quickly, and are typical of vaccinations by the IM route. GLA-SE often increases the rate and severity of local and systemic reactogenicity compared to the antigen alone. This is in line with the nonclinical animal experience and is to be expected of a potent immunostimulant.

Based on the evidence provided, we expect the risks for this study to include discomfort of having blood drawn and there may be pain, soreness, redness, warmth, swelling, firmness, and itching at the site of injection. Other reactions may include fever, chills, rash, myalgia, nausea, arthralgia, headache, fatigue, malaise. As with other vaccines, an allergic reaction including anaphylaxis is possible. We expect that there may be some mild to moderate transient fluctuations in serum chemistry and hematology values.

There may be other side effects from the study injections, even serious ones that are not yet known.

It is unknown if ID93 alone or in combination with GLA-SE poses any risks to an unborn or breastfeeding child.

This document is proprietary and is to be used only as authorized. No part is to be reproduced, disclosed to others, or quoted without prior written authorization from IDRI.

Subjects will be asked to provide personal health information (PHI). All attempts will be made to keep this PHI confidential within the limits of the law. However, there is a chance that unauthorized persons will see the subjects' PHI. All records will be kept in a locked file cabinet or maintained in a locked room at the clinical site. Electronic files will be password protected. Only people who are involved in the conduct, oversight, monitoring, or auditing of this study will be allowed access to the PHI that is collected. Any publications from this study will not use information that will identify subjects by name. Organizations that may inspect and/or copy research records maintained at the clinical site for quality assurance and data analysis include groups, such as the sponsor IDRI, the National Institute of Allergy and Infectious Diseases (NIAID), and the Food and Drug Administration (FDA).

2.3.2 Known Potential Benefits

There will be no direct benefit to subjects from the experimental vaccine. Subjects who participate will contribute to knowledge about the safety and immunogenicity of this investigational vaccine that may lead to better prevention and treatment of TB for those living in parts of the world where TB is endemic. Subjects will receive a complete physical exam and routine laboratory tests to assess their health status.

Society may benefit from the potential development of a new vaccine against TB.

This document is proprietary and is to be used only as authorized. No part is to be reproduced, disclosed to others, or quoted without prior written authorization from IDRI.

3 OBJECTIVES

3.1 Study Objectives

Primary:

- To evaluate the safety and tolerability of single-vial lyophilized ID93 + GLA-SE following two intramuscular (IM) injections administered on Days 0 and 56.
- To compare the safety and tolerability of single-vial lyophilized ID93 + GLA-SE to the two-vial presentation consisting of lyophilized ID93 and liquid GLA-SE following two IM injections administered on Days 0 and 56.

Secondary:

- To assess the immunogenicity of single-vial lyophilized ID93 + GLA-SE following two IM injections administered on Days 0 and 56 by quantifying cytokine production and IgG antibody responses to ID93 at specified time points.
- To compare the immunogenicity of single-vial lyophilized ID93 + GLA-SE to the two-vial presentation consisting of lyophilized ID93 and liquid GLA-SE following two IM injections administered on Days 0 and 56.

Exploratory:

- To further evaluate the immunogenicity of ID93 + GLA-SE following two IM injections administered on Days 0 and 56 by measuring mycobacterial growth inhibition, quantifying IgA levels in mucosal secretions, and measuring frequencies of circulating T follicular helper cells, antibody secreting cells, and memory B cells at specified time points.

3.2 Study Outcome Measures

3.2.1 Primary Outcome Measures

- The number of subjects experiencing solicited local injection site reactions within 7 days following each study injection.
- The number of subjects experiencing solicited systemic reactions within 7 days following each study injection.
- The number of subjects spontaneously reporting adverse events from Day 0 through Day 84.
- The number of serious adverse events considered related to any of the study injections reported at any point during the study period.

This document is proprietary and is to be used only as authorized. No part is to be reproduced, disclosed to others, or quoted without prior written authorization from IDRI.

3.2.2 Secondary Outcome Measures

- The proportion of subjects with at least a 4-fold increase in IgG antibody responses to ID93 on Days 14, 56, 70, 84, and 224 relative to baseline (Day 0).
- Mean fold change from baseline (Day 0) in IgG antibody responses to ID93 on Days 14, 56, 70, 84, and 224.
- The number of IFN-γ and IL-10 cytokine-secreting cells in PBMC samples in response to the ID93 antigen relative to baseline (Day 0) at Days 14, 56, 70, 84, and 224, as assayed by ELISpot.
- Percentage of CD4 and CD8 T cells producing 1 or more cytokines (IFN-γ, TNF, and IL-2) simultaneously in response to stimulation with the ID93 antigen as measured by intracellular cytokine staining of PBMCs at Days 14, 56, 70, 84, and 224 relative to baseline (Day 0).

3.2.3 Exploratory Outcome Measures

- Measurement of net intracellular growth inhibition of Mtb using whole blood on Days 0, 70, and 224.
- Quantitate IgA titers in mucosal secretions (nasal swabs and tear collections) by ELISA on Days 0, 70, and 224.
- Quantitate number of antibody-secreting cells in PBMC by short-culture B cell ELISpot on Days 0, 7, and 63.
- Quantitate number of antigen-specific memory B cells in PBMC by long-culture B cell ELISpot on Days 0, 56, 84, and 224.
- Quantitate T follicular helper cells, memory B cells, and T cell homing markers in PBMC by immunophenotyping flow cytometry on Days 0, 7, 14, 63, 70, and 224.

This document is proprietary and is to be used only as authorized. No part is to be reproduced, disclosed to others, or quoted without prior written authorization from IDRI.

4 STUDY DESIGN

This study is a phase 1, randomized, double-blind clinical trial designed to evaluate the safety, tolerability, and immunogenicity of single-vial lyophilized ID93 + GLA-SE compared to the two-vial presentation consisting of lyophilized ID93 and liquid GLA-SE administered as two intramuscular injections in healthy adult subjects.

This study will enroll 48 healthy subjects aged 18 to 55. Subjects will be counseled on the study and will then give an informed consent. Screening will be performed that will include evaluation of medical history, medication history, a physical examination, and safety laboratory evaluations. Subjects who meet all eligibility criteria will be randomized 1:1 into the two treatment groups. Study injections will be performed and subjects will be observed in clinic for 60 minutes. All subjects will complete a written subject memory aid that solicits local and systemic reactogenicity adverse events for 7 days following each study injection. Subjects will undergo a second study injection on Day 56. General safety will be evaluated on Days 0, 7, 56, 63, and 84 for each subject. All data will be recorded in the source document during the study period.

Non-serious adverse events will be collected through Day 84 (28 days following the last study injection). The occurrence of serious adverse events (SAEs) and the new onset of any potential immune-mediated medical conditions (PIMMCs) will be collected throughout the study period (approximately 421 days).

The details of all study visits are outlined in Section 7. Each subject's duration of participation will be about 14 months. A detailed outline of the study visits and procedures is provided in the Study Visit Schedule (Appendix 1). Immunogenicity will be assessed on Day 0 (pre-dose) and on Days 7, 14, 56, 63, 70, 84, and 224. Blood will be assessed for safety laboratories on the day of screening, and on Days 7 and 63. Urinalysis will be performed at screening.

This document is proprietary and is to be used only as authorized. No part is to be reproduced, disclosed to others, or quoted without prior written authorization from IDRI.

5 STUDY ENROLLMENT AND WITHDRAWAL

Forty-eight (48) subjects between the ages of 18 and 55 years will be enrolled over an estimated 4 month period. It is the intent of this study to enroll subjects who are considered healthy volunteers. Subjects with pre-existing clinically significant conditions are not considered normal, healthy volunteers. The target population will reflect the community at large in the Saint Louis area. Various methods of recruitment may be used such as letters, postcards, e-mails, brochures, posters, media boards, websites, social media, telephone contact, advertisements, and press releases. All forms and mechanisms of recruitment as well as the recruitment materials will be approved by the site's IRB prior to use. Retention of participants is accomplished through study reminder telephone calls, texts, emails, and mailings as per the preferences of each individual volunteer.

5.1 Subject Inclusion Criteria

Subjects must meet ALL of the following criteria to be eligible for inclusion in the study:

1. Males and females 18 to 55 years of age.

2. In good general health as confirmed by a medical history and physical exam, vital signs*, and screening laboratories conducted no more than 30 days prior to study injection administration.

   **Temperature <38 ° C, respiratory rate < 17 breaths pm, heart rate ⩽100 bpm and >54 bpm, systolic blood pressure ⩽140 mmHg and >89 mmHg, diastolic blood pressure ⩽90 mmHg and ⩾60 mmHg.*
   NOTE: Athletically trained subjects with a pulse ⩾40 may be enrolled at the discretion of the principal investigator or designated licensed clinical investigator.

3. Screening laboratory values within normal limits: sodium, potassium, ALT, AST, total bilirubin, alkaline phosphatase, creatinine, random glucose, total WBC count, hemoglobin, and platelet count.

4. Negative HIV 1/2 antibody, hepatitis B surface antigen (HBsAg), and hepatitis C virus (HCV) antibody.

5. Urine dipstick for protein and glucose (negative to trace protein are acceptable).

6. Women of childbearing potential* in sexual relationships with men must agree to practice acceptable contraception** for the 30-day period before Day 0 through 90 days after the last study injection.

   **Not sterilized via tubal ligation, bilateral oophorectomy, hysterectomy or successful Essure® placement (permanent, non-surgical, non-hormonal sterilization) with documented radiological confirmation test at least 90 days after the procedure, and still menstruating or < 1 year of the last menses if menopausal). Post-menopausal defined as at least 12 months spontaneous amenorrhea and confirmed with FSH > 40 mIU/ml.*

This document is proprietary and is to be used only as authorized. No part is to be reproduced, disclosed to others, or quoted without prior written authorization from IDRI.

***Includes, but is not limited to, sexual abstinence, monogamous relationship with vasectomized partner who has been vasectomized for 6 months or more prior to the subject receiving study product, barrier methods such as condoms or diaphragms with spermicide or foam, effective intrauterine devices, NuvaRing ®, and licensed hormonal methods such as implants, injectables or oral contraceptives ("the pill").*

7. Able to understand and comply with planned study procedures and willing to be available for all study-required procedures, visits and calls for the duration of the study.

8. Provide written informed consent before initiation of any study procedures.

9. Willing to abstain from donating whole blood or blood derivatives until 90 days after the final study injection.

5.2 Subject Exclusion Criteria

Subjects who meet any of the following criteria at screening/baseline will be excluded from study participation:

1. Previous exposure to ID93 vaccines or experimental products containing GLA-SE.

2. History of treatment for active or latent tuberculosis infection.

3. History or evidence of active or documented latent tuberculosis, or positive QuantiFERON®-TB Gold test.

4. Shared a residence within the last year prior to randomization with an individual on anti-tuberculosis treatment or with culture or smear positive tuberculosis.

5. Received a tuberculin skin test within 3 months (90 days) prior to randomization.

6. History of autoimmune disease or immunosuppression.

7. Used immunosuppressive medication (e.g., oral or injected steroids) within 3 months prior to randomization (inhaled and topical corticosteroids are permitted).

8. Received any investigational drug therapy or investigational vaccine within past 6 months prior to randomization, or planned participation in any other investigational study during the study period.

9. Received investigational TB vaccine at any time prior to randomization.

10. Received any vaccine within 30 days prior to the first study vaccination and no planned immunizations between Day 0-84 or Day 210-224 due to the washout period prior to immunology blood draws.

11. History or laboratory evidence of immunodeficiency state including but not limited to laboratory indication of HIV-1 infection at screening.

This document is proprietary and is to be used only as authorized. No part is to be reproduced, disclosed to others, or quoted without prior written authorization from IDRI.

12. History of allergic disease or reactions, likely to be exacerbated by any component of the study vaccine.

13. History of allergic reaction to kanamycin-related antibiotics.

14. Subjects with a history of previous anaphylaxis or severe allergic reaction to vaccines or unknown allergens.

15. Previous medical history that may compromise the safety of the subject in the study, including but not limited to: severe impairment of pulmonary function from tuberculosis infection or other pulmonary disease; chronic illness with signs of cardiac or renal failure; suspected progressive neurological disease; or uncontrolled epilepsy or infantile spasms.

16. Known or suspected alcohol or drug abuse within the past 5 years.

17. Smokes 1 pack or more of cigarettes per day.

18. History of keloid formation or excessive scarring.

19. History or evidence on physical examination of any systemic disease or any acute or chronic illness that, in the opinion of the investigator, may interfere with the evaluation of the safety or immunogenicity of the vaccine, including axillary lymphadenopathy.

20. Received a blood transfusion or immunoglobulin within the past 3 months prior to randomization.

21. Donated blood products (platelets, whole blood, plasma, etc.) within past 1 month prior to randomization.

22. Presence of any febrile illness, oral temperature of >100.4 °F/38.0 °C within 24 hours of study injection administration. Such subjects may be re-evaluated for enrolment after resolution of illness.

23. Positive serum (at screening visit only) or urine pregnancy test at screening or within 24 hours prior to study injection for women of childbearing potential.

24. Breastfeeding at any time throughout the study.

25. Rash, tattoos, or any other dermatological condition on the upper anterolateral arm that could adversely affect the vaccine injection site or interfere with its evaluation.

26. BMI <18 or >35 kg/m2.

27. Any medical or neuropsychiatric condition which, in the Investigator’s opinion, would render the subject incompetent to provide informed consent or unable to provide valid safety observations and reporting.

This document is proprietary and is to be used only as authorized. No part is to be reproduced, disclosed to others, or quoted without prior written authorization from IDRI.

28. Cancer or treatment for cancer within 3 years of study injection administration. Persons with a history of cancer who are disease-free without treatment for 3 years or more are eligible. Persons with treated and uncomplicated basal cell carcinoma of the skin are eligible.

29. Subjects unlikely to cooperate with the requirements of the study protocol.

Vital signs are performed after subjects have been in a seated position for five minutes, with no hot or cold beverages or smoking in the past five minutes. Vital signs may be performed up to three times to allow for transient conditions to resolve. Screening laboratory values that are abnormal, but are considered abnormal due to an acute illness or process may be repeated once. Creatinine, AST, ALT, bilirubin values that fall below the FDA standard toxicity tables (Appendix 3 - Guidance for Industry: Toxicity Grading Scale for Healthy Adult and Adolescent Volunteers Enrolled in Preventative Vaccine Clinical Trials; FDA/CBER; 2007) are not considered abnormal for the purposes of this study. Additionally, abnormalities in the RBC parameters other than hemoglobin and abnormalities in the WBC differential not specified in the inclusion criteria above (e.g. low lymphocytes, basophils, or eosinophils) will not be considered exclusionary for this study and will be followed with the standard safety laboratory follow ups outlined in the study. Laboratory values that are performed as a standard panel by the clinical laboratory, but are not requested for the study, will be reviewed by a licensed study clinician and the clinician will determine whether the laboratory value abnormality is clinically significant and should be considered exclusionary. If determined to be clinically insignificant, the study team is not required to follow the laboratory until resolution or the value is determined to be clinically stable.

5.3 Eligibility Criteria for 2nd Injection

Subjects must meet all inclusion and exclusion criteria as outlined in Sections 5.1 and 5.2 with the exception of clinical safety lab values. Subjects with safety laboratory values that meet Grade 2 or greater severity (according to the toxicity table, Appendix 3), that do not return to the protocol-defined normal range prior to the second study injection will not be eligible to receive the second study injection. Repeat HIV, hepatitis B and hepatitis C serologies are not required prior to the second study injection.

5.4 Treatment Assignment Procedures

5.4.1 Randomization Procedures

Forty-eight (48) subjects will be randomly assigned to one of two groups, as shown in Table 1. Block randomization of appropriate size will accommodate balanced enrollment in a ratio of 1:1 into each of the two groups for the planned 48 subjects.

The list of randomized treatment assignments will be prepared by statisticians at DF/Net Research and included in the enrollment module of Interactive Web Randomization System

This document is proprietary and is to be used only as authorized. No part is to be reproduced, disclosed to others, or quoted without prior written authorization from IDRI.

(IWRS). A designated individual at each site will be provided with a treatment key, which links the treatment code to the actual treatment assignment, which will be kept in a secure place.

Instructions for use of IWRS will be included in the Study Specific Procedures (SSP) Manual. Manual back-up randomization procedures will be provided in the event that the site temporarily loses access to the Internet or the online enrollment system is unavailable.

Table 1: Treatment Assignments

Group	N	Study Injections	Timing of Study Injections
1	24	ID93 (2 µg, lyophilized) + GLA-SE (5 µg, liquid) [two-vial presentation]	Days 0, 56
2	24	ID93 + GLA-SE (2 µg ID93, 5 µg GLA) [lyophilized, single-vial]	Days 0, 56

5.4.2 Blinding Procedures

This is a double-blind study. Subjects, investigators, study personnel performing any study-related assessments following study injection, and laboratory personnel performing immunology assays will be blinded to treatment assignment.

The randomization scheme will be generated by DF/Net Research and provided to unblinded study personnel (i.e., pharmacists performing study product preparations and unblinded study product administrators).

The unblinded study product administrator is a study personnel licensed to administer medications/vaccines, but will not be involved in study-related assessments or have subject contact for data collection following study injection.

The Safety Monitoring Committee (SMC) may receive data in aggregate and by group assignment, or may be unblinded to individual study treatment assignments, as needed to assess safety issues.

Refer to the Study Specific Procedures (SSP) Manual for unblinding procedures.

5.4.3 Reasons for Withdrawal

Any enrolled/vaccinated subject may withdraw or be withdrawn from the study for the following reasons:

- The subject withdraws consent
- The subject fails to comply with the scheduled study visits
- The study is terminated

This document is proprietary and is to be used only as authorized. No part is to be reproduced, disclosed to others, or quoted without prior written authorization from IDRI.

- For any reason that, in the opinion of the investigator, precludes the subject's participation in the study
- Medical disease or condition, or any new clinical finding for which continued participation, in the opinion of the site PI or appropriate sub-investigator, would compromise the safety of the subject, or would interfere with the subject's successful completion of this trial, or would interfere with the evaluation of responses (for example, has significant laboratory abnormalities)
- Subject lost to follow-up
- Subject no longer meets eligibility criteria

Subjects may withdraw from participation in the study at any time. If a subject withdraws or is withdrawn prior to completion of the study, the reason for this decision must be recorded in the electronic case report forms (eCRFs). Refer to Section 7.11 for procedures to be followed if a subject withdraws from the study.

5.4.4 Handling of Withdrawals

In the case of subjects who fail to appear for a follow-up assessment, extensive efforts (i.e., documented phone calls and certified mail) should be made to locate or recall them, or at least to determine their health status. These efforts should be documented in the subjects' records and their health status documented in the appropriate eCRF.

Subjects who withdraw from the study after randomization but prior to the first study injection may be replaced. Subjects who withdraw from the study after the first study injection will not be replaced.

5.4.5 Handling of Subjects Withdrawn from Further Study Injections

A volunteer may withdraw voluntarily from or be withdrawn from receiving further study injections, but will be encouraged to permit continued follow-up of AEs and to donate scheduled blood samples. If the volunteer agrees, other study procedures (e.g., blood sampling for safety and immunogenicity responses) may be continued. Refer to Section 7.13 for the visit schedule to be followed if second study injection is withheld.

Withdrawal from receiving further study injections will occur if:

- Any rule for discontinuing study injection in an individual subject is met (Section 9.6.1)
- The subject no longer meets inclusion/exclusion criteria
- The subject develops a serious adverse event that warrants withdrawal
- The subject becomes pregnant

This document is proprietary and is to be used only as authorized. No part is to be reproduced, disclosed to others, or quoted without prior written authorization from IDRI.

- Other safety-related reasons at the discretion of the PI, IDRI Medical Monitor, DMID Medical Monitor, SMC, or the subject

5.4.6 Termination of Study

The sponsor IDRI, the IRB, or the FDA have the right to terminate this study at any time. Reasons for terminating the study may include, but are not limited to, the following:

- Incidence or severity of AEs indicating a potential health hazard
- Data recording is inaccurate or incomplete
- The Investigator has not been adhering to the protocol or applicable regulatory guidelines in conducting the study

This document is proprietary and is to be used only as authorized. No part is to be reproduced, disclosed to others, or quoted without prior written authorization from IDRI.

6 STUDY INTERVENTION/INVESTIGATIONAL PRODUCT

6.1 Study Product Description

ID93 for Injection

Studies by investigators in the TB research program at IDRI have resulted in the identification of a recombinant subunit vaccine antigen (ID93) formulated as a fusion protein from four Mtb proteins associated with virulence and latency (Rv2608, Rv3619, Rv3620, and Rv1813). The final 891 amino acid fusion protein has a predicted mass of 93KDa.

GLA-SE Adjuvant (also known as AP 10-201)

Glucopyranosyl Lipid A (GLA) is a synthetic Monophosphoryl Lipid A-like molecule (MPL®; GSK Biologicals) which is a Toll-like receptor 4 (TLR4) agonist. GLA is formulated in a stable oil-in-water emulsion (SE) to produce GLA-SE. Due to the TLR4 activity of the GLA molecule, the combination of GLA-SE with a recombinant protein antigen (ID93) results in a Th1-type T cell response, which is important for protection from infection with Mtb.

ID93 + GLA-SE for Injection

The single-vial presentation of ID93 + GLA-SE contains the antigen and adjuvant components formulated together and lyophilized in the same vial. After reconstitution with water-for-injection, it is ready for administration to the study participant.

6.1.1 Acquisition

All investigational products will be supplied by IDRI and shipped to the clinical site at Saint Louis University. Water for Injection, USP diluent (WFI) will be supplied by the clinical site.

6.1.2 Formulation, Storage, Packaging, and Labeling

ID93 for Injection

ID93 for Injection is formulated as a lyophilized solid and appears as a white to off-white cake. Each 2 mL glass vial contains 100 µg of ID93 antigen and must be stored at 2-8°C. Reconstitution of ID93 with WFI results in a clear solution. After reconstitution, vials may be held at ambient temperature while preparing doses for injection. Vials are for single-use only. For the two-vial presentation, ID93 is vialed separately and labeled “ID93 for Injection”.

This document is proprietary and is to be used only as authorized. No part is to be reproduced, disclosed to others, or quoted without prior written authorization from IDRI.

GLA-SE Adjuvant (also known as AP 10-201)

GLA-SE Adjuvant (labeled as "AP 10-201") is formulated as 20 µg/mL GLA in 4% stable oil-in-water emulsion and supplied as single use glass vials. GLA-SE appears as a milky-white liquid. Each 2 mL vial contains a fill volume of 0.4 mL and must be stored at 2-8°C. Do not freeze. For the two-vial presentation, GLA-SE is vialed separately and labeled "AP 10-201".

ID93 + GLA-SE for Injection

ID93 + GLA-SE is formulated as a lyophilized solid, appears as a white to off-white cake, and must be stored at 2-8°C. Do not freeze. Each 3 mL glass vial, when reconstituted with 0.8 mL WFI, contains 4 µg/mL of ID93 antigen and 10 µg/mL GLA in 2% stable oil-in-water emulsion. Reconstitution of ID93 + GLA-SE for Injection with WFI results in a milky-white solution. For the single-vial presentation, that includes both ID93 and GLA-SE, each vial is labeled "ID93 + GLA-SE for Injection".

Each of the study products containing ID93 antigen and/or GLA-SE adjuvant will be labeled according to manufacturer specifications and include the statement "Caution: New Drug – Limited by Federal Law to Investigational Use."

Sterile Water for Injection, USP

The sterile WFI sourced by the clinical site should be nonpyrogenic and contain no bacteriostatic, antimicrobial agent, or added buffer. This product should be used to reconstitute the antigen ID93 for Injection and for reconstituting the single-vial ID93 + GLA-SE, and will be supplied as single-use containers. The sterile WFI vials are stored at 20 to 25ºC (68 to 77ºF) [See USP Controlled Room Temperature; excursions between 15 and 30°C (59 and 86°F) are permitted]

6.2 Dosage, Preparation, and Administration of Study Intervention/Investigational Product

Study vaccine preparation, including vaccine dilutions and admixing for the various dosing groups, will be performed by the unblinded site pharmacist using aseptic technique under a biological safety cabinet (BSC) or laminar flow hood on the same day of study vaccine administration.

Long-term stability of mixtures of ID93 and GLA-SE is unknown at this time. Therefore, all study injection preparations must be administered within 2 hours of preparation. The 2 hour window begins when the final formulated preparation is mixed and drawn into a syringe for administration to a study participant. The time of preparation and time of administration should be noted in the source documentation.

Vials of antigen and adjuvant should be removed from the 2-8°C storage refrigerator at least 5 minutes prior to study injection preparation to allow equilibration with room temperature. The vials and prepared syringes may be maintained at room temperature (below 37°C) during preparation

This document is proprietary and is to be used only as authorized. No part is to be reproduced, disclosed to others, or quoted without prior written authorization from IDRI.

time and prior to administration. The designee assigned to administer the study injections will be supplied with a loaded syringe labeled with the participant identification number (PID#) and hospital required identification data.

All vials are single-use only.

All used and labeled vials for each subject per dosing day should be placed in a sealable bag. The bag should be labeled with PID#(s) and the date of injection. The used vials must be kept in a secured location. Used vials may be kept at room temperature or refrigerated until drug accountability is completed by the unblinded investigational product monitor.

Refer to the SSP Manual for detailed information on the preparation, labeling, and storage of study product.

6.3 Modification of Study Intervention/Investigational Product for a Participant

No modification of the study intervention or investigational product will occur for individual participants.

6.4 Accountability Procedures for the Study Intervention/Investigational Product(s)

After receipt of the investigational products, the site PI is responsible for study product distribution and disposition, and has ultimate responsibility for study product accountability. The PI may delegate to the site Research Pharmacist the responsibility for study product accountability. The site Research Pharmacist will be responsible for maintaining complete records and documentation of product receipt, accountability, dispensation, temperature and storage conditions, and final disposition of study product. All study product, whether administered or not, must be documented on the appropriate study product accountability record or dispensing log.

Used and unused vials of investigational product will be retained until monitored and released for disposition as applicable. Upon completion or termination of the study and after the final monitoring visit, final disposition of the unused study product will be determined by the sponsor and communicated to the participating site(s). For details regarding final disposition of study products, see the SSP Manual.

6.5 Concomitant Medications/Treatments

Administration of any medications, therapies, or vaccines will be documented in the subject's source documentation and reported on the subject's electronic case report form (eCRF). Concomitant medications will include all medications, vitamins, and supplements taken within the 30 days before enrollment and through 28 days after the last study injection (approximately Day 84) or early termination, whichever occurs first.

This document is proprietary and is to be used only as authorized. No part is to be reproduced, disclosed to others, or quoted without prior written authorization from IDRI.

Medications that might interfere with the evaluation of the investigational product should not be used unless necessary. Medications in this category include, but are not limited to, glucocorticoids (i.e., oral, parenteral, and high-dose inhaled steroids), and immunosuppressive or cytotoxic drugs. Analgesics and antipyretics should be avoided for the 24 hours following study injection, unless necessary.

This document is proprietary and is to be used only as authorized. No part is to be reproduced, disclosed to others, or quoted without prior written authorization from IDRI.

7 STUDY SCHEDULE

7.1 Screening Procedures (Day -30 to Day -1)

More than one clinic visit may be required in order to complete all screening evaluations for an individual subject. The following procedures will be performed:

- Obtain informed consent. This should be performed prior to conducting any of the assessments listed below.
- Assign a Screening Identification number (SID #).
- Review inclusion/exclusion criteria as specified for the initial study injection.
- Obtain medical history, and concomitant medications (prescription and over-the counter drugs taken 30 days prior to enrollment will be reviewed and documented).
- Perform a physical exam that assesses general appearance and the following areas/systems: skin, lymph nodes, HEENT, neck, pulmonary/respiratory, cardiovascular, abdomen, musculoskeletal, and neurological.
- Obtain height and weight. Calculate BMI.
- Obtain vital signs including blood pressure (BP), pulse (P), respiratory rate (RR), and oral temperature.
- Assess visual acuity (Snellen test or other objective measure of visual acuity)
- Obtain blood and urine sample for screening laboratories and QuantiFERON®-TB Gold test.
- Perform serum pregnancy test for female subjects of childbearing potential or serum FSH for post-menopausal females.
- Review of contraceptive methods (female subjects of childbearing potential only).
- Counseling on avoidance of pregnancy (for all female subjects of childbearing potential).
- All screening laboratory results must be available for review by the investigator prior to visit Day 0.

7.2 Day 0 Enrollment/Baseline Evaluations and First Study Injection

Prior to Study Injection:

- Review Inclusion/Exclusion criteria as specified for the initial study injection.
- Review concomitant medications.
- Obtain vital signs (BP, P, RR, oral temperature).
- Perform a targeted physical exam if indicated based on symptoms.
- Perform urine pregnancy test for females of childbearing potential within 24 hours prior to study injection. Test results must be confirmed as negative prior to study injection.
- Review of acceptable contraceptive methods (female subjects only).
- Counseling on avoidance of pregnancy (for all female subjects of childbearing potential).
- Collect nasal swab
- Collect tears
- Collect blood prior to study injection for baseline immunologic evaluations for:
    - Serum for IgG ELISA
    - Serum to bank (baseline for auto-immune testing, if indicated)
    - Whole blood for MGIA
    - PBMC isolation

This document is proprietary and is to be used only as authorized. No part is to be reproduced, disclosed to others, or quoted without prior written authorization from IDRI.

- Perform randomization procedures.

Study Injection:
- Administer study injection in the non-dominant deltoid unless there is a reason to inject in the alternate deltoid. Both injections are preferably administered in the same arm.
- Dispense memory aid, measuring tool, and thermometer.

Immediately Following Study Injection:

All subjects will be observed for a minimum of 60 minutes following study injection. After 60 minutes:
- Obtain vital signs (BP, P, RR, oral temperature).
- Examine the injection site and assess any AE/SAEs (including specific solicited systemic reactions: headache, arthralgia, chills, loss of appetite, fever, fatigue, and myalgia), prior to discharge from the clinic.
- Request subject to bring their completed memory aid to the next clinic visit.

All subjects will be asked to record, on a subject memory aid, daily maximum temperatures, symptoms for 7 days, measure erythema or induration if any, beginning with the day of study injection and report them to the clinic staff. Subjects will be asked to bring their completed memory aid to the Day 7 post-injection clinic visit.

7.3 Day 7 (±1) Evaluations
- Obtain vital signs (BP, P, RR, oral temperature).
- Evaluate site of injection.
- Perform a targeted physical exam if indicated, based on symptoms.
- Review memory aid.
- Review adverse events, including SAEs and PIMMCs.
- Review concomitant medications
- Collect blood for:
  - Hematology: WBC with differential, HGB, platelets
  - Serum chemistry: includes sodium, potassium, ALT, AST, total bilirubin, alkaline phosphatase, and creatinine.
  - PBMC isolation

7.4 Day 14 (±3) Evaluations
- Collect blood for:
  - Serum for IgG ELISA
  - PBMC isolation

7.5 Day 56 (±3) Evaluations and Second Study Injection

Prior to Study Injection:
- Review Inclusion/Exclusion criteria as specified for the second study injection.
- Review adverse events, including SAEs and PIMMCs.

This document is proprietary and is to be used only as authorized. No part is to be reproduced, disclosed to others, or quoted without prior written authorization from IDRI.

- Review concomitant medications.
- Obtain vital signs (BP, P, RR, oral temperature).
- Perform a targeted physical exam if indicated, based on symptoms.
- Perform urine pregnancy test for females of childbearing potential within 24 hours prior to study injection. Test results must be confirmed as negative prior to study injection.
- Review of acceptable contraceptive methods (female subjects only).
- Counseling on avoidance of pregnancy (for all female subjects of childbearing potential).
- Collect blood prior to study injection for baseline immunologic evaluations for:
    - Serum for IgG ELISA
    - PBMC isolation

Study Injection:

- Administer study injection in the non-dominant deltoid unless there is a reason to inject in the alternate deltoid. Both injections are preferably administered in the same arm.
- Dispense memory aid, measuring tool, and thermometer.

Immediately Following Study Injection:

All subjects will be observed for a minimum of 60 minutes following study injection. After 60 minutes:

- Obtain vital signs (BP, P, RR, oral temperature).
- Examine the injection site and assess any AE/SAEs (including specific solicited systemic reactions: headache, arthralgia, chills, loss of appetite, fever, fatigue, and myalgia), prior to discharge from the clinic.
- Request subject to bring their completed memory aid to the next clinic visit.

All subjects will be asked to record, on a subject memory aid, daily maximum temperatures, symptoms for 7 days, and measure erythema or induration if any beginning with the day of study injection and report them to the clinic staff. Subjects will be asked to bring their completed memory aid to the Day 7 post-injection clinic visit.

7.6 Day 63 (±1) Evaluations

- Obtain vital signs (BP, P, RR, oral temperature).
- Evaluate site of injection.
- Perform a targeted physical exam if indicated, based on symptoms.
- Review memory aid.
- Review adverse events, including SAEs and PIMMCs.
- Review concomitant medications.
- Collect blood for:
    - Hematology: WBC with differential, HGB, platelets.
    - Serum chemistry: includes sodium, potassium, ALT, AST, total bilirubin, alkaline phosphatase, and creatinine.
    - PBMC isolation

7.7 Day 70 (±3) Evaluations

- Collect nasal swab
- Collect tears

This document is proprietary and is to be used only as authorized. No part is to be reproduced, disclosed to others, or quoted without prior written authorization from IDRI.

- Collect blood for:
  - Serum for IgG ELISA
  - Whole blood for MGIA
  - PBMC isolation

7.8 Day 84 (±5) Evaluation

- Obtain vital signs (BP, P, RR, oral temperature).
- Perform a targeted physical exam if indicated, based on symptoms.
- Review adverse events, including SAEs and PIMMCs.
- Review concomitant medications.
- Collect blood for:
  - Serum for IgG ELISA
  - PBMC isolation

7.9 Day 224 (±10) Evaluations

- Perform a targeted physical exam if indicated, based on symptoms.
- Review SAEs and PIMMCs.
- Record concomitant medications used for treatment of SAEs or PIMMCs, if any.
- Collect nasal swab
- Collect tears
- Collect blood for:
  - Serum for IgG ELISA
  - Whole blood for MGIA
  - PBMC isolation

7.10 Day 421 (±14) Evaluations

The follow-up at one year after the last study injection will be performed by telephone.

- Review SAEs and PIMMCs.
- Record concomitant medications used for treatment of SAEs or PIMMCs, if any.

7.11 Early Termination Visit

If a subject withdraws from the study early, the following procedures should be performed at the early termination visit, if possible.

- Review current health status (interim medical history) and note any changes since the last visit.
- Obtain vital signs (BP, P, RR, oral temperature). All concomitant medications will be recorded if before Day 84 visit.
- Collect blood for (refer to Schedule of Blood Draws and Volumes):
  - Serum for IgG ELISA
  - PBMC isolation

  If prior to Day 63, for:
  - Hematology: WBC with differential, HGB, platelets

This document is proprietary and is to be used only as authorized. No part is to be reproduced, disclosed to others, or quoted without prior written authorization from IDRI.

  - Serum chemistry: includes sodium, potassium, ALT, AST, total bilirubin, alkaline phosphatase, and creatinine
- A targeted physical examination may be performed, if indicated.
- Information regarding AEs, SAEs, and new onset chronic medical conditions will be solicited (AEs will be solicited only if prior to Day 84). Any ongoing AEs or PIMMCs will be followed to resolution or until a stable chronic condition has been established.

Subjects may withdraw voluntarily from participation in the study at any time. Subjects may also withdraw voluntarily from receiving the study intervention for any reason. An investigator may also withdraw a subject from receiving further study intervention. The subject may be withdrawn from the study if the sponsor terminates the study.

Follow-up assessments will be completed according to the protocol schedule, if possible.

7.12 Unscheduled Visit

Unscheduled visits may occur at any time during the study. The procedures below are to be performed at any unscheduled visit. See the SSP Manual for instructions for documentation and data reporting.

- Review concomitant medications.
- Obtain vital signs, if clinically indicated.
- Perform a targeted physical exam if indicated, based on symptoms.
- Blood and/or urine samples will be collected for safety laboratory tests, if indicated
- Evaluate injection site, if within 7 days of injection or if indicated.
- Review memory aid and adverse events if within 7 days of injection or if ongoing local or systemic reactogenicity on day 7 post study injection.
- Review adverse events, if within 84 days of first injection or if indicated.
- Review SAEs and PIMMCs

7.13 Visit Schedule to be followed if 2nd study injection is withheld

Subjects who receive only the first study injection will continue to be followed for 28 days after the injection, and then a telephone visit will be performed on Day 421. The appropriate scheduled blood samples will be obtained for these visits as outlined in the study visit schedule.

This document is proprietary and is to be used only as authorized. No part is to be reproduced, disclosed to others, or quoted without prior written authorization from IDRI.

8 STUDY PROCEDURES AND EVALUATIONS

8.1 Clinical Evaluations

The schedule of study visits and procedures is specified in Appendix 1.

Medical History: Will be obtained by interview of the subjects. Subjects will be queried regarding a history of significant medical disorders of the head, eyes, ears, nose, throat, mouth, cardiovascular system, lungs, gastrointestinal tract, liver, pancreas, kidney, nervous system, blood, lymph glands, endocrine system, musculoskeletal system, skin, and genital/reproductive tract. A history of any allergies, cancer, immunodeficiency, psychiatric illness, substance abuse, and autoimmune disease will be solicited. The collection of medical history information will include a review of TB vaccine history and plans for vaccinations.

Concomitant Medications: All current medications and medications taken in the 30 days before enrollment (prescription and over-the-counter drugs) will be included, as well as vitamins and supplements, through 28 days after the last study injection, unless if used for treatment of an SAE or PIMMC. In the latter case, it will be included through the end of the study. Assessment of eligibility also will include a review of prohibited medications (per the exclusion criteria).

Physical Examination: This examination will be conducted at Screening and will assess general appearance including height and weight (at Screening only), vital signs (blood pressure, oral temperature, pulse), and the following areas/symptoms: skin, lymph nodes, head, eyes, ears, nose, throat, neck, respiratory, cardiovascular, pulmonary, abdomen, extremities, musculoskeletal, and neurological.

Targeted Physical Examination: This may be conducted at any study visit based on indicated symptoms. No examination is necessary if no symptoms are present.

Local and systemic reactogenicity assessments: This will include a brief review for assessment of AEs, which includes an assessment of pain, erythema, and induration at the injection site; and systemic reactions including headache, arthralgia, chills, loss of appetite, fever, fatigue, and myalgia.

Memory Aids: All subjects will complete a subject memory aid for 7 days following each study injection. Subject memory aids will be reviewed with the subject for AEs at the clinic visit following each study injection. If a subject noted ongoing local or systemic reactogenicity on day 7 post study injection, the memory aid will continue to be used until resolved. The memory aids will be retained in the subject source document binder after review. A measuring tool and thermometer are included with the memory aid.

This document is proprietary and is to be used only as authorized. No part is to be reproduced, disclosed to others, or quoted without prior written authorization from IDRI.

8.2 Laboratory Evaluations

Schedules and volumes of blood to be collected for clinical laboratory tests and immunogenicity assays are specified in Appendix 2.

8.2.1 Clinical Laboratory Evaluations

Clinical safety laboratory evaluations will be obtained at Screening and on Days 0, 7, 56, and 63. Specific tests to be performed are described below:

Screening Visit:

- Hematology: WBC with differential, HGB, platelets
- Chemistry: sodium, potassium, ALT, AST, total bilirubin, alkaline phosphatase, creatinine, random glucose.
- Serology tests: HIV 1/2 antibody, hepatitis B surface antigen (HBsAg), and hepatitis C virus (HCV) antibody.
- Urinalysis: will be performed by dipstick testing (includes protein and glucose).
- Pregnancy test: urine pregnancy test in females of childbearing potential or serum FSH for post-menopausal females.

Days 7 and 63:

- Hematology: WBC with differential, HGB, platelets.
- Chemistry: sodium, potassium, ALT, AST, total bilirubin, alkaline phosphatase, and creatinine.

Days 0 and 56:

- Pregnancy test: urine pregnancy tests on Days 0 and 56 in females of childbearing potential.

One or more of the laboratory parameters may be repeated at any time during the study as determined by the PI or licensed clinical study physician, if indicated by an AE. Screening laboratory values that are abnormal, but are considered abnormal due to an acute illness or process may be repeated once. A clinically significant abnormal value should be repeated within 10 days if possible and followed up as clinically relevant.

8.2.2 Special Assays or Procedures

Screening Visit:

- Prior exposure to TB antigens (prior infection with Mtb or vaccination with BCG) will be assessed using QuantiFERON®-TB Gold tests at the screening visit. Positive results will result in exclusion from the study and referral to medical treatment.

Days 0, 14, 56, 70, 84, 224:

- IgG antibody response in serum (secondary outcome measure)

This document is proprietary and is to be used only as authorized. No part is to be reproduced, disclosed to others, or quoted without prior written authorization from IDRI.

    - ID93-specific antibody responses will be measured by IgG binding to the ID93 protein using ELISA. Studies will be performed at IDRI.
- ELISpot and ICS assays in PBMC (secondary outcome measures)
    - T cell immune responses will be assessed by enumerating antigen-specific, cytokine-secreting T cells using IFN-γ enzyme-linked immunosorbent spot assays (ELISpot) and flow cytometry-based intracellular cytokine staining (ICS) from PBMC samples. Both assays will be performed at IDRI.

Days 0, 70, 224:

- IgA antibody response in mucosal secretions (exploratory outcome measure)
    - Antibody responses will be measured in nasal swab and tear specimens by IgA binding to the ID93 protein using ELISA. Assay will be performed by Dan Hoft's laboratory at Saint Louis University.

Days 0, 70, 224:

- Mtb growth inhibition in whole blood (exploratory outcome measure)
    - Functionality of the immune response will be assessed by measuring the capacity of whole blood to inhibit the growth of mycobacteria. Assay will be performed by Dan Hoft's laboratory at Saint Louis University.

Days 0, 7, 14, 63, 70, 224:

- Exploratory cellular immune responses (exploratory outcome measures)
    - PBMC samples will be used to quantitate the number of antibody-secreting cells in PBMC by short-culture B cell ELISpot, the number of antigen-specific memory B cells in PBMC by long-culture B cell ELISpot, and the frequencies of T follicular helper cells, memory B cells, and T cell homing markers in PBMC by immunophenotyping flow cytometry. Studies will be performed at IDRI.

8.2.3 Specimen Shipment

All participant samples (except for whole blood specimens) will be processed and stored through Study Day 224. Whole blood specimens are used fresh by the site and therefore not stored. Mucosal specimens will remain at the site for analysis (tears, nasal swabs). PBMC and serum specimens will be shipped to IDRI for analysis after the last Day 224 specimens are stored. PBMC and serum specimens from a single participant will be divided into separate boxes for storage and shipment. Shipment of PBMC and serum samples from the same individual and time point will take place on separate dates in case of specimen loss due to temperature excursions/deviation in cold chain.

This document is proprietary and is to be used only as authorized. No part is to be reproduced, disclosed to others, or quoted without prior written authorization from IDRI.

9 ASSESSMENT OF SAFETY

9.1 Specification of Safety Parameters

Safety will be assessed by the frequency and severity of:

1. Serious adverse events occurring from the time of the first study injection through the entire study period.

2. Solicited adverse events – reactogenicity events occurring on the day of each study injection through 7 days after each study injection. This time period may be extended if a subject notes ongoing local or systemic reactogenicity on day 7 post study injection:

   a) Injection site reactions including pain, induration (hardening/swelling), and erythema (redness).

   b) Systemic reactions including headache, arthralgia (generalized joint pain), chills, loss of appetite, fever, fatigue, and myalgia (generalized muscle pain).

3. Unsolicited adverse events – non-serious adverse events occurring from the time of the first study injection through Day 84, including vital sign and safety laboratory abnormalities.

4. New-onset potential immune-mediated medical conditions (PIMMCs) occurring from the time of the first study injection through the entire study period.

9.2 Methods and Timing for Assessing, Recording, and Analyzing Safety Parameters

9.2.1 Adverse Events

Adverse Event: Adverse event means any untoward medical occurrence associated with the use of a drug in humans, whether or not considered drug or study injection related. An adverse event (also referred to as an adverse experience) can be any unfavorable and unintended sign (e.g., an abnormal laboratory finding), symptom, or disease temporally associated with the use of a drug, and does not imply any judgment about causality. An adverse event can arise with any use of the drug (e.g., off-label use, use in combination with another drug) and with any route of administration, formulation, or dose, including an overdose.

All conditions that exist prior to administration of the study injection (pre-existing conditions) will be recorded in the participant's medical history to establish baseline. Day-to-day fluctuations in pre-existing conditions that do not represent a clinically significant change in the participant's status will not necessarily be reported as adverse events.

This document is proprietary and is to be used only as authorized. No part is to be reproduced, disclosed to others, or quoted without prior written authorization from IDRI.

Any adverse change from the participant’s baseline condition (determined from screening evaluations conducted to confirm study eligibility) that occurs following the administration of the study injection will be considered an adverse event. This includes the occurrence of a new adverse event or the worsening of a baseline condition, whether or not considered related to the study injection. Intermittent conditions such as headaches may be present on Study Day 0, but may represent an adverse event if the frequency, intensity, or duration of the event is worse than usual following receipt of study injection. Adverse events include but are not limited to: adverse changes from baseline that represent increases in toxicity grade, adverse changes in the general condition of the participant, signs and symptoms noted by the participant, concomitant disease with onset or increased severity after study injection administration, and changes in laboratory safety parameters occurring after study injection administration.

A ***suspected adverse reaction*** is any adverse event for which there is a *reasonable possibility* that the study injection caused the event. Suspected adverse reactions are a subset of all adverse events. For example, headache or fever following study injection.

An ***adverse reaction*** is any adverse event *definitely* caused by the study injection. Adverse reactions are a subset of all suspected adverse reactions. For example, local site of injection erythema or pain at the site of injection.

Unexpected Adverse Event: An adverse event is considered “unexpected” if it is not listed in the Investigator’s Brochure or is not listed at the specificity or severity that has been observed.

Serious Adverse Event: An adverse event is considered a *serious* adverse event (SAE) if, in the view of either the investigator or sponsor, it results in any of the following outcomes:

- death,
- a life-threatening adverse event,
- inpatient hospitalization or prolongation of existing hospitalization,
- persistent or significant disability/incapacity or substantial disruption of the ability to conduct normal life functions,
- a congenital anomaly/birth defect in the offspring of a study subject.

Important medical events that may not result in death, are not life-threatening, or do not require hospitalization may be considered an SAE when, based upon appropriate medical judgment, they may jeopardize the subject and may require medical or surgical intervention to prevent one of the outcomes listed in this definition. Examples of such medical events include allergic bronchospasm requiring intensive treatment in an emergency room or at home, blood dyscrasias or convulsions that do not result in inpatient hospitalization, or the development of drug dependency or drug abuse.

NOTE: A distinction should be drawn between serious and severe events. A severe event is a major experience of its type. A severe event does not necessarily need to be serious. For example, nausea, which persists for several hours, may be considered severe nausea but not a serious adverse event. On the other hand, a stroke that results in only a limited degree of disability may be considered a mild stroke but would be a serious adverse event.

This document is proprietary and is to be used only as authorized. No part is to be reproduced, disclosed to others, or quoted without prior written authorization from IDRI.

Life-threatening Event: An adverse event is considered "life-threatening" if, in the view of either the investigator or sponsor, its occurrence places the patient or subject at immediate risk of death. It does not include an adverse event that, had it occurred in a more severe form, might have caused death.

Potential immune-mediated medical conditions (PIMMCs)*:* FDA requires monitoring for specified known or suspected autoimmune disorders when testing novel adjuvants. PIMMCs will be recorded on the AE form and reported to IDRI Pharmacovigilance regardless of their attributed relationship to study injections. PIMMCs consist of any of the diagnoses listed in Appendix 6 reported in study injection recipients at any time during the study period or after completion of the study.

All adverse events (including PIMMCs) will be reported on the Adverse Event eCRF using a recognized medical term or diagnosis that accurately reflects the event. Adverse event evaluations will be reviewed by the PI or by a designated medically qualified practitioner. Adverse event eCRF pages are to be completed by members of the study team designated in writing by the PI. The onset and resolution dates of the event and action taken in response to the event will be documented. All adverse events must be followed until resolution or stabilization is demonstrated. The resolution date will be recorded on the eCRF as the last date on which the participant experienced the adverse event. If the subject cannot recall the date of resolution, then the resolution date will be the visit date on which the study staff determines the AE to be resolved.

Adverse events that are still present at the end of the trial should be recorded as ongoing and be followed until resolved or considered stable. Information recorded on the eCRF must be substantiated in the source documents. If an adverse event evolves into a condition that becomes "serious," it will be designated as serious on the Adverse Event eCRF and an SAE Report will be completed.

9.2.2 Assessment of Severity (Grading)

The safety concepts of "severity" and "seriousness" are distinct concepts. Severity refers to a degree of clinical manifestation. "Seriousness" refers to defined outcomes from an adverse event. A severe adverse event is not always serious and a serious adverse event is not always severe.

For all adverse events, the investigator is responsible for assessing the severity of the event and the causal relationship of the event to the study injection.

The severity of all adverse events, including clinical findings and abnormal laboratory values, will be classified as one of the following grades:

- Grade 1: Mild
- Grade 2: Moderate
- Grade 3: Severe

This document is proprietary and is to be used only as authorized. No part is to be reproduced, disclosed to others, or quoted without prior written authorization from IDRI.

Toxicity tables are provided in the protocol appendices for the assessment of severity of specified adverse events. The toxicity table adverse event grades do not correlate directly with the classical severity grades of mild, moderate, and severe. For the purposes of recording events on the eCRF, Grade 1 events will be considered mild in severity, Grade 2 events will be considered moderate in severity, and Grade 3 events will be considered as severe. In the grading scale for local reactions (Appendix 4), certain local reactions such as erythema and induration are graded according to size. Laboratory values are graded according to level of deviation from the normal range.

For adverse events not listed in the toxicity tables, determination of severity requires some level of interpretation as outlined below. The degree of incapacity caused by the adverse event and the level of medical intervention required for treatment may be helpful in assessing the overall severity of the adverse event.

For example:

- “Mild” events are generally regarded as noticeable but have no impact on normal activities; they may or may not require over-the-counter treatment managed by the participant.
- “Moderate” events generally have some impact on an individual’s normal daily activities and may require general symptomatic medical intervention by a healthcare professional or by the participant.
- “Severe” adverse events may be incapacitating, leading to suspension of normal daily activities, and would generally require more immediate medical evaluation and intervention by a healthcare professional.

A change in severity of an adverse event will not be recorded as a new adverse event. Only the highest severity level that occurs during the entire period of the adverse event will be recorded on the eCRF with the onset and resolution dates encompassing the entire duration of the event.

9.2.3 Assessment of Causality (Relatedness to Study Injection)

For all adverse events, the investigator will determine a causal relationship to the study injection. A number of factors will be considered in making this assessment, including: 1) the temporal relationship of the event to the administration of the study injection, 2) whether an alternative etiology has been identified including the toxicity profile of any concomitant medication, and 3) biological plausibility.

The investigator will use the following guidelines to assess the causal relationship of an adverse event to study injection:

- **Not Related** to study injection (i.e., there is not a reasonable possibility that the administration of the study product caused the adverse event).
- **Possibly Related** to study injection (i.e., there is a reasonable possibility that the adverse event was caused by study injection. There must be a plausible mechanism for the event

This document is proprietary and is to be used only as authorized. No part is to be reproduced, disclosed to others, or quoted without prior written authorization from IDRI.

to be related to study injection. The evidence is inadequate to accept or reject, or favors rejection of, a causal relationship; an association exists between the event and the study injection but there may also be an alternative etiology, such as characteristics of the participant's clinical status or underlying condition).

- **Probably Related** to study injection (i.e., it is likely that the adverse event was caused by administration of the study injection. The evidence favors acceptance of a causal relationship; an association exists between the event and receipt of the study injection and there is a plausible mechanism for the event to be related to the study injection, and an alternative etiology is not apparent).
- **Definitely Related** to study injection (i.e., the study injection is known to be the cause of the adverse event. The evidence establishes a causal relationship; an association exists between the event and receipt of the study injection and there is a plausible mechanism for the event to be related to the study injection, and causes other than the study injection have been ruled out).

For reporting purposes, consistent with NIAID guidelines, definitely, probably, and possibly related adverse events are considered **related** to study injection. Not related adverse events are considered **unrelated**.

For adverse events requiring immediate reporting, the PI and the Sponsor Medical Monitor both determine causality. It is expected that communication and consultation may occur in the assessment of the causality of adverse events. The greatest degree of causal relationship (definite > probable > possible > not related) determined by either the investigator or Medical Monitor after their discussions will determine the ultimate classification of the adverse event. Adverse events where the causality is not assessable will be considered related to the study injection.

Every effort should be made by the investigator to determine the existence of any pre-existing conditions that must be taken into consideration when assessing causal relationship of an adverse event. Pre-existing conditions should be recorded in the eCRF as baseline history and substantiated by appropriate source documentation. Intermittent conditions such as headaches in adults may not be present on Study Day 0 but may represent an adverse event if the frequency, intensity, or duration of the event is worse than usual following study injection.

9.2.4 Evaluation of Expectedness

Expected adverse events are adverse events consistent with the applicable product information provided by the sponsor (the Investigator's Brochure for an investigational product). The sponsor, in the person of the Sponsor Medical Monitor, determines expectedness. If the assessment is that the adverse event is expected, no further action is required. If the Sponsor Medical Monitor's assessment is that the adverse event is unexpected, then the event may represent a suspected unexpected serious adverse reaction (SUSAR) or expedited SAE.

This document is proprietary and is to be used only as authorized. No part is to be reproduced, disclosed to others, or quoted without prior written authorization from IDRI.

9.2.5 Reactogenicity

Local site of injection reactions (pain, erythema, and induration) will be graded according to Appendix 4. Specific solicited systemic reactions (headache, arthralgia, chills, loss of appetite, fever, fatigue, and myalgia) will be graded according to Appendix 5.

9.2.6 Memory Aids

Memory aid booklets will be distributed to each subject to collect local and systemic adverse event information covering the 7-day period following the first and second study injections. All subjects will be instructed to record local and general signs and symptoms on individual memory aids on the evening of the day of injection and at approximately the same time daily for each of the six days following injection. Erythema and induration at the injection site will be measured in centimeters, using the measuring tool provided for that purpose. Subjects will also be asked to record any medications taken. The memory aid will be brought to the clinic on the scheduled visit 7 days after each injection for review and will be collected by the study research staff on Days 7 and 63. The memory aid is a tool to help aid the PI and/or designee to engage in a conversation with the subject about any AEs that may have occurred between visits. Memory aids not brought to the scheduled visit should be obtained before complete adverse event assessment can be performed and events discussed with the PI and/or designee. Adverse events obtained from the memory aid, as determined by the PI or designee, will not be directly recorded onto eCRFs. Any entry recorded by the subject on the memory aid that differs from the opinion of the investigator's evaluation of the event (e.g., the severity level of an event is changed after interviewing the subject) must be explained by notation in source documentation.

Subject-assessed symptoms related to the injection site in the memory aid will include injection site pain, redness (erythema), and hardening/swelling (induration).

Grading of ***local pain*** at the injection site will be according to the following definitions:

- None
- Mild (does not interfere with normal* daily activities)
- Moderate (repeated use of non-narcotic pain reliever > 24 hours or interferes with normal* daily activities)
- Severe (any use of narcotic pain reliever or prevents normal* daily activities)

Grading of ***redness*** and ***hardening/swelling*** at the injection site will be made according to the following (note: measurements are at the widest diameter):

- None (0 to < 2.5 cm in diameter)
- Mild (2.5 to 5 cm in diameter)
- Moderate (5.1 to 10 cm in diameter)
- Severe (>10 cm in diameter or necrosis or exfoliative dermatitis for redness)
  (>10 cm in diameter or necrosis for induration)

This document is proprietary and is to be used only as authorized. No part is to be reproduced, disclosed to others, or quoted without prior written authorization from IDRI.

Oral temperature will be measured daily and recorded in the memory aid. General signs and symptoms will be limited to headache, joint pain (arthralgia), chills, anorexia (loss of appetite), fever, tiredness (fatigue), and generalized muscle pain (myalgia). Grading of none, mild, moderate, and severe will be used according to the following definitions:

- None
- Mild (no interference with daily activity)
- Moderate (some interference with daily activity)
- Severe (Significant interference, prevents daily activity)

'Normal' daily activities include those performed by most able people, such as dressing, bathing, walking, driving, housekeeping; they do not include unusually strenuous activities that may be 'normal' for certain individuals such as lifting heavy weights.

During the 60-minute observation period following the first study injection, subjects will be trained by the research staff on the proper completion of the memory aid and the schedule for completion. Subjects will also be trained in the proper procedure for taking oral temperature and will be asked to record their oral temperature each evening in the memory aid. Digital oral thermometers will be provided to all subjects for oral temperature readings. Subjects will also be provided with a measuring tool and trained on how to measure local redness (erythema) and hardening/swelling (induration).

Explicit choices for mild, moderate, and severe reactions will be indicated in the memory aid by the subjects, as defined above.

9.3 Serious Adverse Events (SAE)

9.3.1 Assessment of Seriousness

Seriousness refers to the outcome of an adverse event. Seriousness is determined by both the PI and the Sponsor Medical Monitor. If either PI or Sponsor Medical Monitor determines an event to be serious, it will be classified as such. If any of the following outcomes are present then the adverse event is serious:

- **It results in death** (i.e., the AE caused or led to the fatality). Serious does not describe an event that hypothetically might have caused death if it were more severe.

- **It was immediately life-threatening** (i.e., the AE placed the subject at immediate risk of dying). It does not refer to an event that hypothetically may have led to death if it were more severe.

- **It required inpatient hospitalization or prolonged hospitalization** beyond the expected length of stay. Hospitalizations for scheduled treatments and elective medical/surgical procedures related to a pre-existing condition that did not increase in severity or frequency following receipt of study injection, are not serious by this criterion. Hospitalization is defined as either a hospital admission or an emergency room visit for a period greater than 24 hours.

This document is proprietary and is to be used only as authorized. No part is to be reproduced, disclosed to others, or quoted without prior written authorization from IDRI.

- **It resulted in a persistent or significant disability/incapacity** (i.e., substantial reduction of the subject's ability to carry out activities of daily living).
- **It resulted in a congenital anomaly or birth defect** (i.e., an adverse finding in a child or fetus of a subject exposed to the study vaccine prior to conception or during pregnancy).
- **Other medically important conditions** that may not result in death, threaten life, or require hospitalization (i.e., the AE does not meet any of the above serious criteria) may be considered a serious adverse event when, based on appropriate medical judgment, they may jeopardize the subject and require medical or surgical intervention to prevent one of the serious outcomes listed in these criteria (e.g., allergic bronchospasm requiring intensive treatment in an emergency room or at home, blood dyscrasias or convulsions that do not result in hospitalization, or the development of drug dependency or drug abuse).

A serious adverse event is an adverse event meeting the outcome criteria for seriousness regardless of relationship to the study injection.

When an adverse event is judged related to an investigational product and is serious and unexpected, it is a SUSAR and subject to expedited reporting.

9.3.2 Procedures to be Followed in the Event of Abnormal Laboratory Test Values or Abnormal Clinical Findings

Investigation, treatment, follow-up, and medical referral of all adverse events will be determined by the investigator using his/her best medical judgment and according to local clinical practice guidelines.

Laboratory values that are abnormal based on the toxicity tables will be reported as an AE. In the event a subject experiences an AE or injection site reaction that is still present at the end of the subject's participation in the study, the subject must be followed until resolution of the event or until the event or abnormality resolves or stabilizes to the investigator's and/or sponsor's satisfaction. Follow-up procedures, evaluations, and outcomes will be recorded on the subject's eCRFs.

9.4 Reporting Procedures

The PI (or designee) must report immediately (within 24 hours) any occurrence of SAEs or other reportable events described in this section to IDRI Pharmacovigilance.

9.4.1 Serious Adverse Events

Serious AEs will be collected from the time of the first study drug administration until the final study visit. Serious AEs that are related to the investigational drug and continue beyond the normal collection period (i.e., are ongoing at the time a subject exits the study) will be followed until resolution or until stabilized with sequelae. Serious AEs that begin after the subject's

This document is proprietary and is to be used only as authorized. No part is to be reproduced, disclosed to others, or quoted without prior written authorization from IDRI.

participation in the study is complete, but that the Investigator considers to be related to study drug, may be reported at any time.

The Investigator or clinical site personnel should notify IDRI Pharmacovigilance of all SAEs, regardless of relationship to the investigational drug, within 24 hours of clinical site personnel becoming aware of the event. The Investigator will provide the initial notification by sending a completed “RAE Report Form,” which must include the Investigator’s assessment of the relationship of the event to investigational drug, and must be signed by the Investigator.

In addition, notification is sent by the clinical site to the IRB as required.

Follow-up information, or new information regarding an ongoing SAE, must be provided promptly to IDRI Pharmacovigilance.

All SAE reports should be sent to the following contacts:

In case of a serious adverse event, the Principal Investigator will send a report within 24 hours of notification to:

IDRI Pharmacovigilance
1616 Eastlake Ave. E, Suite 400
Seattle, WA 98102
E-mail: pvg@idri.org
Fax: 206-858-6098
Phone: 206-858-6039/6054

IDRI will ensure notification of all serious adverse events as soon as possible, but no later than 24 hours after learning of an event to DMID PVG below:

DMID Pharmacovigilance Group
Clinical Research Operations and Management Support (CROMS)
6500 Rock Spring Drive, Suite 650
Bethesda, MD 20817
SAE Hot Line: (800) 537-9979 (U.S.) or (301) 897-1709 (outside U.S.)
SAE FAX Phone Number: (800) 275-7619 (U.S.) or (301) 897-1710 (outside U.S.)
SAE Email Address: pvg@dmidcroms.com

IDRI is responsible for submitting the SAE to DMID as soon as possible, but no later than 24 hours after learning of an SAE.

The DMID Medical Monitor and the DMID Clinical Project Manager will be notified of the SAE by the DMID Pharmacovigilance Group. The DMID Medical Monitor will review and assess the SAE for potential impact on study subject safety and protocol conduct.

The Independent Safety Monitor will receive copies of all correspondence between the Sponsor and FDA for SAEs.

The sponsor is responsible for determining regulatory reporting requirements for SAEs.

This document is proprietary and is to be used only as authorized. No part is to be reproduced, disclosed to others, or quoted without prior written authorization from IDRI.

9.4.2 Regulatory Reporting

The sponsor will report all suspected unexpected serious adverse reactions (SUSARs) to the FDA within 7 or 15 days of receipt of the report of the event, depending on the nature of the SUSAR. In addition, all other SAEs and PIMMCs will be reported annually to the FDA in a dedicated tabulation by MedDRA coding term and relationship to study vaccine.

9.4.3 Reporting Other Adverse Events (if applicable)

The investigator must report the following events by scanning and emailing, or faxing, the appropriate form to the Sponsor Medical Monitor or designee within one business day of becoming aware of the event:

- Protocol violation affecting the safety of a participant or involving the study injection process (Reportable Adverse Event Form)
- Any event that, according to the protocol or in the opinion of the investigator, precludes further administration of the study injections (Reportable Adverse Event Form)
- Pregnancy (Pregnancy Alert Report Form)

9.4.4 Reporting of Pregnancy

If a participant becomes pregnant during the study, she will not receive any further study injections but should be encouraged to continue in the study for safety follow-up. Follow-up should continue for pregnancy outcome including premature terminations, and data are to be included in the safety reports.

The health status of the mother and child, the date of delivery, and the child's sex, birth weight, and parity should be reported to the Sponsor. Pregnancy will not be recorded as an adverse event. However, if the pregnancy results in a miscarriage or a planned termination, the event (spontaneous abortion or elective abortion) will be reported as an adverse event or serious adverse event per the investigator's judgment (e.g., if it was a medically important or life-threatening event that meets the definition of a serious adverse event).

A congenital anomaly or birth defect (i.e., an adverse finding in a child or fetus of a participant exposed to the study vaccine before conception or during pregnancy) must be reported as a serious adverse event.

9.5 Type and Duration of Follow-up of Subjects after Adverse Events

Adverse events will be considered resolved when the condition returns to normal or returns to the participant's baseline status as established on Study Day 0, or when the condition has stabilized with the expectation that it will remain chronic.

This document is proprietary and is to be used only as authorized. No part is to be reproduced, disclosed to others, or quoted without prior written authorization from IDRI.

The investigator will continue follow-up on adverse events, including laboratory abnormalities and solicited adverse events, until the event has resolved, is otherwise satisfactorily explained, or the participant completes the study.

Follow-up for serious adverse events must continue until resolution and the outcome reported, even if this extends beyond the serious adverse event reporting period (i.e., after the final study visit). For analysis purposes, the outcome for serious adverse events will be determined on the final study visit.

Outcome of all adverse events will be classified as one of the following:

- Resolved
- Resolved with sequelae
- Death

9.6 Halting Rules

9.6.1 Rules for Discontinuing Study injection in an Individual Subject

Administration of additional study injections will be discontinued for an individual if he/she has any of the following reactions:

- Changes in laboratory parameters that meet Grade 3 severity.
- High fever (oral temperature ≥ 102.2°F/39°C) within 1 week following study injection associated with constitutional symptoms and not attributable to another cause.
- Severe injection site reactogenicity that involves Grade 3 local pain, induration, or erythema.

9.6.2 Rules for Suspension of the Entire Study

The study will be immediately suspended (paused) and no additional study injections administered, pending review and discussion of all appropriate safety data by the SMC, <u>if one or more</u> subjects in any treatment group experience any of the following adverse events:

- Anaphylaxis or bronchospasm within 4 hours of study injection indicative of an immediate hypersensitivity reaction to the vaccine and not attributable to another cause.
- Any systemic rash, including but not limited to urticaria, generalized petechiae, or erythema multiforme, not attributable to another cause.
- Tissue necrosis at the study injection site.
- Any other serious adverse event deemed to be possibly, probably, or definitely relatable to the study injection by the PI or Sponsor Medical Monitor, based on close temporal relationship or other factors.

This document is proprietary and is to be used only as authorized. No part is to be reproduced, disclosed to others, or quoted without prior written authorization from IDRI.

The study will also be immediately suspended (paused) and no additional study injections administered pending review and discussion of all appropriate safety data by the SMC if three or more subjects experience the following AEs determined to be related to study injection:

- Changes in laboratory parameters that meet Grade 3 severity.
- High fever (oral temperature ≥ 102.2°F/39°C) within 1 week following study injection associated with constitutional symptoms and not attributable to another cause.
- Severe injection site reactogenicity that involves Grade 3 local pain, induration, or erythema.

Study suspension or termination will only apply to additional study injections. Subjects for whom their study injections are discontinued will remain in limited visit follow-up for safety evaluations and immunology blood draws.

9.7 Safety Oversight (ISM plus SMC)

9.7.1 Independent Safety Monitor (ISM)

The ISM is a physician with relevant expertise whose primary responsibility is to provide independent safety monitoring in a timely fashion. The ISM will review SAEs and other AEs as needed and provide an independent assessment to DMID. Saint Louis University will have an ISM with experience in infectious diseases or internal medicine.

9.7.2 Safety Monitoring Committee (SMC)

This clinical trial will utilize an SMC, which will be comprised of an independent group of experts with experience with early phase clinical research studies. The primary responsibility of the SMC is to monitor subject safety. The SMC is external to DMID and composed of at least three voting members. The SMC will consist of members with appropriate expertise to contribute to the interpretation of the data from this trial. Committee activities will be delineated in an SMC charter that will delineate membership, responsibilities, and the scope and frequency of data reviews. The SMC will operate on a conflict-free basis independently of the study team.

Subsequent to the Organizational Meeting, the SMC will review and discuss safety data at the following milestones:

- Ad hoc meeting will be conducted:
  - If study halting criteria are met as described in Section 9.6
  - At the request of DMID to review a potential safety concern identified by the PI, the Sponsor’s Medical Monitor, DMID MM or ISM
- Final review meeting: 6 to 8 months after clinical database lock to review the cumulative unblinded safety data for the study. The data will be provided in a standard summary format. The SMC may be asked to provide recommendations in response to questions posed by DMID.

This document is proprietary and is to be used only as authorized. No part is to be reproduced, disclosed to others, or quoted without prior written authorization from IDRI.

The SMC will have access to unblinded data during its closed session, if applicable. After its assessment, the SMC will recommend continuation, modification, or termination of the clinical trial. Additionally, data management alerts will be implemented and monitored to determine if any of the halting rules described in Section 9.6 are met.

If any of the halting rules are met following the first study injection, the study will not proceed with the remaining enrollment or study injections without a review by and recommendation from the SMC to proceed.

The DMID Medical Monitor and Clinical Project Manager will review the SMC recommendations and will document what actions DMID recommends be implemented by the study team. The Sponsor will receive the recommendations and will be responsible for responding to the SMC and DMID regarding any actions that were recommended and will not be implemented.

This document is proprietary and is to be used only as authorized. No part is to be reproduced, disclosed to others, or quoted without prior written authorization from IDRI.

10 CLINICAL MONITORING

10.1 Site Monitoring Plan

Site monitoring will be conducted to ensure that human subject protection, study procedures, laboratory procedures, study intervention administration, and data collection processes are of high quality and meet sponsor, ICH/GCP guidelines, and applicable regulatory requirements, and that the study is conducted in accordance with the protocol and sponsor’s standard operating procedures. The sponsor or its designee will conduct site-monitoring visits as specified in the monitoring plan.

Monitoring visits will include, but are not limited to, review of regulatory files, accountability records, eCRFs, informed consent forms, medical and laboratory reports, and protocol compliance. Study monitors will meet with investigators to discuss any problems and actions to be taken and document visit findings and discussions.

This document is proprietary and is to be used only as authorized. No part is to be reproduced, disclosed to others, or quoted without prior written authorization from IDRI.

11 STATISTICAL CONSIDERATIONS

11.1 Introduction

This study is a phase 1, randomized, double-blind clinical trial designed to evaluate the safety, tolerability, and immunogenicity of the single-vial and two-vial presentations of ID93 + GLA-SE. As such, the sample size of n=48 was determined by what is reasonable for a phase 1 trial and not based on statistical considerations of power, and will allow only preliminary safety and immunogenicity information relevant to progression to larger trials.

11.2 Study Population

The study population for this protocol is healthy males and non-pregnant females, ages 18-55 inclusive. The subjects must not have a history of infection with TB, must consent to all protocol requirements, and meet additional inclusion and exclusion criteria specified in Section 5.

11.2.1 Intent to Treat Population

The Intent-to-Treat (ITT) Population will include all subjects randomized to one of the treatment groups, classified according to the randomization assignment.

11.2.2 Per Protocol Population

The Per Protocol Population will include all eligible subjects randomized to one of the treatment groups and who received both assigned study injections. Subjects will be classified according to the treatment received. Analysis of immunogenicity endpoints occurring after the second study injection will be performed on this population.

11.2.3 Safety Population

The Safety Population will consist of all subjects who have received at least one dose of study injection and for whom any data on safety are available. Subjects will be classified according to the treatment received. The primary safety analysis will be done on this population.

11.2.4 Immunogenicity Population

The Immunogenicity Population will include all eligible subjects who have received at least one study injection, for whom data concerning post-baseline immunogenicity endpoint measures are available, and major protocol deviations are absent. The post-baseline immunogenicity endpoints may include humoral or cellular responses measured at the protocol-specified time points, or at the time of early termination. Subjects will be classified according to the treatment

This document is proprietary and is to be used only as authorized. No part is to be reproduced, disclosed to others, or quoted without prior written authorization from IDRI.

received. Immunogenicity analysis will be done using this population or the Per Protocol Population, as appropriate.

11.3 Sample Size Considerations

Sample size for this study will allow only preliminary safety information relevant to progression to larger trials. The study is not designed to test a formal hypothesis.

Sample Size Calculations for Safety: The goal of the safety evaluation for this trial is to identify safety concerns associated with the study injections. The probability of detecting at least one event for a number of true underlying event rates for three potential sample sizes is listed in Table 2.

Table 2: Probability of observing at least one event for several true event rates and the event rates which cannot be ruled out if no events are observed.

True Event Rate	Probability of observing 1 or more events		
	N=10	N=20	N=30
0.05	40%	64%	79%
0.10	65%	88%	96%
0.15	80%	96%	99%
0.25	94%	100%	100%

11.4 Planned Interim Analyses (if applicable)

There is no planned interim analysis. Safety data will be reviewed by the SMC during the study if necessary as outlined in Section 9.7.

11.5 Final Analysis Plan

The final analysis will be used to evaluate the safety, tolerability, and immunogenicity of two IM doses of ID93 + GLA-SE in the single-vial or two-vial presentations at study Days 0 and 56.

A detailed Statistical Analysis Plan will be developed prior to study completion and database lock. The analysis will consist of baseline and demographic data, safety data, immunogenicity data, and laboratory data. Data will be summarized with standard descriptive statistics and provided in tables, listings, and figures as appropriate.

11.5.1 Analysis of Demographics and Disposition

Summaries and descriptive statistics of demographic and baseline characteristics will be provided for the safety population.

The following subject disposition information will be summarized for each treatment group:

This document is proprietary and is to be used only as authorized. No part is to be reproduced, disclosed to others, or quoted without prior written authorization from IDRI.

- Number of subjects enrolled
- Stratified by enrollment group:
  - Number (%) of subjects receiving one or two study injections
  - Number (%) of subjects who discontinued the study and reasons for discontinuation
  - Number (%) of subjects in the per-protocol population and reasons for those excluded
  - Number (%) of subjects with at least one protocol violation

Protocol violations will be identified by the Medical Monitor (or designee) for the trial.

11.5.2 Analysis of Safety

Summaries of the safety endpoints will be completed for the safety population. Safety endpoints include solicited adverse events, unsolicited adverse events, changes in laboratory values, and changes in vital signs. All solicited and unsolicited adverse events will be recorded by the Investigator in the AE eCRF and all adverse event terms will be coded using MedDRA preferred term and system organ class.

11.5.2.1 Solicited Adverse Events

The severity rating (Grade 1 to 3) assigned to each solicited adverse event that is reported based on in-clinic diagnosis and on review of the subject memory aids will be summarized by the 7-day injection interval following each injection and by treatment group. The worst value (i.e., the maximum severity) for each sign/symptom will also be summarized by injection interval and treatment group.

11.5.2.2 Unsolicited Adverse Events

All unsolicited adverse event terms will be coded using MedDRA. Summaries of the number (%) of subjects in each treatment group with at least one adverse event, classified according to MedDRA preferred term and system organ class, will be provided for:

- All adverse events
- Related adverse events
- Serious adverse events
- PIMMCs

Related adverse events are defined as adverse events that are possibly, probably, or definitely related to study injection, as assessed by the study PI.

The following additional summaries of unsolicited adverse events will also be provided:

- Incidence of adverse events after the first and second injections
- Incidence of adverse events by severity of event

This document is proprietary and is to be used only as authorized. No part is to be reproduced, disclosed to others, or quoted without prior written authorization from IDRI.

- Incidence of adverse events by relationship to study injection

The adverse event summaries described in the previous paragraphs will also be provided based on all (i.e., unsolicited and solicited) adverse events.

11.5.2.3 Laboratory Data

Summaries of the actual value and change from baseline for all laboratory tests will be provided for each study visit. A tabulated summary of laboratory abnormalities by toxicity grade will also be provided.

11.5.2.4 Vital Signs

Summaries of the actual value and change from baseline for all vital signs will be provided for each study visit.

11.5.2.5 Missing Data

Missing data will be recorded as they are, and there will be no imputation of missing values.

11.5.3 Immunology Analyses

Immunogenicity will be assessed from blood samples obtained on Days 0, 7, 14, 56, 63, 70, 84, and 224. Secondary immunogenicity evaluations will include quantification of T cell cytokine responses from stimulated PBMCs and serum IgG antibody responses to ID93. Exploratory analyses will include measurement of functionality of immune responses through the whole blood mycobacterial growth inhibition assay (MGIA), measurement of humoral responses in mucosal secretions (nasal swabs and tears), quantitation of IgA and IgG secreting B cells and memory B cells, and measurement of the frequencies of peripheral T follicular helper cells, memory B cells, and T cell homing markers.

Immune responses for each treatment group will be evaluated by calculating the response rate and magnitude of response to ID93 at all timepoints for each immunologic endpoint. Comparisons of response rates for IgG and cytokine responses across treatment groups will be made using n x m Fisher's exact tests. Pairwise comparisons will be done using 2 x 2 Fisher's exact tests with multiplicity adjustment to control the family-wise error rate (FWER).

The magnitude of IgG and cytokine responses will be compared across treatment groups using ANOVA when the distribution is Gaussian and Kruskal-Wallis when the distribution is non-Gaussian. When ANOVA is used and a difference is observed across the treatment groups, pairwise comparisons will be made using t-tests with adjustment to control the FWER. If Kruskal-Wallis is used, Wilcoxon rank-sum tests will be used.

Percentages of CD4 and CD8 T cells producing selected cytokines will be summarized by time point and compared across and between treatment groups.

This document is proprietary and is to be used only as authorized. No part is to be reproduced, disclosed to others, or quoted without prior written authorization from IDRI.

12 SOURCE DOCUMENTS AND ACCESS TO SOURCE DATA/DOCUMENTS

The investigator is responsible for ensuring the accuracy, completeness, legibility, and timeliness of the data reported.

The Investigator will allow representatives of IDRI (or their designee) to periodically audit, at mutually convenient times during and after the study, all eCRFs and corresponding source documents for each subject. It is important that the Investigator and/or other staff are available at these visits. The monitoring visits provide IDRI with the opportunity to evaluate the progress of the study, to verify the accuracy and completeness of eCRFs, to resolve any inconsistencies in the study records, as well as assuring that all protocol requirements, applicable regulations, and Investigator's obligations are being fulfilled. The Investigator must maintain source documents such as laboratory and consultation reports, history and physical examination reports, etc., for possible review.

Telephone contact will be made as necessary during the data collection period and during the data and report writing periods. Data reported in the eCRF derived from the data collection forms should be consistent with the source documents or the discrepancies should be explained.

The sponsor will provide guidance to investigators on making corrections to the data collection forms and eCRFs.

This document is proprietary and is to be used only as authorized. No part is to be reproduced, disclosed to others, or quoted without prior written authorization from IDRI.

13 QUALITY CONTROL AND QUALITY ASSURANCE

Following a written sponsor and DMID-accepted site quality management plan, the investigational site is responsible for conducting routine quality assurance and quality control activities to internally monitor study progress and protocol compliance. The PI will provide direct access to all trial-related site source data/documents, and reports for the purpose of monitoring and auditing by the sponsor, and inspection by local and regulatory authorities. The PI will ensure all study personnel are appropriately trained and applicable documentation is maintained on site.

Sponsor-designated clinical monitors will verify that the clinical trial is conducted and data are generated, documented (recorded), and reported in compliance with the protocol, ICH/GCP guidelines, and the applicable regulatory requirements. Clinical monitoring reports will be submitted to DMID.

DF/Net Research will implement quality control procedures beginning with the data entry system and generate data quality control checks that will be run on the database. Any missing data or data anomalies will be communicated to the site for clarification and resolution.

This document is proprietary and is to be used only as authorized. No part is to be reproduced, disclosed to others, or quoted without prior written authorization from IDRI.

14 ETHICS/PROTECTION OF HUMAN SUBJECTS

14.1 Ethical Standard

The experimental protocol for this study has been designed in accordance with the E6 Good Clinical Practice: Consolidated Guidance (ICH), and the U.S. Code of Federal Regulations governing the protection of human subjects (21 CFR 50). The review of this protocol by the Institutional Review Board (IRB) and the performance of all aspects of the study, including the methods used for obtaining informed consent, must also be in accordance with the regulations described in Title 21 Code of Federal Regulations (CFR) Part 50 Protection of Human Subjects and Part 56 Institutional Review Boards. The sponsor and the investigator's institution will hold a current Federal Wide Assurance issued by Office for Human Research Protections for federally funded research.

14.2 Institutional Review Board

Prior to enrollment of subjects into this trial, the approved protocol and the informed consent form will be reviewed and approved by the appropriate IRB. The Investigator will be responsible for preparing documents for submission to the relevant IRB and obtaining written approval for this study. The responsible official for the IRB will sign the IRB letter of approval of the protocol prior to the start of this trial and a copy will be provided to the sponsor and DMID. Notification of the IRB's composition, or the IRB's Federal Wide Assurance number, will be provided to DMID. Should amendments to the protocol be required, the amendments will be written by the sponsor and provided to the investigator for submission to the IRB.

14.3 Informed Consent Process

All subjects must sign an informed consent form that complies with the requirements of both 21 CFR 50 and 45 CFR 46.

Prior to participation in the trial, subjects will receive a comprehensive explanation of the proposed vaccine, including the nature and risks of the trial, alternate therapies, any known AEs, the investigational status of the components, and the other elements that are part of obtaining proper informed consent. Subjects will also receive a detailed explanation of the proposed use and disclosure of their protected health information, including specifically their serum samples. Subjects will be allowed sufficient time to consider participation in the trial, after having the nature and risks of the trial explained to them. The consent form must not include any exculpatory statements.

The sponsor will provide the investigator, in writing, any new information that significantly affects risk related to a subject's receipt of the investigational product. This new information will be communicated by the investigator to subjects who consent to participate in the trial in accordance

This document is proprietary and is to be used only as authorized. No part is to be reproduced, disclosed to others, or quoted without prior written authorization from IDRI.

with IRB requirements. The informed consent document will be updated and subjects will be re-consented, if necessary.

Site staff may utilize IRB-approved recruitment methods prior to the subject consenting; however, before any protocol-specific procedures are performed to determine protocol eligibility an informed consent form must be signed. Subjects will be given a copy of all consent forms that they sign.

By signing the informed consent form, the subject agrees to complete all evaluations required by the trial, unless the subject withdraws voluntarily or is terminated from the trial for any reason.

14.4 Exclusion of Women, Minorities, and Children (Special Populations)

This study will be inclusive of all healthy adults who meet the inclusion/exclusion criteria, regardless of religion, sex, or ethnic background. Only individuals who are 18 to 55 years old, inclusive, will be included at this time. Children are excluded for safety reasons.

14.5 Subject Confidentiality

Subjects will have code numbers and will not be identified by name. Subject confidentiality is strictly held in trust by the participating investigators, their staff, the sponsor(s), and their agents. This confidentiality extends to genetic and biological sample tests, in addition to the clinical information relating to participating subjects.

The study protocol, documentation, data, and all other information generated will be held in strict confidence. Documented evidence that shows that a potential investigator is aware of and agrees to the confidential nature of the information related to the study must be obtained by means of a confidentiality agreement.

All information provided by the sponsor and all data and information generated by the site as part of the study (other than a subject's medical records) will be kept confidential by the investigator and other site staff. This information and data will not be used by the investigator or other site personnel for any purpose other than conducting the study. These restrictions do not apply to: (1) information that becomes publicly available through no fault of the investigator or site staff; (2) information that it is necessary to disclose in confidence to an IRB solely for the evaluation of the study; (3) information that it is necessary to disclose in order to provide appropriate medical care to a study subject; or (4) study results that may be published as described in Section 16. If a written contract for the conduct of the study that includes confidentiality provisions inconsistent with this statement is executed, that contract's confidentiality provisions shall apply rather than this statement.

The study monitor or other authorized representatives of the sponsor and/or the FDA may inspect all documents and records required to be maintained by the investigator. This includes, but is not limited to, medical records (office, clinic, or hospital) and pharmacy records for the subjects in this study. Clinical study sites will permit access to such records.

This document is proprietary and is to be used only as authorized. No part is to be reproduced, disclosed to others, or quoted without prior written authorization from IDRI.

To protect privacy, we have received a Certificate of Confidentiality. With this Certificate, the researchers cannot be forced to release information that may identify the research subject, even by a court subpoena, in any federal, state, or local civil, criminal, administrative, legislative, or other proceedings. The researchers will use the Certificate to resist any demands for information that would identify the subject, except as explained below.

The Certificate cannot be used to resist a demand for information from personnel of the United States Government that is used for auditing or evaluation of federally funded projects, like this study, or for information that must be released in order to meet the requirements of the Federal Food and Drug Administration (FDA).

A Certificate of Confidentiality does not prevent the subject from voluntarily releasing information about themselves or their involvement in this research. If any person or agency obtains a written consent to receive research information, then the researchers may not use the Certificate to withhold that information.

The Certificate of Confidentiality does not prevent the researchers from reporting without the subject's consent, information that would identify the subject as a participant in the research project regarding matters that must be legally reported including: child and elder abuse, sexual abuse, or wanting to harm themselves or others.

14.6 Study Discontinuation

If the study is discontinued, enrolled subjects will continue to be followed for safety assessments. No further study injections will be administered.

14.7 Future Use of Stored Specimens

Subjects will be asked for permission to keep any remaining specimens for possible use in future research studies, such as testing for antibodies against viruses or bacteria. Samples will be stored at IDRI for up to 10 years. Samples may be shared with other investigators at other institutions. The samples will not be sold or used directly for production of any commercial product. Each sample will be encoded (labeled) *only* with a barcode and a unique tracking number to protect subject's confidentiality.

Additional samples will be taken for the sole purpose of banking serum for auto-immune testing, if indicated (i.e., if a participant has a PIMMC). These samples will not be used for other purposes and will be discarded after 10 years.

There are no benefits to subjects in the collection, storage, and subsequent research use of specimens. Reports about future research done with a subject's samples will NOT be kept in their health records. Subjects can decide if they want their samples to be used for future research or have their samples destroyed at the end of the study. A subject's decision can be changed at any time prior to the end of the study by notifying the study doctors or nurses in writing. However, if a

This document is proprietary and is to be used only as authorized. No part is to be reproduced, disclosed to others, or quoted without prior written authorization from IDRI.

subject consents to future use and some of their blood has already been used for research purposes, the information from that research may still be used.

14.8 Disclosure of Individual Research Information

IDRI does not intend to provide each participant with a summary of study results or their treatment assignment.

This document is proprietary and is to be used only as authorized. No part is to be reproduced, disclosed to others, or quoted without prior written authorization from IDRI.

15 DATA HANDLING AND RECORD KEEPING

The investigator is responsible to ensure the accuracy, completeness, legibility, and timeliness of the data reported. Data reported in the eCRF derived from source documents should be consistent with the source documents or the discrepancies should be explained. IDRI and/or it designee will provide guidance to investigators on making corrections to the source documents and eCRF.

15.1 Data Management Responsibilities

All source documents and laboratory reports must be reviewed by the clinical team and data entry staff, who will ensure that they are accurate and complete. Adverse events must be graded, assessed for severity and causality, and reviewed by the site PI or designee.

Data collection is the responsibility of the clinical trial staff at the site under the supervision of the site PI. During the study, the investigator must maintain complete and accurate documentation for the study.

DF/Net Research will be responsible for data management, quality review, analysis, and reporting of the study data.

15.2 Data Capture Methods

Clinical data (including AEs, concomitant medications, and reactogenicity data) and clinical laboratory data will be entered into a 21 CFR Part 11-compliant data entry system provided by DF/Net Research. The data system includes password protection and internal quality checks, such as automatic range checks, to identify data that appear inconsistent, incomplete, or inaccurate. Clinical data will be entered directly from the source documents.

15.3 Types of Data

Data for this study will include safety, immunologic laboratory, and outcome measures (e.g., reactogenicity, immunogenicity).

15.4 Timing/Reports

Safety data will be reviewed by the SMC per the SMC charter for this study. The SMC will make a recommendation at that time as to the advisability of proceeding with study injections. Interim statistical reports may be generated as deemed necessary and appropriate by the sponsor and DMID.

This document is proprietary and is to be used only as authorized. No part is to be reproduced, disclosed to others, or quoted without prior written authorization from IDRI.

15.5 Study Records Retention

Each participating site will maintain appropriate medical and research records for this trial, in compliance with ICH E6, Section 4.9 and regulatory and institutional requirements for the protection of confidentiality of subjects. Each site will permit authorized representatives of IDRI, its designees, DMID, and appropriate regulatory agencies to examine (and when required by applicable law, to copy) clinical records for the purposes of quality assurance reviews, audits, monitoring, and evaluation of the study safety and progress. These representatives will be permitted access to all source data, which include, but are not limited to, hospital records, clinical and office charts, laboratory notes, memoranda, evaluation checklists, pharmacy dispensing records, recorded data from automated instruments, copies or transcriptions certified after verification as being accurate and complete, microfiches, photographic negatives, microfilm or magnetic media, x-rays, and subject files and records kept at the pharmacy, at the laboratories, and medico-technical departments involved in the clinical trial. Data collection forms used as source documents will be derived from the eCRFs and be provided by DF/Net Research.

Records and documents pertaining to the conduct of this study, including eCRFs, data collection forms, other source documents, consent forms, laboratory test results, and medication inventory records, must be retained by the investigator for at least 2 years following submission of a Biologics License Application or until IDRI authorizes transfer or destruction of study records. No study records will be destroyed without prior authorization from IDRI.

15.6 Protocol Deviations

A protocol deviation is any noncompliance with the clinical trial protocol, Good Clinical Practice (GCP), or SSP Manual requirements. The noncompliance may be on the part of either the subject, the investigator, or the study site staff. As a result of deviations, corrective actions are to be developed by the site and implemented promptly. All deviations must be promptly reported. All deviations from the protocol must be addressed in study subject source documents. Protocol deviations must be sent to the local IRB per their guidelines. The site PI/study staff is responsible for knowing and adhering to their IRB requirements.

This document is proprietary and is to be used only as authorized. No part is to be reproduced, disclosed to others, or quoted without prior written authorization from IDRI.

16 PUBLICATION POLICY

All information contained in this clinical study protocol and accompanying documents is confidential. All data collected during the course of this study are confidential and considered the property of IDRI. The Investigators agree to use this information and data only in accomplishing this study and will not use it for other purposes without the written permission of IDRI. IDRI encourages publication in peer-reviewed medical journals and will not unduly withhold permission to publish. However, all proposed publications, papers, abstracts, or written materials related to the study, or an outline or poster of any oral presentation, shall be submitted to and coordinated by IDRI for review at least thirty (30) days in advance of the submission to assure that no proprietary information is presented and that authorship is fairly represented.

Within thirty (30) days after receipt of such materials, IDRI may request in writing that the proposed publication or presentation be delayed or modified, specifying in reasonable detail the reasons for the request. If IDRI objects to a proposed publication or presentation on the basis that it would disclose confidential information, the Investigator shall remove the objectionable information from such proposed publication or presentation. If the parties disagree concerning whether certain information should be deleted or modified, the parties agree to meet for the purpose of making good faith efforts to discuss and resolve any such issues or disagreements. IDRI will work with the Institution in a collaborative manner to make certain that the clinical study results contained within such presentation or publication (whether positive or negative) are accurate.

Following completion of the study, the investigator or sponsor is expected to publish the results of this research in a scientific journal. The International Committee of Medical Journal Editors (ICMJE) member journals have adopted a trials-registration policy as a condition for publication. This policy requires that all clinical trials be registered in a public trials registry such as ClinicalTrials.gov, which is sponsored by the National Library of Medicine. Other biomedical journals are considering adopting similar policies. It is the responsibility of the Sponsor to register this trial in an acceptable registry. Any clinical trial starting enrollment after 01 July 2005 must be registered on or before patient enrollment. For trials that began enrollment prior to this date, the ICMJE member journals will require registration by 13 September 2005, before considering the results of the trial for publication.

The ICMJE defines a clinical trial as any research project that prospectively assigns human subjects to intervention or comparison groups to study the cause-and-effect relationship between a medical intervention and a health outcome. Studies designed for other purposes, such as to study pharmacokinetics or major toxicity (e.g., Phase I trials), would be exempt from this policy.

By signing the study protocol, the PI agrees that the results of the study may be used for national and international registration, publication, and information for medical and pharmaceutical professionals by the Sponsor. If necessary, the authorities will be notified of the PI's name, address, qualifications, and extent of involvement.

This document is proprietary and is to be used only as authorized. No part is to be reproduced, disclosed to others, or quoted without prior written authorization from IDRI.

If the PI or site is to be included as an author of a publication manuscript prepared by the Sponsor, the Sponsor will allow the PI or site 60 days for full review of the manuscript before publication.

As of January 2018, all clinical trials supported by the NIH must be registered on ClinicalTrials.gov, no later than 21 days after the enrollment of the first subject. Results of all clinical trials supported by the NIH, generally, need to be submitted no later than 12 months following the primary completion date. A delay of up to 2 years is available for trials that meet certain criteria and have applied for certification of delayed posting.

As part of the result posting a copy of this protocol (and its amendments) and a copy of the Statistical Analysis Plan will be posted on ClincialTrials.gov.

For this trial the responsible party is IDRI, which will register the trial and post results.

The responsible party does not plan to request certification of delayed posting.

Refer to:

- Public Law 110-85, Section 801, Clinical Trial Databases
- 42CFR11
- NIH NOT-OD-16-149

This document is proprietary and is to be used only as authorized. No part is to be reproduced, disclosed to others, or quoted without prior written authorization from IDRI.

17 LITERATURE REFERENCES

1. Ottenhoff TH, Kaufmann SH. Vaccines against tuberculosis: where are we and where do we need to go? PLoS pathogens **2012**; 8:e1002607.
2. Flynn JL, Goldstein MM, Chan J, et al. Tumor necrosis factor-alpha is required in the protective immune response against Mycobacterium tuberculosis in mice. Immunity **1995**; 2:561-72.
3. Flynn JL, Chan J. Immunology of tuberculosis. Annu Rev Immunol **2001**; 19:93-129.
4. Gardam MA, Keystone EC, Menzies R, et al. Anti-tumour necrosis factor agents and tuberculosis risk: mechanisms of action and clinical management. Lancet Infect Dis **2003**; 3:148-55.
5. Hoft DF. Tuberculosis vaccine development: goals, immunological design, and evaluation. Lancet **2008**; 372:164-75.
6. Colditz GA, Brewer TF, Berkey CS, et al. Efficacy of BCG vaccine in the prevention of tuberculosis. Meta-analysis of the published literature. JAMA **1994**; 271:698-702.
7. Rodrigues LC, Diwan VK, Wheeler JG. Protective effect of BCG against tuberculous meningitis and miliary tuberculosis: a meta-analysis. Int J Epidemiol **1993**; 22:1154-8.
8. Trunz BB, Fine P, Dye C. Effect of BCG vaccination on childhood tuberculous meningitis and miliary tuberculosis worldwide: a meta-analysis and assessment of cost-effectiveness. Lancet **2006**; 367:1173-80.
9. Kaufmann SH, Hussey G, Lambert PH. New vaccines for tuberculosis. Lancet **2010**; 375:2110-9.
10. McShane H. Tuberculosis vaccines: beyond bacille Calmette-Guerin. Philos Trans R Soc Lond B Biol Sci **2011**; 366:2782-9.
11. Weiner J, 3rd, Kaufmann SH. Recent advances towards tuberculosis control: vaccines and biomarkers. Journal of internal medicine **2014**; 275:467-80.
12. Bertholet S, Ireton GC, Ordway DJ, et al. A defined tuberculosis vaccine candidate boosts BCG and protects against multidrug-resistant Mycobacterium tuberculosis. Science translational medicine **2010**; 2:53ra74.
13. Baldwin SL, Bertholet S, Reese VA, Ching LK, Reed SG, Coler RN. The importance of adjuvant formulation in the development of a tuberculosis vaccine. J Immunol **2012**; 188:2189-97.

This document is proprietary and is to be used only as authorized. No part is to be reproduced, disclosed to others, or quoted without prior written authorization from IDRI.

Appendix 1: Schedule of Study Visits and Procedures

	**Screen**	**Schedule for Study Injection Phase (Days with allowable visit window)**						**Follow-Up** visit	visit	phone	**ET[1] visit**
	**Day -30 to Day 0**	**0**	**7 (±1)**	**14 (±3)**	**56 (±3)**	**63 (±1)**	**70 (±3)**	**84 (±5)**	**224 (±10)**	**421 (±14)**	
Informed consent	X										
Inclusion/exclusion criteria	X	X			X						
Medical history	X										
Concomitant medications	X	X	X		X	X		X	(X)[6]	(X)[6]	X
Physical exam	X										
Targeted physical exam[5]		X	X		X	X		X	X		X
Vital signs	X	X[2]	X		X[2]	X		X			X
Safety labs	X		X			X					
HIV, HCV, HBsAg	X										
Urinalysis (protein, glucose)	X										
Serum pregnancy test or FSH[3]	X										
Urine pregnancy test		X			X						
Visual acuity	X										
QuantiFERON®-TB Gold	X										
Nasal swab and tear collection		X					X		X		(X)[7]
Immunology blood[4]		X	X	X	X	X	X	X	X		(X)[7]
Study injection		**X**			**X**						
Injection site checks		X	X		X	X					
Dispense memory aid		X			X						
Review memory aid			X			X					
Adverse events	X	X	X		X	X		X			X
SAE/PIMMC	X	X	X		X	X		X	X	X	X

[1] ET = Early termination
[2] Vital signs are performed prior to and at 60 minutes post study injection.
[3] Serum pregnancy test for females of childbearing potential; FSH for post-menopausal females
[4] See Appendix 2 for volumes of immunology blood.
[5] If indicated, based on review of symptoms
[6] Concomitant meds to be collected only if used for treatment of an SAE or PIMMC.
[7] If indicated

This document is proprietary and is to be used only as authorized. No part is to be reproduced, disclosed to others, or quoted without prior written authorization from IDRI.

Appendix 2: Schedule of Blood Draws and Volumes

	Screen	Day 0	Day 7 (±1)	Day 14 (±3)	Day 56 (±3)	Day 63 (±1)	Day 70 (±3)	Day 84 (±5)	Day 224 (±10)
Serum chemistry and hematology	12.5		12.5			12.5			
HIV 1/2, HBsAg, HCV	12								
QuantiFERON®-TB Gold	3								
Serum IgG		6*		4	4		4	4	4
MGIA		4					4		4
PBMC**		64	24	64	64	24	64	64	64
**Blood volume per visit**	27.5	74	36.5	68	68	36.5	72	68	72
**Cumulative total volume**	**27.5**	**101.5**	**138**	**206**	**274**	**310.5**	**382.5**	**450.5**	**522.5**

Note: blood volumes are approximate and may be adjusted for clinical site requirements
* Includes 2 mL for serum to be banked for autoimmune testing, if indicated
** PBMC utilized in ICS and ELISpot will be left unstimulated or stimulated with ID93 protein or overlapping peptides pools encompassing the individual vaccine antigens (Rv1813, Rv2608, Rv3619, Rv3620), or a positive control (SEB). PBMC used in B cell ELISpot will be left unstimulated or stimulated with ID93 protein, SEB, or polyclonal stimulation reagents.

This document is proprietary and is to be used only as authorized. No part is to be reproduced, disclosed to others, or quoted without prior written authorization from IDRI.

Appendix 3: Grading Scale for Clinical Laboratory Values

Parameter	Grade for Abnormal Results (Value or Change from Reference) [1, 2]		
	**Mild (Grade 1)**	**Moderate (Grade 2)**	**Severe (Grade 3)**
Sodium (mmol/L) [2]	< LLN - 130<br>> ULN - 150	125 - 129<br>151 - 154	< 125<br>> 154
Potassium (mmol/L) [2]	< LLN - 3.0<br>> ULN - 6.0	2.5 - 2.9<br>6.1 - 6.5	< 2.5<br>> 6.5
Creatinine (mg/dL)	> ULN - 1.7	1.8 - 2.0	> 2.0
Total bilirubin (mg/dL)	> ULN - 1.5 x ULN*<br>> ULN - 1.25 x ULN**	> 1.5 - 2.0 x ULN*<br>> 1.25 - 1.5 x ULN**	> 2.0 x ULN*<br>> 1.5 x ULN**
ALT (SGPT) (U/L)	> ULN - 2.5 x ULN	> 2.5 - 5.0 x ULN	> 5.0 ULN
AST (SGOT) (U/L)	> ULN - 2.5 x ULN	> 2.5 - 5.0 x ULN	> 5.0 ULN
Alkaline phosphatase (U/L)	> ULN - 2.0 x ULN	> 2.0 - 3.0 x ULN	> 3.0 ULN
Random glucose (mg/dL)	Not followed after baseline screening		
WBC<br>(K/µL, K/mm^3 or 10^9/L)	> ULN - 15.0<br>2.5 - < LLN	> 15.0 - 20.0<br>≥ 1.5 - < 2.5	> 20.0<br>< 1.5
Hemoglobin (g/dL)	M: 12.5 - < LLN<br>F: 11 - < LLN	M: 10.5 - 12.4<br>F: 9.5 - 10.9	M: < 10.5<br>F: < 9.5
Platelet count<br>(K/µL, K/mm^3 or 10^9/L)	100 - < LLN	50 - 99	< 50

ULN=upper limit of normal; LLN=lower limit of normal.

[1] FDA Guidance for Industry Toxicity Grading Scale for Healthy Adults Enrolled in Preventive Vaccine Clinical Trials, 2007.

[2] DAIDS Toxicity Grading scale for electrolytes.

* Tox grading for total bilirubin when liver transaminases are normal.

** Tox grading for total bilirubin when accompanied by increased transaminases.

This document is proprietary and is to be used only as authorized. No part is to be reproduced, disclosed to others, or quoted without prior written authorization from IDRI.

Appendix 4: Grading Scale for Local Injection Site Reactions

Local Finding	Grade 0 (None)	Grade 1 (Mild)	Grade 2 (Moderate)	Grade 3 (Severe)
Pain	None	Does not interfere with activity	Repeated use of non-narcotic pain reliever > 24 hours or interferes with activity	Any use of narcotic pain reliever or prevents daily activity
Erythema/Redness	< 2.5 cm	2.5 – 5.0 cm	5.1 – 10 cm	> 10 cm or necrosis or exfoliative dermatitis
Induration/Swelling	< 2.5 cm	2.5 – 5.0 cm	5.1 – 10 cm	> 10 cm or necrosis

Note: Local injection site reaction grading should not be confused with general AE grading.

This document is proprietary and is to be used only as authorized. No part is to be reproduced, disclosed to others, or quoted without prior written authorization from IDRI.

Appendix 5: Grading Scale for Determining the Severity of Clinical AEs

VITAL SIGNS*	Mild (Grade 1)	Moderate (Grade 2)	Severe (Grade 3)
Fever: oral (˚C)**<br>(˚F)**	38.0 - 38.4<br>100.4 - 101.2	38.5 - 38.9<br>101.3 - 102.1	≥ 39.0<br>≥ 102.2
Tachycardia - bpm	101 - 115	116 - 130	> 130
Bradycardia – bpm*	50 – 54<br>or >10 bpm less than baseline, if baseline < 50	45 – 49<br>or >15 bpm less than baseline, if baseline < 50	< 45<br>or > 20 bpm less than baseline, if baseline < 50
Hypertension (systolic) mm Hg	141 - 150	151 - 155	> 155
Hypertension (diastolic) mm Hg	91 - 95	96 - 100	> 100
Hypotension (systolic) mm Hg	85 - 89	80 - 84	< 80
Hypotension (diastolic) mm Hg	50 - 54	45 - 49	< 45
Respiratory Rate – breath per minute	17 - 20	21 - 25	> 25

* Subjects should be at rest for at least 5 minutes prior to vital sign measurements
** Oral temperature; no recent hot or cold beverages or smoking. Note: A fever can be considered not related to the study product if an alternative etiology can be documented and it is confirmed to be not related to the study product by the Independent Safety Monitor at the site.
*** When resting heart rate is between 60-100 beats per minute. Use clinical judgment when characterizing bradycardia among some healthy subject populations, for example conditioned athletes.

Systemic (General)	Mild (Grade 1)	Moderate (Grade 2)	Severe (Grade 3)
Headache	Does not interfere with activity	Repeated use of non-narcotic pain reliever >24 hours or interferes with activity	Significant; any use of narcotic pain reliever or prevents daily activity
Fatigue	Does not interfere with activity	Interferes with activity	Significant; prevents daily activity
Myalgia (generalized muscle pain)	Does not interfere with activity	Interferes with activity	Significant; prevents daily activity
Arthralgia (joint pain)	Does not interfere with activity	Interferes with activity	Prevents daily activity
Chills	Does not interfere with activity	Interferes with activity	Prevents daily activity
Anorexia (loss of appetite)	Does not interfere with activity	Interferes with activity	Prevents daily activity
Nausea/Vomiting	Does not interfere with activity or 1 - 2 episode in 24 hours	Interferes with activity or >2 episodes in 24 hours	Prevents daily activity or requires outpatient IV hydration
Diarrhea	2-3 loose stools or < 400 gms/24 h	4-5 stools or 400-800 gms/24 hours	6 or more watery stools or >800 gms/24 hours or requires outpatient IV hydration
Illness or clinical adverse event (as defined according to applicable regulations)	Does not interfere with activity.	Interferes with activity not requiring medical intervention	Prevents daily activity and requires medical intervention

This document is proprietary and is to be used only as authorized. No part is to be reproduced, disclosed to others, or quoted without prior written authorization from IDRI.

Appendix 6: List of PIMMCs

This list is current as of November 2017. It is provided by FDA/CBER and is subject to periodic update. Any updates will be implemented via a Note to File.

Gastrointestinal disorders	Liver disorders	Metabolic diseases
• Celiac disease<br>• Crohn's disease<br>• Ulcerative colitis<br>• Ulcerative proctitis	• Autoimmune cholangitis<br>• Autoimmune hepatitis<br>• Primary biliary cirrhosis<br>• Primary sclerosing cholangitis	• Addison's disease<br>• Autoimmune thyroiditis (including Hashimoto thyroiditis)<br>• Diabetes mellitus type I<br>• Grave's or Basedow's disease
**Neuroinflammatory disorders**	**Musculoskeletal disorders**	**Skin disorders**
• Acute disseminated encephalomyelitis, including site specific variants (eg, non-infectious encephalitis, encephalomyelitis, myelitis, radiculomyelitis)<br>• Cranial nerve disorders, included paralyses/paresis (eg, Bell's palsy)<br>• Guillain-Barré syndrome, including Miller Fisher syndrome and other variants<br>• Immune-mediated peripheral neuropathies and plexopathies, including chronic inflammatory demyelinating polyneuropathy, multifocal motor neuropathy and polyneuropathies associated with monoclonal gammopathy<br>• Multiple sclerosis<br>• Myasthenia gravis, including Eaton-Lambert syndrome<br>• Narcolepsy<br>• Optic neuritis<br>• Transverse myelitis	• Antisynthetase syndrome<br>• Dermatomyositis<br>• Juvenile chronic arthritis (including Still's disease)<br>• Mixed connective tissue disorder<br>• Polymyalgia rheumatic<br>• Polymyositis<br>• Psoriatic arthropathy<br>• Relapsing polychondritis<br>• Rheumatoid arthritis<br>• Scleroderma, including diffuse systemic form and CREST syndrome<br>• Spondyloarthritis, including ankylosing spondylitis, reactive arthritis (Reiter's Syndrome) and undifferentiated spondyloarthritis<br>• Systemic lupus erythematosus<br>• Systemic sclerosis	• Alopecia areata<br>• Autoimmune bullous skin diseases, including pemphigus, pemphigoid and dermatitis herpetiformis<br>• Cutaneous lupus erythematosus<br>• Erythema nodosum<br>• Morphoea<br>• Lichen planus<br>• Psoriasis<br>• Sweet's syndrome<br>• Vitiligo
**Vasculitides**	**Others**	
• Large vessels vasculitis including: giant cell arteritis such as Takayasu's arteritis and temporal arteritis<br>• Medium sized and/or small vessels vasculitis including: polyarteritis nodosa, Kawasaki's disease, microscopic polyangiitis, Wegener's granulomatosis, Churg-Strauss syndrome (allergic granulomatous angiitis), Buerger's disease thromboangiitis obliterans, necrotizing vasculitis and anti-neutrophil cytoplasmic antibody (ANCA) positive vasculitis (type unspecified), Henoch-Schonlein purpura, Behcet's syndrome, leukocytoclastic vasculitis	• Antiphospholipid syndrome<br>• Autoimmune hemolytic anemia<br>• Autoimmune glomerulonephritis (including IgA nephropathy, glomerulonephritis rapidly progressive, membranous glomerulonephritis, membranoproliferative glomerulonephritis, and mesangioproliferative glomerulonephritis)<br>• Autoimmune myocarditis/cardiomyopathy<br>• Autoimmune thrombocytopenia<br>• Goodpasture syndrome<br>• Idiopathic pulmonary fibrosis<br>• Pernicious anemia<br>• Raynaud's phenomenon<br>• Sarcoidosis<br>• Sjögren's syndrome<br>• Stevens-Johnson syndrome<br>• Uveitis	

This document is proprietary and is to be used only as authorized. No part is to be reproduced, disclosed to others, or quoted without prior written authorization from IDRI.
