# Supplementary material for: Safety and immunogenicity of a thermostable ID93 + GLA-SE tuberculosis vaccine candidate in healthy adults
Source: Nat Commun. 2023 Mar 6;14:1138. doi: 10.1038/s41467-023-36789-2 (PMC9988862; doi:10.1038/s41467-023-36789-2)
Supplement: Supplementary file 3 — Reporting Summary [file 41467_2023_36789_MOESM3_ESM.pdf]

nature portfolio

Corresponding author(s): Christopher Fox

Last updated by author(s): Feb 3, 2023

Reporting Summary

Nature Portfolio wishes to improve the reproducibility of the work that we publish. This form provides structure for consistency and transparency in reporting. For further information on Nature Portfolio policies, see our Editorial Policies and the Editorial Policy Checklist.

Statistics

For all statistical analyses, confirm that the following items are present in the figure legend, table legend, main text, or Methods section.

n/a	Confirmed	
☐	☒	The exact sample size (*n*) for each experimental group/condition, given as a discrete number and unit of measurement
☐	☒	A statement on whether measurements were taken from distinct samples or whether the same sample was measured repeatedly
☐	☒	The statistical test(s) used AND whether they are one- or two-sided<br>*Only common tests should be described solely by name; describe more complex techniques in the Methods section.*
☒	☐	A description of all covariates tested
☐	☒	A description of any assumptions or corrections, such as tests of normality and adjustment for multiple comparisons
☐	☒	A full description of the statistical parameters including central tendency (e.g. means) or other basic estimates (e.g. regression coefficient) AND variation (e.g. standard deviation) or associated estimates of uncertainty (e.g. confidence intervals)
☐	☒	For null hypothesis testing, the test statistic (e.g. *F*, *t*, *r*) with confidence intervals, effect sizes, degrees of freedom and *P* value noted<br>*Give P values as exact values whenever suitable.*
☐	☒	For Bayesian analysis, information on the choice of priors and Markov chain Monte Carlo settings
☒	☐	For hierarchical and complex designs, identification of the appropriate level for tests and full reporting of outcomes
☒	☐	Estimates of effect sizes (e.g. Cohen's *d*, Pearson's *r*), indicating how they were calculated

Our web collection on statistics for biologists contains articles on many of the points above.

Software and code

Policy information about availability of computer code

Data collection	Safety data were collected with DFexplore-DFdiscover version 5 (DF/Net Research, Inc., Seattle, WA). Cell counts for peripheral blood mononuclear cell samples were obtained on a Guava easyCyte (Luminex, Austin, TX), cells were analyzed on an LSRFortessa flow cytometer (BD Biosciences), and cell population counts and frequencies were collected using FlowJo v10.8.1 software (BD Biosciences). Time to positivity of Mycobacteria Growth Indicator Tubes was measured with a Bactec MGIT 320 instrument (BD).
Data analysis	ELISpots were counted and data were analyzed using ImmunoSpot software v. 7.0.22.1 (Cellular Technology Limited, Cleveland, OH). Statistical analyses were conducted using DFexplore-DFdiscover v. 5, MedDRA v. 21.1 (MedDRA MSSO, Herndon, VA), Prism v. 9.3 or higher (GraphPad Software, San Diego, CA), SAS v. 9.4 (SAS Institute, Cary, NC), or R v. 4.03 (The R Foundation, Vienna, Austria).

For manuscripts utilizing custom algorithms or software that are central to the research but not yet described in published literature, software must be made available to editors and reviewers. We strongly encourage code deposition in a community repository (e.g. GitHub). See the Nature Portfolio guidelines for submitting code & software for further information.

Data

Policy information about availability of data

All manuscripts must include a data availability statement. This statement should provide the following information, where applicable:

- Accession codes, unique identifiers, or web links for publicly available datasets
- A description of any restrictions on data availability
- For clinical datasets or third party data, please ensure that the statement adheres to our policy

The datasets generated during and/or analysed during the current study are available from the corresponding author on reasonable request. However, individual participant data will not be available because informed consent did not explicitly include this. The study protocol, study participant disposition, and protocol deviations are included in the Supplementary Information. Source data are provided with this paper.

Human research participants

Policy information about studies involving human research participants and Sex and Gender in Research.

Reporting on sex and gender	This study was inclusive of all healthy adults who met the inclusion/exclusion criteria, regardless of sex. Sex was determined based on self-reporting. After recruitment and screening, 75% of participants enrolled were female, and 25% were male. No sex- or gender-based analyses were performed as the study was not designed with sufficient power to adequately address this aspect.
Population characteristics	Participants were healthy adults. Ages at enrollment ranged from 18 to 48 years with a median of 23 years. Participants' mean body mass index (BMI) was 24.1. Races included 43 (90%) White, 2 (4%) Asian, 2 (4%) mixed, and 1 (2%) Black or African American. Participants were randomized into two treatment groups: one receiving the thermostable single-vial ID93 +GLA-SE vaccine and the other receiving the non-thermostable two-vial ID93+GLA-SE vaccine.
Recruitment	Various methods of recruitment were used to recruit healthy adults from St. Louis, Missouri, and the surrounding area of the clinical site (Saint Louis University Center for Vaccine Development): letters, postcards, e-mails, brochures, posters, media boards, websites, social media, telephone contact, advertisements, and press releases. All forms and mechanisms of recruitment as well as the recruitment materials were approved by the site's Institutional Review Board prior to use. All participants signed informed consent forms.
Ethics oversight	The study protocol, informed consent form, and other study materials were approved by the Saint Louis University Institutional Review Board.

Note that full information on the approval of the study protocol must also be provided in the manuscript.

Field-specific reporting

Please select the one below that is the best fit for your research. If you are not sure, read the appropriate sections before making your selection.

☒ Life sciences ☐ Behavioural & social sciences ☐ Ecological, evolutionary & environmental sciences

For a reference copy of the document with all sections, see nature.com/documents/nr-reporting-summary-flat.pdf

Life sciences study design

All studies must disclose on these points even when the disclosure is negative.

Sample size	This study is a Phase 1, randomized, double-blind clinical trial designed to evaluate the safety, tolerability, and immunogenicity of the single-vial and two-vial presentations of ID93 + GLA-SE. As such, the sample size of n=48 was determined by what is reasonable for a Phase 1 trial and not based on statistical considerations of power, and will allow only preliminary safety and immunogenicity information relevant to progression to larger trials. Based on previous clinical experience with the non-thermostable presentation of ID93 + GLA-SE, we estimated that a sample size of 24 participants per group would allow detection of an increase in systemic adverse reaction frequency of >32.5% in the thermostable presentation group compared to the non-thermostable presentation group at 80% power with a one-sided confidence interval of 95%, and detection of a decrease in serum antibody response rate of >10% in the thermostable presentation group compared to the non-thermostable presentation group at 90% power with a one-sided confidence interval of 95%.
Data exclusions	In rare instances, data were excluded if specific criteria were not met, including high background signal from the corresponding negative control, absence of a negative control value due to user error or insufficient cells, low cell viability or cell count, or off-scale results.
Replication	ELISA and ELISpot assays were performed using duplicate or triplicate wells or plates for intra-experimental technical replication, and mean values were calculated. Technical replicates refer to a given sample being run 2 or 3 times on the sample plate within the same batch run. Data were omitted only in rare circumstances when there was a suspected artifact or quality issue related to operator error, major physical damage to the well, or an off-scale or highly anomalous result inconsistent with other replicates or dilutions. Otherwise, no attempts were made to replicate experiments.

Randomization	Participants were enrolled by randomization into two treatment groups. Participants were assigned a sequential identification number, which was used by statisticians at DF/Net Research (Seattle, WA) to generate the list of randomized treatment assignments. Block randomization of an appropriate size was used to balance enrollment in a 1:1 ratio into each of the two groups.
Blinding	Investigators were blinded to group allocation during data collection and analysis. The study was conducted as a double-blind trial. Participants, investigators, study personnel performing any study-related assessments following study injection, and laboratory personnel performing immunology assays were blinded to treatment assignment.

Reporting for specific materials, systems and methods

We require information from authors about some types of materials, experimental systems and methods used in many studies. Here, indicate whether each material, system or method listed is relevant to your study. If you are not sure if a list item applies to your research, read the appropriate section before selecting a response.

Materials & experimental systems

n/a	Involved in the study	
☐	☒	Antibodies
☒	☐	Eukaryotic cell lines
☒	☐	Palaeontology and archaeology
☒	☐	Animals and other organisms
☐	☒	Clinical data
☒	☐	Dual use research of concern

Methods

n/a	Involved in the study	
☒	☐	ChIP-seq
☐	☒	Flow cytometry
☒	☐	MRI-based neuroimaging

Antibodies

Antibodies used

The following antibodies were used:
- Mouse anti-Human IgG1 Fc Secondary Ab HRP, Clone HP6070, Cat# MH1715, Invitrogen (1000-fold dilution)
- Mouse anti-Human IgG2 Fd Secondary Ab HRP, Clone HP6014, Cat# 05-0520, Invitrogen (500-fold dilution)
- Mouse anti-Human IgG3 (Hinge) Secondary Ab HRP, Clone HP6047, Cat# 53620, Invitrogen (1000-fold dilution)
- Mouse anti-Human IgG4 Fc, Secondary Ab HRP, Clone HP6023, Cat# MH1742, Invitrogen (500-fold dilution)
- Ultra Leaf Human IgG2 Isotype Control Recombinant Antibody, Clone QA16A13, Cat# 403602, BioLegend (1000-fold dilution)
- Ultra Leaf Human IgG4 Isotype Control Recombinant Antibody, Clone QA16A15, Cat# 403702, BioLegend (2350-fold dilution)
- IgG Capture Antibody, Clone MT91/145, Cat# 3850-3-1000, MabTech (100-fold dilution)
- IgA Capture Antibody, Clone MT57, Cat# 3860-3-1000, MabTech (100-fold dilution)
- Biotinylated IgG Detection Ab, Clone MT78/145, Cat# 3850-6-250, MabTech (500-fold dilution)
- Biotinylated IgA Detection Ab, Clone MT20, Cat# 3860-6-250, MabTech (500-fold dilution)
- IFN-γ Mouse anti-human Capture Antibody, Clone 1-D1K, Cat# 3420-3-250, MabTech (100-fold dilution)
- IL-10 Mouse anti-human Capture Antibody, Clone 9D7, Cat# 3430-3-250, MabTech (100-fold dilution)
- IFN-γ Biotinylated Mouse anti-human Detection Ab, Clone 7-B6-1, Cat# 3420-6-250, MabTech (1000-fold dilution)
- IL-10 Biotinylated Mouse anti-human Detection Ab, Clone 12G8, Cat# 3430-6-250, MabTech (1000-fold dilution)
- Biotinylated Anti-Hu-IgA (alpha chain) Ab, Cat# 5260-0027, KPL, Seracare Life Sciences (500-fold dilution)
- Human CD3 R718, Clone SP34-2, Cat# 566955, BD Biosciences (25-fold dilution)
- Human CD4 BUV395, Clone SK3, Cat# 563550, BD Biosciences (25-fold dilution)
- Human CD8 BV711, Clone RPA-T8, Cat# 563677, BD Biosciences (25-fold dilution)
- Human CD14 BV510, Clone M5E2, Cat# 301842, BioLegend (20-fold dilution)
- Human CD45RA APC-Cy7, Clone HI100, Cat# 560674, BD Biosciences (40-fold dilution)
- Human CD56 BV650, Clone HCD56, Cat# 318344, BioLegend (50-fold dilution)
- Human CD154 BUV496, Clone TRAP1, Cat# 750407, BD Biosciences (40-fold dilution)
- Human CD197 (CCR7) BV785, Clone G043H7, Cat# 353230, BioLegend (10-fold dilution)
- Mouse anti-Human CD279 (PD-1) PE-Cy7, Clone eBioJ105, Cat# 25-2799-42, eBioscience (10-fold dilution)
- Human CXCR5 PE-eFluor 610 (PE-Dazzle), Clone MU5UBEE, Cat# 61-9185-42, eBioscience (20-fold dilution)
- Human IL-2 PE, Clone MQ1-17H12, Cat# 559334, BD Biosciences (10-fold dilution)
- Human IL-4 PerCP-Cy5.5, Clone MP4-25D2, Cat# 500822, BioLegend (40-fold dilution)
- Human IL-21 APC, Clone 3A3-N2, Cat# 513008, BioLegend (40-fold dilution)
- Human IFN-γ BV421, Clone B27, Cat# 560371, BD Biosciences (25-fold dilution)
- Human TNF-α FITC, Clone MAb11, Cat# 11-7349-82, eBioscience (40-fold dilution)
- Human CD49d, Clone 9F10, Cat# 16-0499-85, Invitrogen
- Human CD28, Clone CD28.2, Cat# 16-0289-85, eBioscience

Validation

All commercial antibodies used from MabTech had validation statements on the manufacturer's websites. Other manufacturers performed in-house assays to test lots. We also validated commercial antibodies for serum ELISA, ELISpot, and ICS assays by performing initial confirmatory assay runs prior to formal analysis of clinical samples.

Clinical data

Policy information about clinical studies

All manuscripts should comply with the ICMJEguidelines for publication of clinical research and a completedCONSORT checklist must be included with all submissions.

Clinical trial registration	ClinicalTrials.gov NCT03722472
Study protocol	The full study protocol can be found in the Supplementary Information.
Data collection	Participation from human subjects in the study occurred beginning with recruitment from October 29, 2018 to follow-up, ending June 15, 2020. The clinical site, where data was collected, was Saint Louis University Center for Vaccine Development in St. Louis, MO. Participants were compensated $75 per study visit and $10 per telephone call visit.
Outcomes	The primary endpoints included: (1) number of participants experiencing solicited local injection site reactions within 7 days following each study injection, (2) number of participants experiencing solicited systemic reactions within 7 days following each study injection, (3) number of participants spontaneously reporting AEs from Study Day 0 through Study Day 84, and (4) number of SAEs considered related to any of the study injections reported at any point during the study period. General safety was evaluated on Days 0, 7, 56, 63, and 84 for each participant. Blood was assessed for safety laboratory analyses (hematology and serum chemistry) on the day of screening and on Study Days 7 and 63. All participants completed a written participant memory aid that solicited local and systemic reactogenicity AEs for 7 days following each study injection. Unsolicited AEs were recorded through Study Day 84 (28 days following the last study injection). The occurrence of SAEs and the onset of any PIMMCs were recorded throughout the study period (approximately 421 days).<br><br>The secondary endpoints included: (1) proportion of participants with at least a 4-fold increase in IgG antibody responses to ID93 on Study Days 14, 56, 70, 84, and 224 relative to baseline (Study Day 0) as assayed by ELISA; (2) mean fold change from baseline in IgG antibody responses to ID93 on Study Days 14, 56, 70, 84, and 224 relative to baseline (Study Day 0) as assayed by ELISA; (3) number of IFN-γ and IL-10 cytokine-secreting cells in PBMC samples in response to ID93 at Study Days 14, 56, 70, 84, and 224 relative to baseline (Study Day 0) as assayed by ELISpot; and (4) percentage of CD4+ and CD8+ T cells producing two or more cytokines in response to ID93 as measured by ICS with flow cytometry of PBMCs on Study Days 0, 7, 14, 56, 63, 70, 84, and 224.

Flow Cytometry

Plots

Confirm that:

- [x] The axis labels state the marker and fluorochrome used (e.g. CD4-FITC).
- [x] The axis scales are clearly visible. Include numbers along axes only for bottom left plot of group (a 'group' is an analysis of identical markers).
- [x] All plots are contour plots with outliers or pseudocolor plots.
- [x] A numerical value for number of cells or percentage (with statistics) is provided.

Methodology

Sample preparation	Intracellular cytokine staining was performed according to the following procedures. Peripheral blood mononuclear cells (PBMCs) from study participants were thawed and rested in R10 media (RPMI 1640 with 10% FBS, 1% L-glutamine, and 1% penicillin-streptavidin) at a maximum concentration of 2 x 10^6 cells/mL overnight at 37°C and 5% CO2. PBMCs were then resuspended to a concentration of 10 x 10^6 cells/mL and distributed so that 1 x 10^6 cells/mL were each stimulated with 0.25 µg/mL Staphylococcal enterotoxin B (SEB) or 10 µg/mL ID93 or 0.3% DMSO, and a CD28/CD49d co-stimulation cocktail was also added to each. After PBMCs incubated for 2 h at 37°C and 5% CO2, 1X brefeldin A was added to retain secretory proteins (e.g., cytokines and interleukins) intracellularly. Activation continued for an additional 10 h at 37°C and 5% CO2 with cells incubated in a sealed plastic bag containing a damp towel. Cell counts and percent viability were monitored using the Guava EasyCyte flow cytometer (Luminex, Austin, TX). Cells were then stained in multiple stages to detect region/zone-partitioned targets. Cells were stained separately: first with 500-fold dilution of viability dye for 20 min at ambient temperature followed by 5 µg/mL human Fc block for 10 min at ambient temperature (protected from light), and then with an extracellular surface marker cocktail for 20 min at ambient temperature. A complete list of antibodies and reagents used are in the Supplementary Information. Next, cells were permeabilized and fixed with BD CytoFix/CytoPerm (BD Biosciences) for 10 min at ambient temperature (protected from light), followed by 1X Perm/Wash buffer washes. Finally, cells were stained with an intracellular marker cocktail for 30 min at ambient temperature (protected from light), fixed with 1X BD Stabilizing Fixative for 20 min at ambient temperature, and analyzed within 16 h post-staining on the BD LSRFortessa flow cytometer. Data on cell population counts and frequencies were collected using FlowJo v10.8.1 software (BD Biosciences).
Instrument	Cell counts and percent viability were monitored using the Guava EasyCyte flow cytometer (Luminex, Austin, TX) and analyzed on a BD LSRFortessa flow cytometer.
Software	Data on cell population counts and frequencies were collected using FlowJo v10.8.1 software (BD Biosciences).
Cell population abundance	No cell sorting was performed for this assay. All samples were analyzed but not sorted into individual populations.
Gating strategy	Gating limits were determined by using "Fluorescence-minus-multiple" (FMX) and unstained cells. For greater reproducibility and accuracy when defining boundaries between positive and negative cell populations, we used unstained cells and FMX

cocktails (fluorescent marker minus a certain X number of specific antibodies; similar to FMO or "fluorescence marker minus one"). This reduced the degree of subjectivity (inherent variation among analysts) when identifying certain population gate limits. FMX1 was an antibody cocktail containing all antibodies except PD-1, IFN-γ, and TNF-α. The extracellular FMX1 cocktail contained the following antibodies: CD14-BV510, CD56-BV650, CCR7-BV785, CXCR5-PEeFluor610, and CD45RA-APC-H7. The intracellular FMX1 cocktail contained the following antibodies: CD3-R718, CD4-BUV395, CD8-BV711, IL-2-PE, CD154-BUV496, IL-21-APC, and IL-4-PerCP-Cy5.5. The FMX2 cocktail contained all antibodies except CD154, IL-2, IL-4, and IL-21. The extracellular FMX2 cocktail contained the following antibodies: CD14-BV510, CD56-BV650, CCR7-BV785, CXCR5-PEeFluor610, PD-1-PE-Cy7, and CD45RA-APC-H7. The intracellular FMX2 cocktail contained the following antibodies: CD3-R718, CD4-BUV395, CD8-BV711, TNFα-FITC, and IFNγ-V450. These FMXs were specifically generated due to the requirements of the gating strategy and expressive populations of interest. Markers and flourochrome combinations left out of a specific FMX cocktail allowed better separation and clarity of present markers and flourochrome combination in said FMX cocktail. This aided in proper gate placement on these non-primary expressive markers. However, further clarification was needed to provide accurate gating of populations of NK and NK T cells; therefore, a polygonal gating strategy was used for these cells that also included CD3dim cells. See Supplementary Figures 6-10.

☒ Tick this box to confirm that a figure exemplifying the gating strategy is provided in the Supplementary Information.
